# Antileishmanial Activity of Cinnamic Acid Derivatives against *Leishmania infantum*

**DOI:** 10.3390/molecules28062844

**Published:** 2023-03-21

**Authors:** Mayara Castro de Morais, Gisele Alves Medeiros, Fernanda Silva Almeida, Juliana da Câmara Rocha, Yunierkis Perez-Castillo, Tatjana de Souza Lima Keesen, Damião Pergentino de Sousa

**Affiliations:** 1 Department of Pharmaceutical Sciences, Federal University of Paraíba, João Pessoa 58051-900, BP, Brazil; 2 Immunology of Infectious Diseases Laboratory, Department of Cellular and Molecular Biology, Federal University of Paraiba, João Pessoa 58051-900, BP, Brazil; 3 Bio-Cheminformatics Research Group and Area de Ciencias Aplicadas, Facultad de Ingeniería y Ciencias Aplicadas, Universidad de Las Americas, Quito 170503, Ecuador; 4 Postgraduate Program in Bioactive Natural and Synthetic Products, Federal University of Paraíba, João Pessoa 58051-900, BP, Brazil

**Keywords:** cinnamate, cinnamamide, natural product, medicinal plant, antiparasitic activity

## Abstract

*Leishmania infantum* is the etiological agent of visceral leishmaniasis (VL) in South America, the Mediterranean basin, and West and Central Asia. The most affected country, Brazil, reported 4297 VL cases in 2017. *L. infantum* is transmitted by female phlebotomine sand flies during successive blood meals. There are no validated vaccines to prevent the infection and the treatment relies on drugs that often present severe side effects, which justify the efforts to find new antileishmanial drugs. Cinnamic acid derivatives have shown several pharmacological activities, including antiparasitic action. Therefore, in the present study, the biological evaluation of cinnamic acid and thirty-four derivatives against *L. infantum* is reported. The compounds were prepared by several synthesis methods and characterized by spectroscopic techniques and high-resolution mass spectrometry. The results revealed that compound **32** (N-(4-isopropylbenzyl)cinnamamide) was the most potent antileishmanial agent (IC_50_ = 33.71 μM) with the highest selectivity index (SI > 42.46), followed by compound **15** (piperonyl cinnamate) with an IC_50_ = 42.80 μM and SI > 32.86. Compound **32** was slightly less potent and nineteen times more selective for the parasite than amphotericin B (MIC = 3.14 uM; SI = 2.24). In the molecular docking study, the most likely target for the compound in *L. infantum* was aspartyl aminopeptidase, followed by aldehyde dehydrogenase, mitochondrial. The data obtained show the antileishmanial potential of this class of compounds and may be used in the search for new drug candidates against *Leishmania* species.

## 1. Introduction

**Figure 1:**
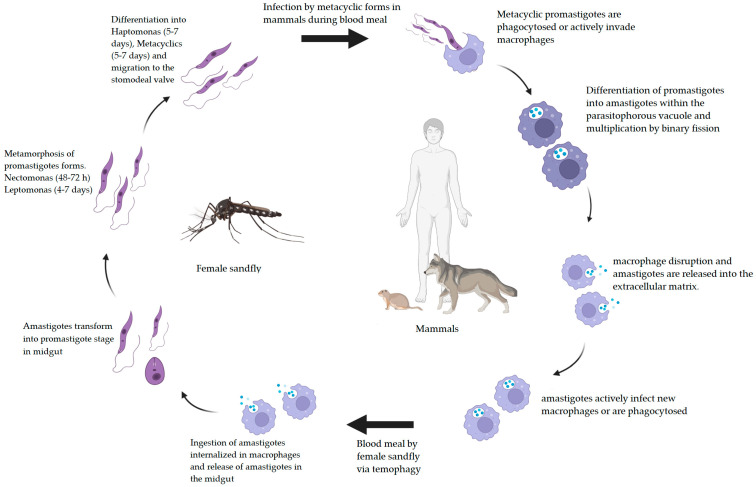
Digenetic life cycle of *Leishmania infantum*, alternating between mammalian and female sandfly hosts.

Leishmaniasis is a public health problem with increasing incidence in the last decade and is considered the second-highest cause of death among infectious tropical diseases [1]. *Leishmania infantum* is a diploid organism, with no distinct sexual cycle [2]. It, like other species of the genus, has a very complex digenetic life cycle involving definitive (vertebrates) and intermediate hosts (insect vectors) with two basic development stages: (1) promastigote, the proliferative form found within the digestive tracts of infected female sandflies; and (2) amastigote, the proliferative form found in many types of mammalian cells [3,4], mainly macrophages, which are the host’s first line of defense (Figure 1) [5]. As a member of the *Leishmania* subgenus, *L. infantum* is considered a suprapilar parasite, with its development restricted to the midgut of sandflies [6,7].

In clinical practice, due to the lack of clinically available vaccines and the difficulty in controlling the vector and host of the disease, the main means of combating leishmaniasis is still pharmacotherapy. However, therapeutic agents are rather limited and consist of pentavalent antimony, amphotericin B (and its lipid formulations), pentamidine, miltefosine, and paromomycin. However, over the years, the rate of resistance to these drugs has increased [8]. Found in many plants, cinnamic acid and its derivatives are often evaluated for their pharmacological activity, and used to model compounds for the development of promising bioactive compounds [9]. Among these synthetic compounds, esters and amides are the most interesting since they exhibit a remarkable range of biological activity. In fact, cinnamic acid and its derivatives present a large range of biological activities, as described, such as anti-inflammatory [10], antioxidant [11], anticancer [12], hypoglycemic [13], cytoprotective [14], antidepressant [15], antibacterial [16], antifungal [17], antimalarial [18], cytotoxic anti-tumor [19], phytotoxic [20,21], and antileishmanial actions. Upon oral administration, cinnamic acid is well absorbed from the gastrointestinal tract and can pass through the blood–brain barrier to also function at the brain level. The compound presents specificity and affinity for a variety of proteins in the human body and can modulate a variety of cell signaling pathways, thus it has potential application to treat diseases [22]. Even still, many studies have been conducted with the aim of producing alternative compounds derived from cinnamic acid and enhancing its pharmacological activity.

In this context, thirty-four chemical derivatives were synthesized from cinnamic acid and its corresponding acid chloride (cinnamoyl chloride) to evaluate the antileishmanial activity and to determine their selectivity against *L. infantum*. Further, a molecular modeling study was also carried out.

## 2. Results

### 2.1. Chemistry

**Scheme 1:**
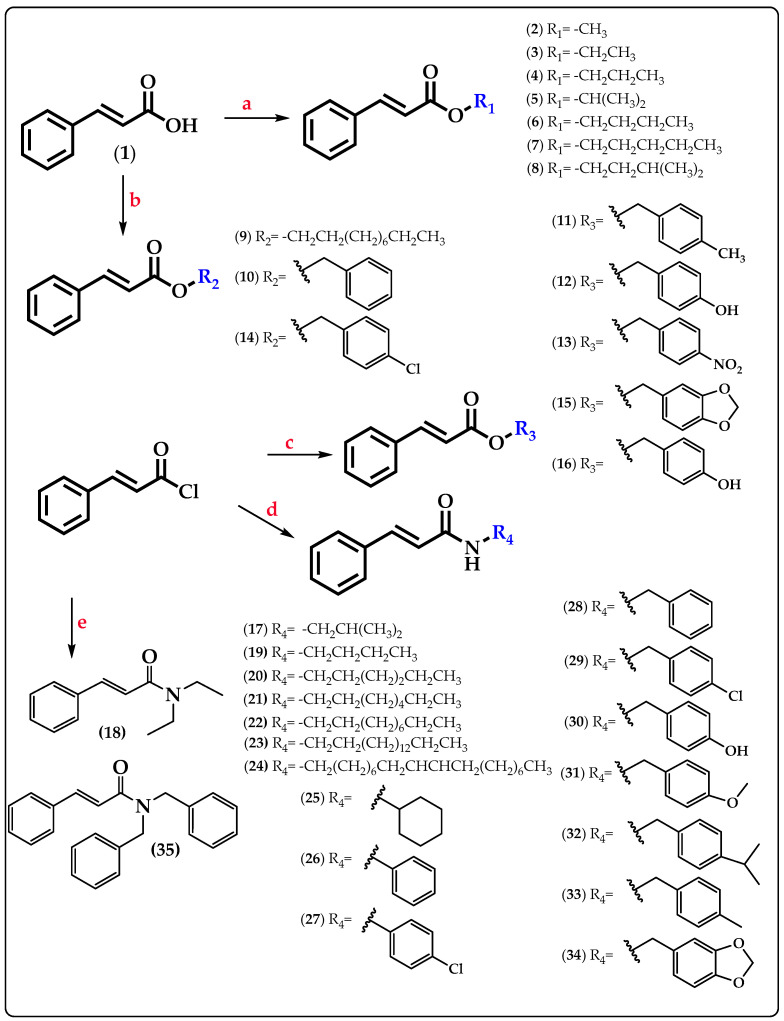
(**a**) ROH, H_2_SO_4_, reflux; (**b**) Et_3_N, RX, acetone, reflux; (**c**) ROH, pyridine, reflux; (**d**) amine, pyridine, reflux; (**e**) diethylamine or dibenzylamine, pyridine, reflux.

The compounds were obtained using three different methods: Fisher’s esterification [23,24], esterification with alkyl and aryl halides [25,26], and the Schotten–Baumann reaction using cinnamoyl chloride and differing though structurally related alcohols or amines, with pyridine [27,28] (Scheme 1). Yields for the thirty-four derivatives ranged from 38% to 91.3%.

In the ^1^H NMR spectra and extensions, it was observed by chemical shifts that cinnamic derivatives possess seven hydrogens in common, five hydrogens belonging to the aromatic ring, and two olefinic hydrogens on the carbonic side chain. For the signals common to all of the analogs, the hydrogen signal of the most unprotected part of the spectrum was that of olefinic hydrogen, which appeared in the form of a doublet close to δH 7.60 ppm, coupled to the neighboring hydrogen that presented a signal in the form of a doublet in a lathe in δH 6.53 ppm, the pair’s link configuration was *trans*, and the coupling constant (*J)* value was close to 16 Hz. There was also a multiplet-like signal with an integral for two hydrogens referring to the hydrogens in the *ortho* position of the aromatic ring near δH 7.44 ppm, and, finally, a signal in the form of a multiplet with an integral for three hydrogens referring to the hydrogens at the *meta* and *para* positions of the aromatic ring, with a chemical displacement of approximately 7.30 ppm. In some molecules with an aromatic ring group, there was an overlap of signals referring to the aromatic rings, making it difficult to observe the olefinic doublet, and impossible to calculate the integrals.

For the ^13^C NMR spectra, by chemical displacements, it was observed that the cinnamic derivatives possessed nine carbons in common. A signal close to δ_C_ 166.0 ppm was attributed to a carbonyl; a signal around δ_C_ 141.3 ppm was attributed as belonging to the olefinic carbon. In addition, there was a signal at δ_C_ 133.0 ppm, attributed to the aromatic carbon adjacent to the olefinic group; the presence of a signal around δC 129.6 ppm belonging to the *meta* carbons of the aromatic ring; a signal appearing around δC 128.8 ppm belonging to the two *ortho* carbons of the aromatic ring; another with a chemical shift of approximately δC 127.8 ppm relative to carbon in the *para* position of the aromatic ring; and, finally, the presence of a signal close to δC 120.3 ppm was assigned to carbon olefinic.

For **30**, an unprecedented compound in the literature, high-resolution mass spectroscopic analysis was also performed. The calculated mass was 254.1165, while the spectra analysis yielded a value of 254.1175.

### 2.2. Antileishmanial Activity

The antileishmanial activity evaluation of the thirty-five cinnamic acid derivatives against the promastigote form of *Leishmania infantum* (Table 1) showed that seventeen compounds are bioactive. N-(4-isopropylbenzyl)cinnamamide (**32**) was the most potent compound (MIC = 33.71 µM), followed by piperonyl cinnamate (**15**) and 3-methyl-4-hydroxybenzyl cinnamate (**16**), with MIC = 42.80 µM and 97.16 µM, respectively.

### 2.3. Selectivity Index

In the evaluation of the selectivity index for the compounds that presented the best results was performed, verifying that none of the compounds presented hemolytic activity at any concentration tested, compound **32** (Table 2) is highlighted.

### 2.4. Molecular Docking

The potential targets of compound **32** in *L. infantum* identified following the approach described in the Methods section are provided in Table 3. The information provided in the table includes the UniProt accession code of each protein, the ID assigned to each one, and a functional description. The predicted potential targets of the compound include a diverse set of proteins and are enriched with the peptidyl-prolyl *cis-trans* isomerase function.

Compound **32** was docked into the proteins listed in Table 3, as described in the Methods section. For PPT, the ATP (PPT-ATP) and glucose (PPT-Glu) binding sites were explored separately. In the case of HDAC, Gold could not find any valid binding mode. This resulted in a total of 22 ligand–receptor complexes selected for further analyses. The detailed results of the molecular docking calculations are provided as Supporting Information in Table S1 and the top-scored conformer per target is presented in Table 4. Overall, the best docking scores were obtained for CYP2, the ATP binding site of PPT, and PAH. On the other hand, the worst scores were predicted for CPC, CYP40, and CYPA.

Despite being widely used, molecular docking has limitations in the accuracy of the predicted ligand–receptor interaction energies. These come from the fact that docking scoring functions use very simplified representations of the intermolecular interactions to be able to process large amounts of chemicals in a reasonable time. For this reason, molecular docking was not used as the selection criterion for the most probable targets of compound **32** in *L. infantum*. Instead, MD simulations were performed and the free energies of binding were estimated from these. Similar approaches have been reported previously for the refining of complexes initially obtained from molecular docking [29,30].

**Figure 2:**
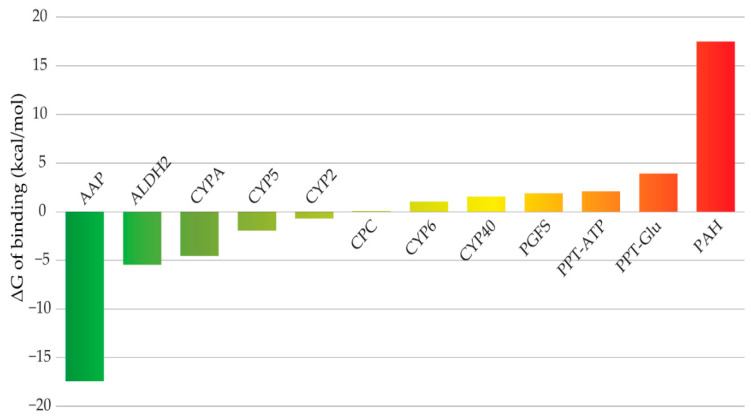
Predicted free energies of the binding of compound **32** to its potential targets in *L. infantum*.

The results of the MM-PBSA calculation for the 22 compound **32**–receptor complexes predicted are given as Supporting Information in Table S2 and summarized in Figure 2. Only the ligand conformation with the best (lowest) free energy per target is presented in Figure 2. According to these results, the most probable target of the compound in *L. infantum* is AAP, followed by ALDH2. Interestingly, three peptidyl-prolyl *cis*-*trans* isomerases are ranked after ALDH2. Given the large difference in the predicted free energies of binding of the ligand to AAP and the second-ranked target, only the complex with AAP was analyzed in detail and considered the most probable one.

**Figure 3:**
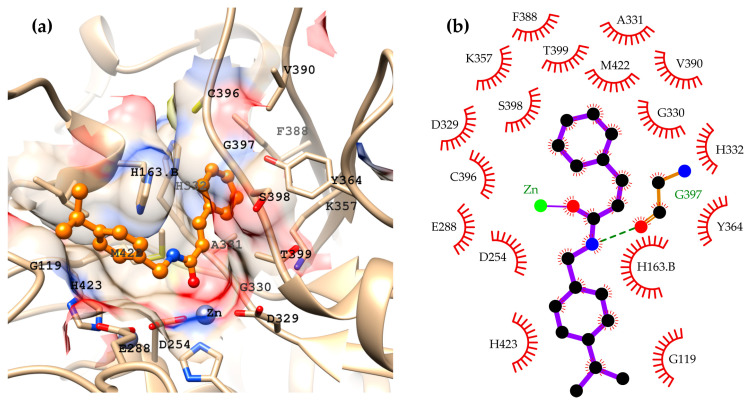
Predicted binding mode of compound **32** to AAP (**a**) and predicted ligand–receptor interactions (**b**). On the left, the ligand is represented in orange balls and sticks, while the receptor is depicted in tan. Nitrogen atoms are colored blue, oxygen atoms red, and sulfur atoms yellow. Only receptor residues interacting with the compound in at least 50% of the examined MD snapshots are labeled in the complex structure and represented in the interactions diagram.

Figure 3 presents the predicted binding mode of compound **32** to AAP (left) along with the observed ligand–receptor interactions (right). For depiction purposes, the structure corresponding to the centroid of the largest cluster obtained from grouping the 100 MD snapshots used for MM-PBSA calculations was employed. The figure was prepared with UCSF Chimera [31], the ligand–receptor interactions diagram was obtained with LigPlot+ [32], and the frequencies of the compound–receptor interactions were analyzed with Cytoscape [33]. Only residues interacting with the ligand in at least 50% of the analyzed MD snapshots are labeled in the figure.

We found no information in the scientific literature about the inhibition of AAP in *Leishmania* parasites. However, metalo-aminopeptidases such as AAP have been explored as molecular targets for the treatment of infectious diseases. The homolog of AAP in *P. falciparum* is a validated molecular target for the development of anti-malarial drug candidates [34]. In *L. donovani* and *L. major*, potent inhibitors have been reported for the leucine aminopeptidase and the methionine aminopeptidases 1 and 2 [35,36,37].

## 3. Discussion

In Table 1, It was observed that cinnamic acid (**1**) presented no bioactivity against the tested parasites, causing no cell deaths at the highest concentration tested. The insertion of a methyl group (**2**) in place of the hydroxyl did not alter the lack of bioactivity. For an analog with an ethyl substituent (**3**) a weak biological action was noted (809.82 μM). The introduction of methylene groups could increase antileishmanial activity. This was observed for the cinnamates with three or four side chain carbons: **4** (propyl group) and **6** (butyl group) (respectively, 253.62 μM and 177.51 μM,). However, compound **7** (pentyl group), with an IC_50_ of 368.66 μM, and **9** (decyl group), with an IC_50_ of 249.80 µg/mL, presented no increases in potency [38,39,40]. Biological activity would be influenced (increased) by the passage of the compound through the biological membranes. However, depending on the chain size, the opposite may occur, since chain size can also imprison the drug, and with water solubility reductions, distribution is also affected [38,39,40]. Lopes et al. (2020) [41] evaluated the antileishmanial action of a series of twelve *p*-coumaric acid derivatives. Of the derivatives tested, eight exhibited antiparasitic activity. Hexyl *p*-coumarate derivative (4.14 ± 0.55 μg/mL; selectivity index (SI = 2.72)) presented the highest antileishmanial potency against the amastigote form of *Leishmania braziliensis.* In the present study, compounds with branched chains, **5** (isopropyl group) and **8** (isopentyl group), presented much lower antileishmanial activity, since it was not possible to determine the IC_50_ of the compound at the maximum concentration tested, highlighting the importance of branching.

Since it does not present substituents on its aromatic ring, compound **10** (benzyl cinnamate) was used as a basis for comparison with the other esters that presented substituents on their aromatic rings. The introduction of bulky groups can protect molecules from enzymatic attack; however, this may also increase or decrease biological activity. Compound **10** presented an IC_50_ of 522.89 μM against the promastigote form, and when compared with the substituted compounds **11** (4-methylbenzyl group), **12** (4-hydroxybenzyl group), and **14** (4-chlorobenzyl group) (IC_50_ = 99.08 μM, 399.01 μM, and 251.71 μM, respectively), significant differences in bioactivity against the promastigote form of the parasite were noted, especially for analog **11** (5.28× more potent), demonstrating that bulky groups can also increase activity. However, compound **13** (4-nitrobenzyl) did not present biological activity, leading us to deduce that the presence of a strong withdrawing group compromises the drug–receptor interaction.

Compound **15**, with a 1,3-dioxolbenzyl group attached to its ring, presented the best results of all the esters (IC_50_ = 42.59 μM). The increase in rigidity and the arrangement of two oxygen atoms in rigid groups on the piperonyl radical contributed to improving the antileishmanial activity, which can be seen by a comparison with analog **16** (presenting a 3-methoxy-3-hydroxybenzyl group), which had its MIC increased in relation to analog **15** (IC_50_ = 97.16 µM). For this biological activity, oxygen atoms in rigid radical structures are important. In certain esters presenting a benzyl ring as a substituent, the insertion of bulky groups with greater rigidity improved antileishmanial activity, possibly explained by the new area opened for induced dipole or hydrophobic bonds between the analog and its respective pharmacological target [39,40,42].

Studies indicate that the salts and esters of acrylate ions (derived from acrylic acid) present antiparasitic activity. They are used by marine phytoplankton for defense. Acrylamides are also derived from acrylic acid. In the cinnamic acid analogs **2**–**35**, the acrylate group is present in the cinnamic esters, and the acrylamide group is present in the amides. Comparing the aliphatic chain amides (**17**–**24**) with the aliphatic chain esters (**2**–**10**), it was found that the change from the acrylate to acrylamide group was decisive for antileishmanial activity and may indicate that cinnamates of the aliphatic chain are more effective than corresponding amides. This was verified between the cinnamate **11** (IC_50_ = 124.6 μM) and the amide **28** (IC_50_ > 400 µg/mL); the same benzyl radical is present in both chemical functions.

Compounds **25**, **26**, and **27** (where the NH is attached directly to the cyclic substituent), and compound **28** (benzyl cinnamamide) were inactive. Thus, CH_2_ spacing influences antiparasitic results in certain compounds. The liposolubility of aniline is lower than benzylamine, due to the presence of a methylene group in the aromatic ring. It was observed (Table 1) that in the *p*-substituted analogs, the introduction of a methyl in the aromatic ring group potentiated biological activity against strains of *L. infantum* [40,43].

The influence on the biological activity of introducing halogen, methyl, methoxyl, hydroxyl, and propyl in the *para* position of the benzyl ring of the substituents was also investigated [40,42]. It was observed that all compounds *para*-substituted on the benzyl ring demonstrated pharmacological activity; **29** (301.05 µM), **30** (410.97 µM), **31** (383.04 µM), and **33** (800.14 μM). The best result was for compound **32**, which contains the *p*-isopropyl-benzyl substituent (33.71 µM). Although the study was with *Leishmania infantum,* the genetic similarity between the species suggests that analog **32** will present antileishmanial activity in other etiological agents that cause leishmaniasis, and can help guide the development of new drugs.

The increase in rigidity and the disposition of two oxygen atoms in rigid groups in the piperonyl radical (**34**) contributed to the antileishmanial activity. Compound **34** presented the second-best result of the collection of amides (IC_50_: 155.16 μM). The tertiary amide (**35**), which presented two benzyl rings (dibenzyl), the bulkiest radical in the collection, presented no activity. It can be assumed that steric hindrance impaired the interaction of the compound with the pharmacological targets.

In Table 2, the compounds with better antileishmania activities showed good selectivity indexes. Compound **32** (SI > 42.46) was approximately nineteen times more selective than the control (Amphoreticin B).

The molecular docking study predicts that compound **32** binds to AAP with its isopropylbenzene group at the entrance of the binding pocket, stacking perpendicularly to the H163 of the neighboring protein monomer and also interacting with G119, E288, and H423. On the other hand, the cinnamamide core points towards the inner cavity. The carbonyl oxygen of the ligand interacts directly with one of the Zn^2+^ ions at the active site, while the nitrogen from amide serves as a hydrogen bond donor for the oxygen atom of G397. It was hypothesized that these two later interactions are highly important for stabilizing the ligand–receptor complex. Finally, the unsubstituted phenyl ring is located at the bottom of the predominantly hydrophobic binding pocket lined by H163, G330, A331, H332, K337, Y364, F388, V390, C396, G397, S398, and T399. 

No reports were found on AAP inhibition in *Leishmania* parasites. However, metallo-aminopeptidases such as AAP have been explored as molecular targets for the treatment of infectious diseases. The AAP homolog in *P. falciparum* is a validated molecular target for the development of antimalarial drug candidates [34]. In *L. donovani* and *L. major*, potent inhibitors have been reported for leucine aminopeptidase and methionine aminopeptidases 1 and 2 [35,37,44].

## 4. Materials and Methods

### 4.1. Chemistry

#### 4.1.1. Chemical Characterization and Reagents

All of the chemical products used during synthesis were from Sigma-Aldrich, St. Louis, MO, USA. ^1^H-NMR and ^13^C-NMR (400 and 100 MHz; 500 and 125 MHz) spectra were respectively recorded on BRUKER-ASCEND (Bruker, Billerica, MA, USA), and VARIAN-RMN-SYSTEM (Varian, Palo Alto, CA, USA) spectrometers. Chemical shifts (δ) are expressed in parts per million (ppm) using TMS as an internal standard. Spin–spin multiplicities are given as *s* (singlet), *brs* (broad singlet), *d* (doublet), *t* (triplet), *q* (quartet), *quint* (quintet), *sex* (sextet), *sept* (septet), and *m* (multiplet). Column adsorption chromatography (CC) was performed on silica gel (Merck 60, 230–400 mesh); analytical TLC was performed on pre-coated silica gel plates (Merck 60 F254). Melting points were determined in a Microquímica apparatus (Microquímica equipamentos LTDA, Model MQAPF 302, Serial No.: 403/18, Palhoça, Brazil) with temperature measurements in the 10 °C to 350 °C range. All reactions were monitored by analytical thin-layer chromatography.

#### 4.1.2. Preparation of Compounds **2**–**8**

Cinnamic acid (0.25 g, 1.69 mmol) and alcohol (50 mL) were added in the presence of sulfuric acid (0.4 mL); this mixture was then heated under reflux until the completion of the reaction (5–24 h), which was verified with single-spot TLC [45]. Spectroscopic data for the compounds in this study are available in the Supplementary Materials [45].

#### 4.1.3. Preparation of Compounds **9**, **10**, and **14**

A mixture of cinnamic acid (0.2 g, 1.35 mmol), triethylamine (0.73 mL), and halide (1.39 mmol) in acetone (16.4 mL) was heated under reflux until a complete reaction (24 h), which was verified with single-spot TLC [46]. Spectroscopic data for the compounds in this study are available in the Supplementary Materials.

#### 4.1.4. Preparation of Compounds **11**–**13** and **15**–**35**

A mixture of cinnamoyl chloride (0.1 g, 0.6 mmol) and the corresponding alcohol or amine (0.6 mmol) in pyridine (1.0 mL) was heated under reflux until a complete reaction (3–24 h), which was verified with single-spot TLC [27]. Spectroscopic data for the compounds in this study are available in the Supplementary Materials.

4-*Hydroxybenzylcinnamamide* (**30**): solid white, yield 38.0% (64.3 mg), M.p.: 188–189 °C, TLC (9:1 hexane/EtOAc), Rf = 0.1 (7:3 Hex: AcOEt), IR vmax (KBr. cm^−1^): 3246, 3066, 2969, 1649, 1614, 1465. ^1^H-NMR (DMSO-d6, 400 MHz): δH 9.12 (*s*, 1H), 8.25 (*t*, *J* = 6.5 Hz, 1H), 7.30–7.27 (*dd*, *J* = 6.4 Hz, 2.0 Hz, 2H), 7.25 (*d*, *J* = 16.0 Hz, 1H), 7.15–7.09 (*m*, 3H), 6.86 (*d*, *J* = 8.4 Hz, 2H), 6.48 (*d*, *J* = 8.4 Hz, 2H), 6.43 (*d*, *J* = 16.0 Hz, 1H). ^13^C-NMR (DMSO-d6, 100 MHz): δC 165.91, 147.15, 145.38, 144.04, 133.91, 130.68. 128.96, 128.59, 128.49, 123.59, 117.45, 44.44. HRMS (MALDI) calculated for C_16_H_15_NO_2_ [M+H]^+^: 254.1165; found: 254.1175.

### 4.2. Antileishmanial Activity

#### 4.2.1. Ethics Statement

All experiments were performed in compliance with the relevant laws and institutional guidelines and in accordance with the ethical standards of the Declaration of Helsinki. Written informed consent was obtained from the patients and was approved by the Ethics Committee of the Federal University of Paraiba (process number: 2.560.067 and CAAE: 82944118.5.0000.5188).

#### 4.2.2. Drugs of Reference

Amphotericin B, which is a drug prescribed for the treatment of leishmaniasis, was used in the present study as a reference antileishmanial drug. A stock solution of amphotericin B was prepared at a concentration of 10 mg/mL in DMSO. Thereafter, this solution was diluted with the appropriate culture media to the concentration needed for the tests, not exceeding a final concentration of 0.5% of DMSO in the test solutions.

#### 4.2.3. *Leishmania* Culture Conditions

The promastigote forms of *Leishmania infantum* (MHOM/BR/2008/RN-05) were cultured in Schneider’s medium, pH 7.0, supplemented with 20% heat-inactivated fetal bovine serum (FBS), 2% male human urine, 100 U/mL of penicillin, and 100 mg/L of streptomycin, and the parasites were maintained in vitro at 26 °C.

#### 4.2.4. Antileishmanial Activity

The inhibition of the promastigote growth assay was performed as previously described by Rodrigues et al. (2015) [47]. Firstly, promastigotes (1 × 10^6^ cells per well) were harvested in the exponential growth phase and were incubated in Schneider’s medium (96-well plates) in the presence and absence of different concentrations (400, 200, 100, 50, 25, 12.5, 6.25, 3.13, 1.56, and 0.7 µg/mL) of ester and amide compounds and the reference drug amphotericin B. The plate was incubated at 26 °C for 72 h in a biological oxygen demand (B.O.D) incubator. The inhibitory effect was evaluated by adding 10 µL of 3-(4,5-dimethylthiazol-2-yl)-2,5-diphenyltetrazolium bromide (5 mg/mL) (MTT; Amresco, Cleveland, OH, USA). After 4 h of incubation, 10% sodium dodecyl sulfate (SDS) was added to dissolve the formazan crystals, and the absorbance at 540 nm was measured using a plate reader (Biosystems model ELx800; Curitiba, PR, Brazil).

#### 4.2.5. Red Blood Cell Lysis Assay

The hemolytic activities of spiro-acridines were determined using human red blood cells from healthy adults (n = 9) according to the method described by Jain et al. (2015) [48]. Briefly, 80 µL of a 5% erythrocyte/phosphate-buffered saline (PBS) suspension was mixed with 20 µL of a series of concentrations (400, 200, 100, 50, 25, 12.5, 6.25, 3.13 µg/mL) of spiro-acridine and reference drugs. After incubation at 37 °C for 1 h, 200 µL of PBS (1.5 mM KH_2_PO_4_, 8.1 mM Na_2_HPO_4_, 136.9 mM NaCl, and 2.6 mM KCl, pH 7.2) was added to stop the hemolysis process, and the samples were centrifuged for 10 min at 1000× *g*. The supernatants were collected, and hemolysis was measured spectrophotometrically at 540 nm. The hemolysis percentage was determined as ((Abs_sam_ − Abs_con_)/(Abs_tot_ − Abs_con_) × 100), where Abs_sam_ is the absorbance of the sample, Abs_con_ is the absorbance of the blank control (without drugs), and Abs_tot_ is the absorbance of total hemolysis (replacing the sample solution with an equal volume of Milli-Q water (Direct-Q; Molsheim, France).

#### 4.2.6. Data Analysis and Statistics

The 50% inhibitory concentration (IC_50_), 50% effective concentration (EC_50_), and 50% hemolytic concentration (HC_50_) values were calculated using the software GraphPadPrism^®^ program (version 6;0; San Diego, CA, USA) for Windows 10. The assays were performed in triplicate and in three independent experiments. For the data obtained in each experiment, the Analysis of Variance (ANOVA) with the post hoc Tukey test was used. Only values with *p* ≤ 0.05 were considered significant.

### 4.3. Modeling Methods

#### 4.3.1. Targets Selection

The potential targets of compound **32** were identified following the previously described homology-based target-fishing approach [49,50]. The first step was to predict probable targets of the compound with the Similarity Ensemble Approach (SEA) web server [51]. Next, the sequences of the identified target proteins were retrieved from the UniProt database and used as a query for a Blast [52] search against the *L. infantum* (taxid: 5671) proteins present in the reference proteins (refseq_protein) database. Blast was performed with its NCBI web interface [53]. Any protein from *L. infantum* identical in at least 40% to any SEA hit and with its sequence covered by the Blast alignment in at least 75% of its length was considered a potential target of compound **32** in the subject parasite.

#### 4.3.2. Molecular Docking

One initial 3D conformation for compound **32** was generated with OpenEye’s Omega [54,55]. Atomic partial charges of type am1bcc were added to this conformation with MolCharge [54]. None of the predicted targets of the compound in *L. infantum* had a 3D structure deposited in the Protein Data Bank database. Thus, homology models were generated for all potential targets with the SwissModel web server [56]. Different homology models were generated for each target protein and the one with the best QMEANDisCo global score per target was selected for additional modeling studies.

Molecular docking calculations were performed with the Gold software [57], as reported in previous publications [36,41]. According to this procedure, hydrogen atoms were added to the receptor and the binding site was defined from the ligands present in the template structures used for homology modeling. Cofactors and metal ions required for protein function were added to the receptor structures using their positioning on the homology model templates if not included in the homology models.

The side chains of the residues directed toward the binding cavity were considered flexible during molecular docking. Primary docking proceeded with the PLP scoring function with the search efficiency parameter of Gold set to 200%. A total of 30 different docking solutions were predicted for each molecular target. All the predicted docking poses were rescored with the ASP, GoldScore, and ChemScore scoring functions of Gold. Afterward, for each target, a consensus scoring approach was applied to select the most probable binding modes of compound **32**. This consensus scoring approach consisted of scaling each scoring value to Z-scores and computing the average Z-score of every predicted pose. All ligand conformations with Z-scores larger than one were selected for additional refinement with molecular dynamics simulations. In the case that no predicted binding pose met the later criterion, only the top-scored ligand conformer was further studied.

#### 4.3.3. Molecular Dynamics Simulations and Estimation of Free Energies of Binding

Molecular dynamics (MD) simulations were performed with Amber 20 1313 following the procedure described in our previous publications [49,58]. The ligand was parameterized with the gaff2 force field and proteins with the ff19SB one. Parameters for cofactors were obtained from the database maintained by the Bryce Group at The University of Manchester (http://amber.manchester.ac.uk/index.html, accessed on 15 November 2022). The parameters for the Zn^2+^ ion and its coordinating residues were retrieved from the Yuan-Ping Pang lab web page (https://www.mayo.edu/research/labs/computer-aided-molecular-design/projects/zinc-protein-simulations-using-cationic-dummy-atom-cada-approach, accessed on 15 November 2022). All the modeled complexes underwent the same energy minimization, heating, equilibration, and production run processes. For each of these, five short MD simulations of length 4 ns were performed, accounting for a total of 20 ns simulation time per complex.

Systems were enclosed in truncated octahedron boxes and solvated with OPC water molecules. The excess charge of each complex was neutralized by adding Na^+^ and Cl^-^ ions at a concentration of 0.15 M according to the procedure described in [59]. Energy minimization was carried out in two stages, with everything except the solvent and counterions restrained during the first of these and with no restraints applied during the second stage. The energy-minimized systems were gradually heated from 0 K to 300 K for 20 ps and the heated systems were equilibrated in the NTP ensemble. Each one of the equilibrated complexes were used as input for the production runs. Initial atomic velocities were randomly initialized before every production run to ensure the exploration of diverse conformational spaces for each complex.

The free energies of binding were estimated from the production runs using the MM-PBSA approach implemented in Amber 20. For a complex, 100 MD snapshots were extracted for MM-PBSA calculations. The selection of the snapshots was performed from all five production runs (20 per trajectory) and evenly drawn from the 1 ns–4 ns time interval. The ionic strength for the calculation of the free energies of binding was set to 0.15 M as during MD simulations.

## 5. Conclusions

In the present study, thirty-four cinnamic acid derivatives were prepared and evaluated against *Leishmania infantum*. Cinnamamide **32** (4-isopropylbenzyl cinnamamide) showed the best antiparasitic action, MIC = 33.71 µM, suggesting that the presence of a bulky alkyl group in the *para* position of the aromatic ring potentiates the antileishmanial action. Cinnamate **15** (piperonyl cinnamate), MIC = 42.80 µM, was the second-best antileishmanial compound. The presence of the dioxymethylene group on the aromatic ring contributed to its better pharmacological profile. Both compounds had good selectivity, SI > 42.46 and SI > 32.86, respectively. Molecular docking studies suggested that the most likely target of compound **32** in *L. infantum* is AAP, followed by ALDH2. Together, these results show that derivatives **32** and **15** can be used as prototypes for researching new drug candidates for the treatment of leishmaniasis.

## Figures and Tables

**Table 1:** Minimum Inhibitory Concentration (MIC) of compounds against *Leishmania (Leishmania) infantum* promastigotes. MIC values are expressed in µg/mL and µM.

Compounds	Radical	X	IC_50_ (µg/mL)	IC_50_ (µM)
**1**	-		>400	-
**2**	Methyl	O	>400	-
**3**	Ethyl	O	142.7	809.82
**4**	Propyl	O	48.25	253.62
**5**	Isopropyl	O	>400	-
**6**	Butyl	O	36.26	177.51
**7**	Pentyl	O	80.48	368.66
**8**	Isopentyl	O	>400	-
**9**	Decyl	O	72.05	249.80
**10**	Benzyl	O	124.6	522.89
**11**	4-methylbenzyl	O	25.0	99.08
**12**	4-hydroxybenzyl	O	92.7	399.01
**13**	4-nitrobenzyl	O	>400	-
**14**	4-chlorobenzyl	O	68.65	251.71
**15**	Piperonyl	O	12.17	42.80
**16**	3-methyl-4-hydroxybenzyl	O	27.55	97.16
**17**	Isobutyl	NH	>400	-
**18**	Dietyl	N	>400	-
**19**	Butyl	NH	>400	-
**20**	Hexyl	NH	>400	-
**21**	Octyl	NH	>400	-
**22**	Decyl	NH	>400	-
**23**	Hexadecyl	NH	>400	-
**24**	Oleyl	NH	>400	-
**25**	Cyclohexyl	NH	>400	-
**26**	Phenyl	NH	>400	-
**27**	4-chlorophenyl	NH	>400	-
**28**	Benzyl	NH	>400	-
**29**	4-chlorobenzyl	NH	81.81	301.05
**30**	4-hydroxybenzyl	NH	104.1	410.97
**31**	4-methoxybenzyl	NH	102.4	383.04
**32**	4-isopropylbenzyl	NH	9.42	33.71
**33**	4-methylbenzyl	NH	201.1	800.14
**34**	Piperonyl	NH	43.65	155.16
**35**	Dibenzyl	N	>400	-
Amphotericin B			2.91	3.14

Antileishmanial evaluation using *L. infantum* promastigotes by the MTT colorimetric method.

**Table 2:** Selectivity index and IC_50_ of the compounds with the best antileishmanial activity.

Compounds	IC50 (µg/mL; µM)	Hemolytic Activity (HC50) (µg/mL; µM)	Selectivity Index (HC50/IC50)
**6**	36.26/177.51	>400/1958.19	>11.03
**11**	25.00/99.08	>400/1585.28	>16
**15**	12.17/42.80	>400/1406.73	>32.86
**16**	27.55/97.16	>400/1410.67	>14.51
**32**	9.42/33.71	>400/1431.42	>42.46
**34**	43.65/155.16	>400/1421.85	>9.16
Amphotericin B	2.91/3.14	6.53/7.04	2.24

**Table 3:** Potential targets of compound **33** in *L. infantum*.

UniProt Accession	ID	Description/Name
A4I6Z4	PGFS	Prostaglandin f2-alpha synthase
A4I1F4	ALDH2	Aldehyde dehydrogenase, mitochondrial
A4I4D6	CPC	Cysteine peptidase C
A4I4V0	AAP	Aspartyl aminopeptidase
A4HZ67	HDAC	Histone deacetylase
A4I1D2	CYPA	Peptidyl-prolyl *cis*-*trans* isomerase (PPIase)
A4HSQ3	CYP2	Peptidyl-prolyl *cis*-*trans* isomerase (PPIase)
A4I698	CYP5	Peptidyl-prolyl *cis*-*trans* isomerase (PPIase)
A4I004	CYP6	Peptidyl-prolyl *cis*-*trans* isomerase (PPIase)
A4IC14	CYP40	Cyclophilin 40
A4HZB2	PPT	Phosphotransferase
A4I3K7	PAH	Phenylalanine-4-hydroxylase

**Table 4:** Docking scores for the top-scored conformer.

Target	PLP ^(a)^	Z_PLP ^(b)^	GS ^(c)^	Z_GS ^(d)^	CS ^(e)^	Z_CS ^(f)^	ASP ^(g)^	Z_ASP ^(h)^	Aggregated Z-Score
PGFS	60.40	−0.39	−7.79	−2.21	27.91	0.36	32.61	0.65	1.52
ALDH2	71.49	0.78	−1.81	−1.83	33.01	1.31	39.00	1.57	1.39
CPC	53.12	−1.17	30.45	0.23	17.93	−1.50	27.70	−0.06	1.15
AAP	70.10	0.64	41.78	0.95	27.45	0.28	29.12	0.15	1.33
CYPA	63.48	−0.07	17.96	−0.57	25.41	−0.10	31.44	0.48	0.88
CYP2	78.44	1.52	30.55	0.24	31.88	1.10	33.74	0.81	2.34
CYP5	70.57	0.69	33.06	0.52	29.43	0.64	32.95	0.70	1.33
CYP6	62.53	−0.17	36.74	0.63	26.03	0.01	25.65	−0.33	1.29
CYP40	51.44	−1.33	23.33	−0.22	21.94	−0.75	24.66	−0.50	0.92
PPT -ATP	57.03	−0.75	36.64	0.62	23.52	−0.46	20.22	−1.14	1.63
PPT-Glu	51.68	−1.32	39.71	0.82	15.07	−2.03	11.16	−2.45	1.17
PAH	79.06	1.59	39.53	0.81	32.07	1.14	29.00	0.13	1.62

^(a)^ PLP score; ^(b)^ PLP Z-score; ^(c)^ GoldScore score; ^(d)^ GoldScore Z-score; ^(e)^ ChemScore score; ^(f)^ ChemScore Z-score; ^(g)^ ASP score; ^(h)^ ASP Z-score.

## Data Availability

Data will be available from the author for correspondence.

## Supplementary Materials

The following supporting information can be downloaded at: https://www.mdpi.com/article/10.3390/molecules28062844/s1, Spectroscopic data of compounds [60,61,62,63,64,65,66,67,68,69,70,71,72,73,74,75,76,77,78,79,80,81,82,83].
