# Supplementary material for: Antileishmanial Activity of Cinnamic Acid Derivatives against Leishmania infantum
Source: Molecules. 2023 Mar 21;28(6):2844. doi: 10.3390/molecules28062844 (PMC10053546; doi:10.3390/molecules28062844)
Supplement: Supplementary file 1 [file molecules-28-02844-s001.zip › molecules-2267907-supplementary.pdf]

Article

Leishmanicidal activity of cinnamic acid derivatives against *Leishmania infantum*

Mayara Castro de Morais [1], Gisele Alves Medeiros [1], Fernanda Silva Almeida [2], Juliana da Câmara Rocha [2], Yunierkis Perez-Castillo [3], Tatjana de Souza Lima Keesen [2] and Damião Pergentino de Sousa [1,4,*]

1 Department of Pharmaceutical Sciences, Federal University of Paraíba, João Pessoa 58051-900, BP, Brazil
2 Immunology of Infectious Diseases Laboratory, Department of Cellular and Molecular Biology, Federal University of Paraiba, João Pessoa 58051-900, BP, Brazil
3 Bio-Cheminformatics Research Group and Area de Ciencias Aplicadas, Facultad de Ingeniería y Ciencias Aplicadas, Universidad de Las Americas, Quito 170503, Ecuador
4 Postgraduate Program in Bioactive Natural and Synthetic Products, Federal University of Paraíba, João Pessoa 58051-900, Brazil
* Correspondence: damiao_desousa@yahoo.com.br

Abstract: *Leishmania infantum* is the etiological agent of visceral leishmaniasis (VL) in South America, the Mediterranean basin, and West and Central Asia. The most affected country, Brazil, reported 4297 VL cases in 2017. *L. infantum* is transmitted by female phlebotomine sand flies during successive blood meals. There are no validated vaccines to prevent the infection and the treatment relies on drugs that often present severe side effects, which justify the efforts to find new antileishmanial drugs. Cinnamic acid derivatives have shown several pharmacological activities, including antiparasitic action. Therefore, in the present study, the biological evaluation of cinnamic acid and thirty-four derivatives against *L. infantum* is reported. The compounds were prepared by several synthesis methods and characterized by spectroscopic techniques and high-resolution mass spectrometry. The results revealed that compound **32** (N-(4-isopropylbenzyl)cinnamamide) was the most potent antileishmanial agent (IC_{50} = 33.71 μM) with the highest selectivity index (SI > 42.46), followed by compound **15** (piperonyl cinnamate) with an IC_{50} = 42.80 μM and SI > 32.86. Compound **32** was slightly less potent and nineteen times more selective for the parasite than amphotericin B (MIC = 3.14 uM; SI = 2.24). In the molecular docking study, the most likely target for the compound in *L. infantum* was aspartyl aminopeptidase, followed by aldehyde dehydrogenase, mitochondrial. The data obtained show the antileishmanial potential of this class of compounds and may be used in the search for new drug candidates against *Leishmania* species.

Keywords: cinnamate; cinnamamide; natural product; medicinal plant; antiparasitic activity

Methyl cinnamate (**2**): Amber amorphous solid; Yield 87.4% (239.3 mg, 1.47 mmol); $C_{10}H_{10}O_2$; 162.19 g/mol; m.p.: 31–33°C [1]; TLC (hexane); R_f = 0.20; IR υmax (KBr, cm^{-1}): 3063, 3030, 2946, 1710, 1639, 1579, 1450, 1309, 1176; ^{1}H-NMR ($CDCl_3$, 200 MHz): δ_H 7.65 (1H; *d*; J = 16.0 Hz), 7.46–7.45 (2H; *m*), 7.33–7.31 (3H; *m*), 6.41 (1H; *d*; J = 16.0 Hz), 3.75 (3H; *s*); ^{13}C-NMR ($CDCl_3$, 100 MHz,): δ_C 167.31, 144.81, 134.37, 130.27, 128.86, 128.07, 117.80, 51.59 [1,2].

Ethyl cinnamate (**3**): Yellow oil; Yield 90.7% (269.53 mg, 1.52 mmol); $C_{11}H_{12}O_2$; 176.22 g/mol; TLC (hexane); R_f = 0.22; IR υmax (KBr, cm^{-1}): 3067, 3032, 2930, 1715, 1638, 1578, 1451, 1312, 1173, 768; ^{1}H-NMR ($CDCl_3$, 500 MHz): δ_H 7.67 (1H; *d*; J = 16.5 Hz), 7.46–7.45 (2H; *m*), 7.32–7.30 (3H; *m*), 6.42 (1H; *d*; J = 16.5 Hz), 4.23 (2H; *q*; J = 7.0 Hz), 1.34 (3H; *t*; J = 7.0 Hz); ^{13}C-NMR ($CDCl_3$, 100 MHz,): δ_C 166.83, 144.51, 134.43, 130.17, 128.82, 128.02, 118.24, 60.38, 14.28 [1,2].

Propyl cinnamate (**4**): Yellow oil; Yield 90.7% (144.4 mg, 0.78 mmol); $C_{12}H_{14}O_2$; 190.24 g/mol; TLC (hexane); R_f = 0.28; IR υmax (KBr, cm^{-1}): 3064, 3032, 2969, 1714, 1639, 1579, 1450, 1313, 1174, 768; ^{1}H-NMR ($CDCl_3$, 500 MHz): δ_H 7.69 (1H; *d*; J = 16.0 Hz), 7.53–7.51 (2H; *m*), 7.38–7.37 (3H; *m*), 6.45 (1H; *d*; J = 16.0 Hz), 4.16 (2H; *t*; J = 7.0 Hz), 1.73 (2H; *sex*; J = 7.0 Hz), 0.99 (3H; *t*, J = 7.5 Hz); ^{13}C-NMR ($CDCl_3$, 125 MHz,): δ_C 167.07, 144.58, 134.51, 130.22, 128.89, 128.07, 118.31, 66.14, 22.13, 10.47 [1-3].
Isopropyl cinnamate (**5**): Yellow oil; Yield 83.1% (133.3 mg, 0.70 mmol); $C_{12}H_{14}O_2$; 190.24 g/mol; TLC (hexane); R_f = 0.34; IR υmax (KBr, cm^{-1}): 3063, 2981, 1710, 1639, 1579, 1450, 1309, 1176, 768; ^{1}H-NMR ($CDCl_3$, 500 MHz): δ_H 7.64 (1H; *d*; J = 16.0 Hz), 7.44–7.42 (2H; *m*), 7.30–7.28 (3H, *m*), 6.37 (1H; *d*; J = 16.0 Hz), 5.10 (1H; *septed*; J = 6.0 Hz), 1.26 (6H; *d*; J = 6.0 Hz); C-NMR ($CDCl_3$, 125 MHz,): δ_C 166.16, 144.06, 134.35, 129.93, 128.66, 127.82, 118.62, 67.32, 21.74 [1,3,4].
Butyl cinnamate (**6**): Yellow oil; Yield 89.3% (153.8 mg, 0.75 mmol); $C_{13}H_{16}O_2$; 204.27 g/mol; TLC (hexane); R_f = 0.2; IR υmax (KBr, cm^{-1}): 3063, 3030, 2961, 1714, 1639, 1579, 1451, 1311, 1172, 768; ^{1}H-NMR ($CDCl_3$, 400 MHz): δ_H 7.69 (1H; *d*; J = 16.0 Hz), 7.53–7.51 (2H; *m*), 7.39–7.37 (3H; *m*), 6.44 (1H; *d*; J = 16.0 Hz), 4.21 (2H; *t*; J = 6.8 Hz), 1.70 (2H; *q*; J = 6.8 Hz), 1.44 (2H; J = 7.2 Hz), 0.97 (3H; *t*; J = 7.2 Hz); ^{13}C-NMR ($CDCl_3$, 100 MHz,): δ_C 167.12, 144.57, 134.50, 130.21, 128.88, 128.06, 118.32, 64.45, 30.88, 19.22, 13.76 [1,4].
Pentyl cinnamate (**7**): Yellow oil; Yield 68.9% (126.9 mg, 0.58 mmol); $C_{14}H_{18}O_2$; 218.30 g/mol; TLC (hexane); R_f = 0.22; IR υmax (KBr, cm^{-1}): 3065, 3032, 2959, 1714, 1639, 1579, 1450, 1311, 1171, 767; ^{1}H-NMR ($CDCl_3$, 400 MHz): δ_H 7.68 (1H; *d*; J = 16.0 Hz), 7.52–7.50 (2H; *m*), 7.37–7.35 (3H; *m*), 6.43 (1H; *d*; J = 16.0 Hz), 4.20 (2H; *t*; J = 6.8 Hz), 1.71 (2H; *q*; J= 6.8 Hz), 1.41–1.36 (4H; *m*), 0.93 (3H; J = 7.2 Hz); ^{13}C-NMR ($CDCl_3$, 100 MHz,): δ_C 167.08, 144.57, 134.53, 130.23, 128.90, 128.08, 118.35, 64.73, 28.49, 28.18, 22.41, 14.02 [1,5,6].
Isopentyl cinnamate (**8**): Yellow oil; Yield 76.1% (140.2 mg, 0.64 mmol); $C_{14}H_{18}O_2$; 218.30 g/mol; TLC (hexane); R_f = 0.2; IR υmax (KBr, cm^{-1}): 3064, 3034, 2960, 1714, 1639, 1579, 1451, 1311, 1168, 767; ^{1}H-NMR ($CDCl_3$, 400 MHz): δ_H.7.68 (1H; *d*; J = 16.0 Hz), 7.53–7.51 (2H; *m*), 7.38–7.36 (3H; *m*), 6.44 (1H; *d*; J = 16.0 Hz), 4.24 (2H; *t*; J = 6.8 Hz), 1.80–1.70 (1H; *m*), 1.60 (2H; *q*; J = 6.8 Hz), 0.96 (3H; *d*; J = 6.4 Hz); ^{13}C-NMR ($CDCl_3$, 100 MHz,): δ_C 167.24, 144.62, 134.65, 130.22, 129.02, 128.16, 118.33, 63.32, 37.66, 25.20, 22.69 [1].
Decyl cinnamate (**9**): Yellow oil; Yield: 47.9% (105.9 mg); $C_{19}H_{28}O_2$; 288.43 g/mol; TLC (9:1 Hex:AcOEt); R_f = 0.52; IR υmax (KBr, cm^{-1}): 3051, 3030, 2932, 1703, 1640, 1593, 1440, 1310, 1187; ^{1}H-NMR ($CDCl_3$, 500 MHz): 7.68 (*d*, J = 16.0 Hz, 1H); 7.50 – 7.49 (*m*, 2H); 7.36 – 7.34 (*m*, 3H); 6.43 (*d*, J = 16.0 Hz); 4.19 (*t*, J = 7.0 Hz, 2H); 1.69 (*q*, J = 7.0 Hz, 2H); 1.42-1.37 (*m*, 2H); 1.36-1.27 (*m*, 12H), 0.88 (*t*, J= 7.0 Hz, 3H); ^{13}C-NMR ($CDCl_3$, 125 MHz,): δ_C 167.03, 144.54, 134.51, 130.19, 128.85, 128.05, 118.32, 64.71, 31.93, 29.58, 29.35, 29.35, 29.33, 28.78, 26.02, 22.71, 14.13 [1].
Benzyl cinnamate (**10**): Yellow oil; Yield 52.9% (85.1 mg, 0.36 mmol); $C_{16}H_{14}O_2$; 238.29 g/mol; TLC (hexane); R_f = 0.3; IR υmax (KBr, cm^{-1}): 3066, 3032, 2921, 1717, 1637, 1578, 1450, 1309, 1163, 767; ^{1}H-NMR ($CDCl_3$, 400 MHz): δ_H 7.75 (*d*, *J* = 16.0 Hz, 1H); 7.54 – 7.53 (*m*, 2H); 7.43 – 7.38 (*m*, 8H, H-3, H-4, H-5, H-2′, H-3′, H-4′, H-5′, H-6′); 6.51 (*d*, *J* = 16.0 Hz, 1H); 5.27 (*s*, 2H); ^{13}C-NMR ($CDCl_3$, 100 MHz,): δ_C 166.03, 145.06, 135.28, 132.74, 130.60, 129.97, 128.94, 128.49, 128.44, 117.72, 64.83 [1,5–7].
4-Methylbenzyl cinnamate (**11**): White solid; Yield 63.8% (108.6 mg, 0.43 mmol); m.p.: 40–44°C [1]; $C_{17}H_{16}O_2$; 252.30 g/mol; TLC (9:1 hexane/EtOAc); R_f = 0.52; IR υmax (KBr, cm^{-1}): 3051, 3027, 2922, 1704, 1640, 1574, 1448, 1310, 1187, 803, 768; ^{1}H-NMR ($CDCl_3$, 400 MHz): δ_H 7.76 (*d*, J = 16.0 Hz, 1H); 7.54 – 7.51 (*m*, 2H); 7.39 – 7.38 (*m*, 3H), 7.33 (*d*, J = 8.0 Hz, 2H),

7.21 (*d*, J = 8.0 Hz, 2H), 6.48 (*d*, J = 16.0 Hz, 1H), 5.32 (*s*, 2H), 2.38 (*s*, 3H), ^{13}C-NMR ($CDCl_3$, 100 MHz,): δ_C 166.10, 145.22, 138.54, 134.24, 133.46, 130.40, 129.66, 129.02, 128.61, 128.26, 118.10, 66.47, 21.75 [1,2,5].

4-Hydroxybenzyl cinnamate (**12**): Yellow oil; Yield 70.0% (110.6 mg); $C_{16}H_{14}O_3$; 218.30 g/mol; TLC (7:3 hexane/EtOAc); R_f = 0.2; IR υmax (KBr, cm^{-1}): 3064, 3028, 2970, 1683, 1632, 1552, 1465, 1326; ^{1}H-NMR ($CDCl_3$, 400 MHz): δ_H 7.80 (1H; *d*; J = 16.0 Hz), 7.74–7.72 (2H; *m*), 7.67–7.63 (3H; *m*), 7.45 (*d*; J = 8.0 Hz, 2H), 7.36-7.35 (2H; *m*), 6.65 (1H; *d*; J = 16.0 Hz), 5.19 (2H; *s*); ^{13}C-NMR ($CDCl_3$, 100 MHz,): δ_C 167.35, 156.16, 145.40, 134.43, 130.49, 129.01, 128.24 , 128.09 , 118.02, 115.63, 66.49 [1,8].

4-Nitrobenzyl cinnamate (**13**): White crystal solid; Yield 62.0% (118.6 mg, 0.42 mmol); m.p.: 116–117°C [2]; $C_{16}H_{13}NO_2$; 283.28 g/mol; TLC (9:1 hexane/EtOAc); R_f= 0.28; IR υmax (KBr, cm^{-1}): 3083, 3067, 2968, 1709, 1632, 1606, 1450, 1517, 1345, 1312, 1158, 859, 749; ^{1}H-NMR (DMSO-d_6, 400 MHz): δ_H 8.23-8.15 (*m*, 2H), 7.71 (1H; *s*), 7.70–7.62 (4H; *m*), 7.39–7.35 (3H; *m*), 6.68 (1H; *d*; J = 16.0 Hz), 5.32 (2H; *s*); ^{13}C-NMR (DMSO-d_6, 100 MHz,): δ_C 165.93, 147.11, 145.38, 144.05, 133.91, 130.68, 128.96, 128.58*, 128.49*, 123.64, 117.45, 64.44 [1,9].

* interchangeable

4-Chlorobenzyl cinnamate (**14**): White solid; Yield 43.1% (158.6 mg, 0.58 mmol); m.p.: 62–63°C [1]; $C_{16}H_{13}ClO_2$; 272.73 g/mol; TLC (9:1 hexane/EtOAc); R_f = 0.48; IR υmax (KBr, cm^{-1}): 3065, 024, 2963, 1710, 1639, 1594, 1446, 1312, 1163, 1012, 801, 770; ^{1}H-NMR ($CDCl_3$, 400 MHz): δ_H 7.74 (1H; *d*; J = 16.0 Hz), 7.53–7.51 (2H; *m*), 7.39–7.38 (3H, *m*), 7.35 (4H; *s*), 6.48 (1H; *d*; J = 16.0 Hz), 5.21 (2H; *s*); ^{13}C-NMR ($CDCl_3$, 100 MHz,): δ_C 166.78, 145.57, 134.72, 134.39, 134.27, 130.57, 129.77, 129.04, 128.91, 128.26, 117.74, 65.62 [1,4,10].

Piperonyl cinnamate (**15**): White oil; Yield 82.01% (170.9 mg); $C_{17}H_{14}O_4$; 282.30 g/mol; TLC (9:1 hexane/EtOAc); R_f= 0.37; IR υmax (KBr, cm^{-1}): 3055, 3024, 2959, 1710, 1639, 1594, 1446, 1312, 1163; ^{1}H-NMR (DMSO-d_6, 400 MHz): δH 7.69-7.68 (*m*, 2H), 7.63 (*d*, J= 16.0 Hz, 1H), 7.39- 7.36 (*m*, 3H), 6,97 (*sl*, 1H); 6.90-6.86 (*m*, 2H), 6.64 (*d*, J=16.0 Hz, 1H), 5.99 (*s*, 2H), 5.08 (*s*, 2H); ^{13}C-NMR (DMSO-d_6, 100 MHz,): δ_C 166.12, 147.37, 147.19, 144.81, 134.02, 130.56, 129.86, 128.95, 128.44, 122.31, 117.96, 109.01, 108.19, 101.12, 65.68 [1,2].

4-hydroxy-3-methoxy-benzyl cinnamate (**16**): Yellow oil, Yield 53%, IV υmax (KBr. cm^{-1}): 3317, 3034, 2981, 2935, 1701, 1641, 1610, 1452, 1246, 1167. ^{1}H-NMR (400 MHz. $CDCl_3$): 7.72 (*d*, *J* = 16.0 Hz, 1H), 7.52 – 7.50 (*m*, 2H), 7.41 - 7.36 (*m*, 3H), 6.96 – 6.89 (*m*, 3H.), 6.47 (*d*, *J* = 16.0 Hz, 1H), 5.16 (*s*, 2H), 3.91 (*s*, 3H), ^{13}C-NMR (100 MHz. $CDCl_3$): 167.04 , 145.67 , 145.99 , 145.24 , 134.15 , 130.48 , 129.03 , 128.23 , 128.05 , 122.33 , 118.12 , 114.53 , 111.51 , 66.76 , 56.11 [11].

Isobutylcinnamamide (**17**): Amorphous solid yellow, Yield 83.7% (99.9 mg), M.p.: 107-110°C [12], TLC (7:3 hexane/EtOAc), Rf = 0.5, IR υmax (KBr, cm^{-1}): 3271, 3082, 2961, 1655, 1618 - 1469. ^{1}H-NMR (DMSO-d_6, 400 MHz): δH 8.23 (*t*, J=7.5 Hz, 1H), 7.55- 7.52 (*m*, 2H), 7.41-7.32 (*m*, 4H), 6.73 (*d*, J= 16.0 Hz, 1H), 2.59 (*d*, J= 6.8 Hz, 2H), 1.76 (*sept*, J= 6.8 Hz, 1H), 0.94 (*d*, J= 6.4 Hz, 6H). ^{13}C-NMR (DMSO-d_6, 100 MHz): δC 165.08, 138.40, 135.06, 129.39, 128.96, 127.52, 122.54, 46.31, 28.20, 20.24, 19.84 [13].

Diethylcinnamamide (**18**): Solid amorphous yellow, Yield 70% (86.7 mg), M.p.: 65-66° C [14], TLC (9:1 hexane/EtOAc), Rf = 0.4 (7: 3 Hex: AcOEt), IR υmax (KBr, cm^{-1}): 3028, 2968, 1648, 1595, 1461. ^{1}H-NMR ($CDCl_3$, 500 MHz): δH 7.69 (*d*, J = 15.5 Hz, 1H), 7.51-7.50 (*m*, H = 2), 7.37-7.32 (*m*, 3H), 6.82 (*d*, J = 15.0 Hz. 1H), 3.49-3.43 (*m*, 4H), 1.27 (t, J=7.0 Hz, 3H), 1.24 (t, J=7.0 Hz, 3H). ^{13}C-NMR ($CDCl_3$. 125 MHz): δC 165.81, 142.35, 135.58, 129.50, 128.82, 127.80, 117.89, 42.39, 41.17, 15.12, 13.27 [15].

Butylcinnamamide (**19**): Solid amorphous white, Yield 73.01% (74.40 mg), M.p.: 85-88º C [16], TLC (9:1 hexane/EtOAc), Rf = 0.49 (7:3 Hex:AcOEt), IR υmax (KBr, cm^{-1}): 3375, 3060, 2924, 1657, 1614, 1467. ^{1}H-NMR (DMSO-d6, 500 MHz): δH 8.01 (*t*, J= 7.5 Hz, 1H), 7.43-7.48 (*m*, 2H), 7.33- 7.25 (*m*, 4H), 6.55 (*d*, J= 15.0 Hz, 1H), 3.08 (*q*, J= 6.0 Hz, 2H), 1.36 (*quint*, J= 7.0 Hz, 2H), 1.18-1.15 (*m*, 2H), 0.76 (*t*, J= 7.0 Hz, 3H). ^{13}C-NMR (DMSO-d6, 125 MHz): δC 164.84, 138.33, 134.99, 129.35, 128.96, 127.45, 122.41, 38.71, 31.25, 22.09, 13.90 [16].
Hexylcinnamamide (**20**): White oil, Yield 69.3% (75.02 mg), TLC (9:1 hexane/EtOAc), Rf = 0.52 (7:3 Hex:AcOEt), IR υmax (KBr, cm^{-1}): 3311, 3059, 2924, 1655, 1618, 1465. ^{1}H-NMR (DMSO-d6, 400 MHz): δH 7.67 (*d*, J = 16.0 Hz, 1H), 7.50-7.49 (*m*, 2H), 7.38- 7.34 (*m*, 3H), 6.57 (*d*, J = 16.0 Hz, 1H), 4.19 (*t*, J= 7.0 Hz, 2H), 1.69 (*quint*, J= 7.5 Hz, 2H), 1.39-1.27 (*m*, 6H), 0.88 (*t*, J=7.0 Hz, 3H). ^{13}C-NMR (DMSO-d6. 100 MHz): δC 167.03, 144.54 , 134.51 , 130.19 , 128.85 , 128.05 , 118.32 , 31.93 , 29.58 , 29.33 , 26.02 , 22.71 , 14.13 [17].
Octylcinnamamide (**21**): Solid amorphous white, Yield 87.5% (135.39 mg), M.p.: 79-82 °C [17], TLC (9:1 hexane/EtOAc), Rf = 0.58 (7:3 Hex:AcOEt), IR vmax (KBr. cm^{-1}): 3284, 3063, 2924, 1651, 1610, 1466. ^{1}H-NMR (DMSO-d6, 500 MHz): δH 7.62 (d, J= 16.0 Hz, 1H), 7.51-7.46 (*m*, 2H). 7.37- 7.30 (*m*, 3H), 6.41 (*d*, J=16.0 Hz, 1H), 3.38 (*quart*. J = 6.5 Hz, 2H), 1.56 (*quint*, J = 6.5 Hz, 2H), 1.33-1.23 (*m*, 10H), 0.87 (*t*, J= 6.5 Hz, 3H). ^{13}C-NMR (DMSO-d6, 125 MHz): δC 166.01 , 140.84 , 135.05 , 129.67 , 128.89 , 127.85 , 121.02 , 39.96 , 31.91 , 29.81 , 29.41 , 29.33 , 27.12 , 22.75 , 14.20 [17].
Decylcinnamamide (**22**): Solid amorphous white, Yield 42.6 % (95.0 mg), M.p.: 178-179 °C, TLC (9:1 hexane/EtOAc), Rf = 0.42 (7:3 Hex:AcOEt), IR vmax (KBr. cm^{-1}): 3302, 3034, 2924, 1651, 1619, 1534, 1467. ^{1}H-NMR ($CDCl_3$. 500 MHz): δH 8.04 (*t*, J= 6.0 Hz, 1H), 7.47-7.44 (*m*, 2H), 7.33- 7.25 (*m*, 4H), 6.55 (*d*, J=15.0 Hz, 1H), 3.08 (*quart*, J= 7.0 Hz, 2H), 1.36 (*quint*, J=7.0 Hz, 2H), 1.22- 1.13 (*m*, 14H), 0.76 (*t*, J= 7.0 Hz, 3H). ^{13}C-NMR ($CDCl_3$, 125 MHz):δC 164.84 , 138.35 , 134.99 , 129.33 , 128.90 , 127.45 , 122.41 , 38.71 , 31.25 , 29.13 , 28.74 , 28.66, 28.14 , 22.09 , 13.93 [18].
Hexadecylcinnamamide (**23**): Solid amorphous white, Yield 62.6 % (115.0 mg), M.p.: 180-182 °C [18], TLC (9:1 hexane/EtOAc), Rf = 0.52 (7:3 Hex:AcOEt), IR vmax (KBr. cm-1): 3311, 3059, 2954, 1655, 1618, 1534, 1467. ^{1}H-NMR ($CDCl_3$, 500 MHz): δH 7.61 (*d*, J=15.5 Hz, 1H), 7.49-7.47 (*m*. 2H), 7.35-7.32 (*m*, 3H), 6.41 (*d*, J=15.5 Hz, 1H), 3.37 (*quart*, J= 7.5 Hz, 2H), 1.57 (*quint*, J= 7.0 Hz, 2H), 1.35- 1.24 (*m*, 26H), 0.88 (*t*, J= 7.0 Hz, 3H). ^{13}C-NMR ($CDCl_3$. 125 MHz): δC 166.03, 140.86 , 134.99, 129.66, 128.89, 127.85, 121.02, 39.96, 32.04, 29.82, 29.78, 29.72, 22.81, 14.24 [18].
Oleylcinnamamide (**24**):Yellow oil, Yield 52.1% (110.5 mg), TLC (9:1 hexane/EtOAc), Rf = 0.65 (7:3 Hex:AcOEt), IR vmax (KBr. cm-1): 3397, 3063, 2923, 1641, 1593, 1539, 1427. ^{1}H-NMR ($CDCl_3$. 500 MHz): δH 8.01 (*t*, J= 6.0 Hz, 1H), 7.50- 7.48 (*m*, 2H), 7.37-7.29 (*m*, 4H), 6.57 (*d*, J=16.0 Hz, 1H), 5.29- 5.25 (*m*, 2H), 3.11 (*quart*, J= 6.5 Hz, 2H), 1.94- 1.87 (*m*, 4H), 1.39 (*quint*, J= 6.5 Hz, 2H), 1.24-1.17 (*m*, 22H), 0.79 (*t*, J= 6.5 Hz, 3H); ^{13}C-NMR ($CDCl_3$. 125 MHz): δC 164.81 , 138.34 , 134.99 , 129.60, 129.30, 128.88 , 127.42, 122.37 , 38.70 , 31.94, 29.14-28.60, 26.60 , 26.58, 26.49 , 22.09, 13.90 [19].
Cyclohexylcinnamamide (**25**): Yellow solid amorphous, Yield 51.3% (66.08 mg), M.p.: 85-87 °C [20], TLC (9:1 hexane/EtOAc), Rf = 0.7 (7:3 Hex:AcOEt), IR vmax (KBr. cm-1): 3278, 3080, 2932, 1655, 1618, 1556, 1446. ^{1}H-NMR ($CDCl_3$, 500 MHz): δH 7.60 (*d*, J=15.6 Hz, 1H), 7.50-7.56 (*m*, 2H), 7.34-7.31 (*m*, 3H), 6.39 (*d*, J=15.6 Hz, 1H), 3.94- 3.87 (*m*, 1H), 1.99 (*dd*, J=12.0 Hz, 4.0 Hz, 2H), 1.75- 1.59 (*m*, 3H), 1.43-1.34 (*m*, 2H), 1.25-1.16 (*m*, 3H). ^{13}C-NMR ($CDCl_3$. 125 MHz): δC 164.81, 138.34, 135.10, 129.60, 129.30, 128.83, 122.37, 48.50, 33.35, 25.67, 24.99 [21].

Phenylcinnamamide (**26**): **S**olid amorphous brown, Yield 54.9% (72.02 mg), M.p.: 150-154°C [22], TLC (9:1 hexane/EtOAc), Rf = 0.6 (7:3 Hex: AcOEt), IR vmax (KBr. cm-1): 3270, 3035, 1660, 1625, 1595, 1442. ^{1}H-NMR ($CDCl_3$. 400 MHz): δH 8.14 (*s*, 1H), 7.74 (*d*, J=16.0 Hz, 1H), 7.68-7.66 (*m*, 2H), 7.44-7.41 (*m*, 2H), 7.32-7.28 (*m*, 5H), 7.12 (*t*, J= 7.0 Hz, 1H), 6.66 (*d*, J=16.0 Hz, 1H). ^{13}C-NMR ($CDCl_3$. 100 MHz): δC 164.54, 142.37, 138.24, 134.71, 129.99, 129.01, 128.71, 127.77, 126.91, 121.16, 120.34 [22].

4-chlorophenylcinnamamide (**27**): Solid amorphous yellow, Yield 57.02% (78.8 mg), M.p.: 182-183°C [22], TLC (9:1 hexane/EtOAc), Rf = 0.6 (7:3 Hex:AcOEt), IR vmax (KBr. cm^{-1}): 3295, 3095, 1659, 1621, 1590, 1449 . ^{1}H-NMR (DMSO-d_6. 400 MHz): δH 9.48 (*t*, J=7.0 Hz, 1H), 6.88 (*d*, J=8.8 Hz, 2H), 6.77- 6.73 (*m*, 3H), 6.61- 6.52 (*m*, 5H), 5.96 (*d*, J=16.0 Hz, 1H). ^{13}C-NMR (DMSO-d_6. 100 MHz): δC 163.02, 140.51, 138.22, 134.61, 129.85, 129.01, 128.71, 127.11, 126.91, 121.94, 120.76 [23].

Benzylcinnamamide (**28**): Yellow solid; Yield 80.5% (110 mg); m.p.: 109–111°C [24]; $C_{16}H_{15}NO$; 237.30 g/mol; TLC (7:3 hexane/EtOAc); Rf = 0.42; IR υmax (KBr, cm^{-1}): 3261, 3029, 2926, 1653, 1616, 1497; ^{1}H-NMR ($CDCl_3$, 500 MHz): δH 7.75 (1H; *d*; J = 16.0 Hz), 7.54–7.50 (2H; *m*), 7.44–7.34 (8H; *m*), 6.50 (*d*, 1H, J = 16.0 Hz), 5.26 (2H; *s*), ^{13}C-NMR ($CDCl_3$, 125 MHz,): δC 166.03, 145.04, 133.95, 132.74, 130.64, 129.97, 128.94, 128.49, 128.44, 117.72, 66.83 [25,26].

4-Chlorobenzylcinnamamide (**29**): White solid; Yield 58.0% (94.7 mg); m.p.: 127–129°C [27]; $C_{15}H_{12}ClNO$; 283.28 g/mol; TLC (7:3 hexane/EtOAc); R_f= 0.7; IR υmax (KBr, cm^{-1}): 3259, 3083, 2921, 1655, 1621; 1489; ^{1}H-NMR ($CDCl_3$, 500 MHz): 7.48 (*d*, H-7, J=15.5 Hz, H=1), 7.31-7.29 (*m*, H=2), 7.19-7.17 (*m*, H=3), 7.13-7.11 (*m*, H=2), 7.09-7.06 (*m*, H=2), 6.26 (*d*, J=15,5 Hz, H=1), 5.95 (*s*, H=1), 4.35 (*d*, J=6.0 Hz, H=2); ^{13}C-NMR ($CDCl_3$, 125 MHz): δC 166.02, 141.84 , 136.92, 134.79, 133.49, 129.96, 129.33, 128.98, 128.98, 127.95, 120.30, 43.23 [10,28].

4- methoxybenzylcinnamamide (**31**): Solid amorphous white, Yield 62.01% (83.3 mg), M.p.: 192-194° C [23], TLC (9:1 hexane/EtOAc), Rf = (7:3 Hex:AcOEt), IR vmax (KBr. cm^{-1}): 3271, 3082, 2961, 1655, 1618, 1560, 1469. ^{1}H-NMR (DMSO-d6, 400 MHz): δH 7.71 (*d*, J= 16.0 Hz, 1H), 7.53-7.49 (*m*, 2H), 7.39-7.37 (*m*, 3H), 6.96-6.89 (*m*, 4H), 6.47 (*d*, J= 16.0 Hz, 1H), 5.16 (*s*, 2H), 3.42 (*s*, 3H). ^{13}C-NMR (DMSO-d6, 100 MHz): δC 166.92 , 145.86, 145.12, 134.38, 130.35, 128.90, 128.10, 127.92, 122.15, 118.00, 114.38, 66.72, 55.96 [23].

4-Isopropylbenzylcinnamamide (**32**): Yellow oil; Yield 57.3%; $C_{19}H_{21}NO$; 239.38 g/mol; TLC (7:3 hexane/EtOAc); R_f = 0.53; IR υmax (KBr, cm^{-1}): 3283, 3092, 2921, 2839, 1654, 1621, 1489. ^{1}H-NMR ($CDCl_3$, 500 MHz): δH 7.75 (*d*, J=15.0 Hz, 1H), 7.37–7.33 (*m*, 2H), 7.29–7.27(*m*, 7H), 6.65 (*d*,J=15.0 Hz, 1H), 4.97 (*sept*, J= 6.5 Hz, 1H), 4.67-4.61(*m*, 2H), 1.16 (*d*, J=7.0 Hz, 6H); ^{13}C-NMR ($CDCl_3$, 125 MHz,): 167.27 (C=O), 142.99 (C-7), 139.03 (C-4′), 135.50 (C-1, C-1′), 129.57 (C-3, C-5), 128.80 (C-2, C-6), 127.90 (C-4, C-2′, C-6′), 126.15 (C3′, C-5′), 119.07 (C-8), 46.33 (C-7′), 46.16 (C-8′), 20.45 (C-9′) [25,27].

4-methylbenzylcinnamamide (**33**): solid amorphous yellow, Yield 67.3%, TLC (9:1 hexane/EtOAc), Rf = 0.32 (7:3 Hex: AcOEt), IV υmax (KBr. cm^{-1}): 3259, 3083, 2921, 1655, 1621, 1567, 1422; ^{1}H-NMR (400 MHz. $CDCl_3$): 7.66 (*d*, *J*= 16.0 Hz, 1H), 7.29 (*dd*, J=8.5 Hz, 16.0 Hz, 2H), 7.36-7.33 (*m*, 3H), 7.29 (*d*, *J*= 6.8 Hz, 1H), 7.24 (*d*, *J*= 6.8 Hz, 1H), 6.43 (*d*, J=16.0 Hz, 1H), 4.52 (*d*, J=5.5 Hz, 2H), 1.73 (*s*, 3H). ^{13}C-NMR (100 MHz. $CDCl_3$): 165.90, 141.73, 136.80, 134.67, 133.37, 129.84, 129.21, 128.86, 127.86, 120.18, 43.10, 24.59.

Piperonylcinnamamide (**34**): White oil; Yield 74.7%; $C_{17}H_{15}NO_3$; 281.31 g/mol; TLC (7:3 hexane/EtOAc); R_f = 0.57; IR υmax (KBr, cm^{-1}): 3183, 2982, 2821, 1655, 1621, 1489; ^{1}H-NMR (DMSO-d_6, 400 MHz): δH 8.48 (*t*, J=7.5 Hz, H=1); 7.48-7.45 (*m*, 2H), 7.38 (*d*, J= 16.0 Hz, 1H), 7.34- 7.25 (*m*, 3H), 6.77- 6.75 (*m*, 2H), 6.68 (*dd*, J= 8.0 Hz; 2.0 Hz, 1H), 6.59 (*d*, J=16.0 Hz, 1H), 5.88 (*s*, 2H), 4.21 (*d*, J= 6.0 Hz, 2H). ^{13}C-NMR (DMSO-d_6, 100 MHz): δC 164.94, 147.30, 146.15, 139.00, 134.92, 133.29, 129.51, 129.40, 127.57, 122.52, 121.09, 108.11, 100.87, 42.18 [29].

Dibenzylcinnamamide (**35**): White solid; Yield 81.6% (121.2 mg); m.p.: 129-130°C [30]. $C_{23}H_{25}NO_3$; 363.45 g/mol; TLC (7:3 hexane/EtOAc); R_f= 0.48; IR υmax (KBr, cm^{-1}): 3084, 2982, 1643, 1593, 1495. ^{1}H-NMR ($CDCl_3$, 400 MHz): δH 7.85 (*d*, J=15.2 Hz, 1H), 7.45-7.42 (*m*, 2H), 7.36-7.27 (*m*, 11H), 7.22-7.20 (*m*, 2H), 6.90 (*d*, H-8, J= 15.2 Hz, 1H), 4.70 (*s*, 2H), 4.59 (*s*, 2H). ^{13}C-NMR ($CDCl_3$, 400 MHz): δC 167.36, 143.97, 137.46, 136.78, 135.31, 129.97, 129.81, 129.11, 128.74, 128.50, 129.81, 129.11, 128.74, 127.99, 127.84, 126.71, 127.56, 117.39, 50.20, 48.95 [29,30].

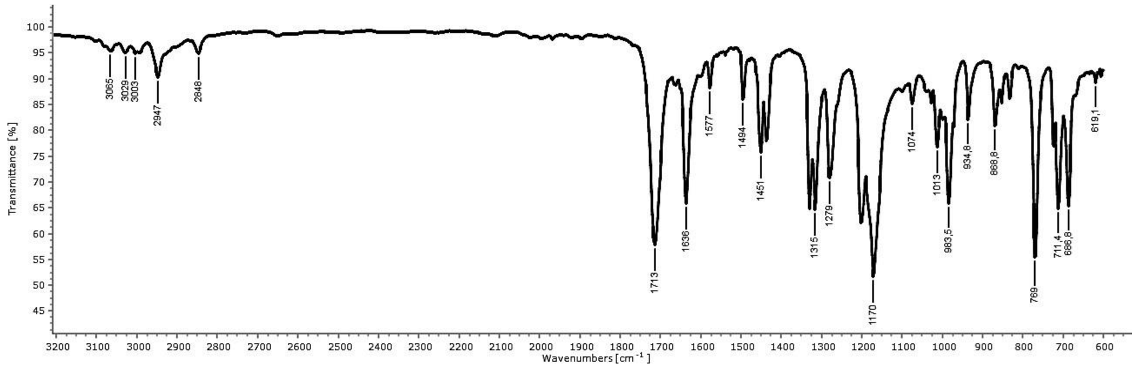

Figure S1. IR υmax (KBr, cm^{-1}) spectrum of Methyl cinnamate (**2**)

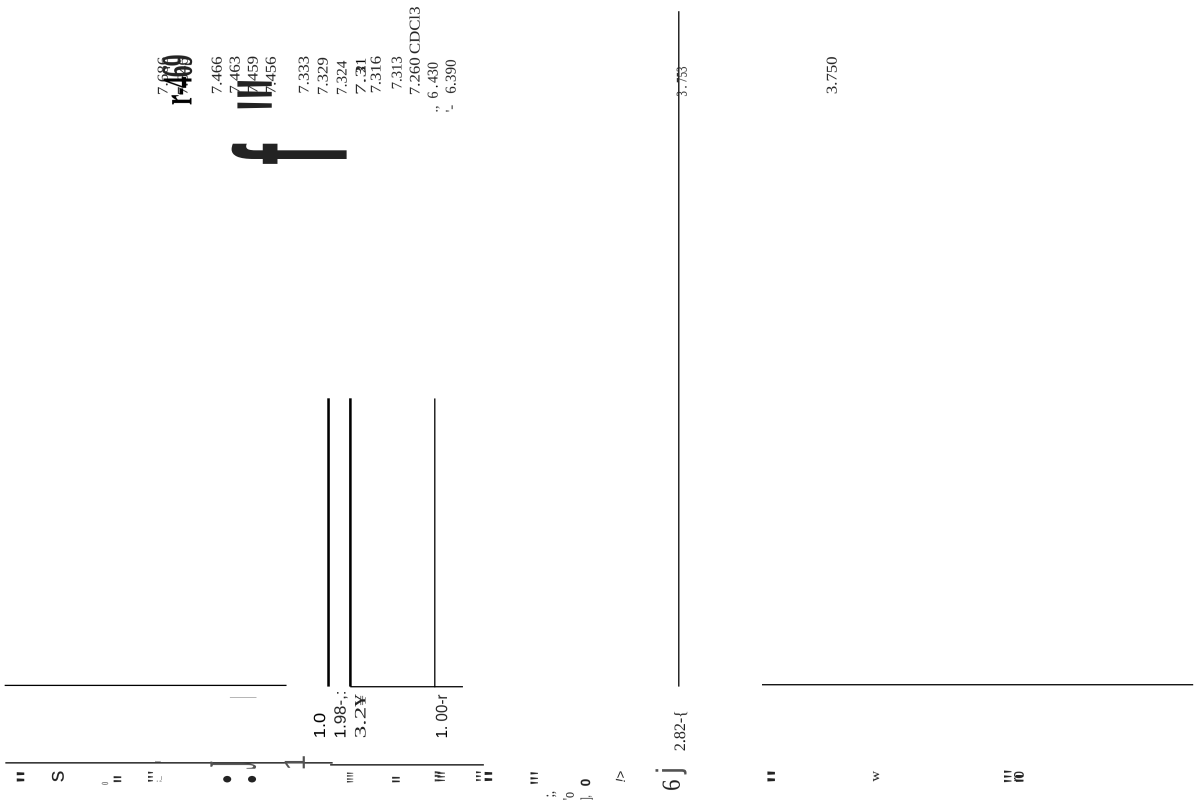

Figure S2. 1H NMR (400MHz, $CDCl_3$) spectrum of Methyl cinnamate (**2**)

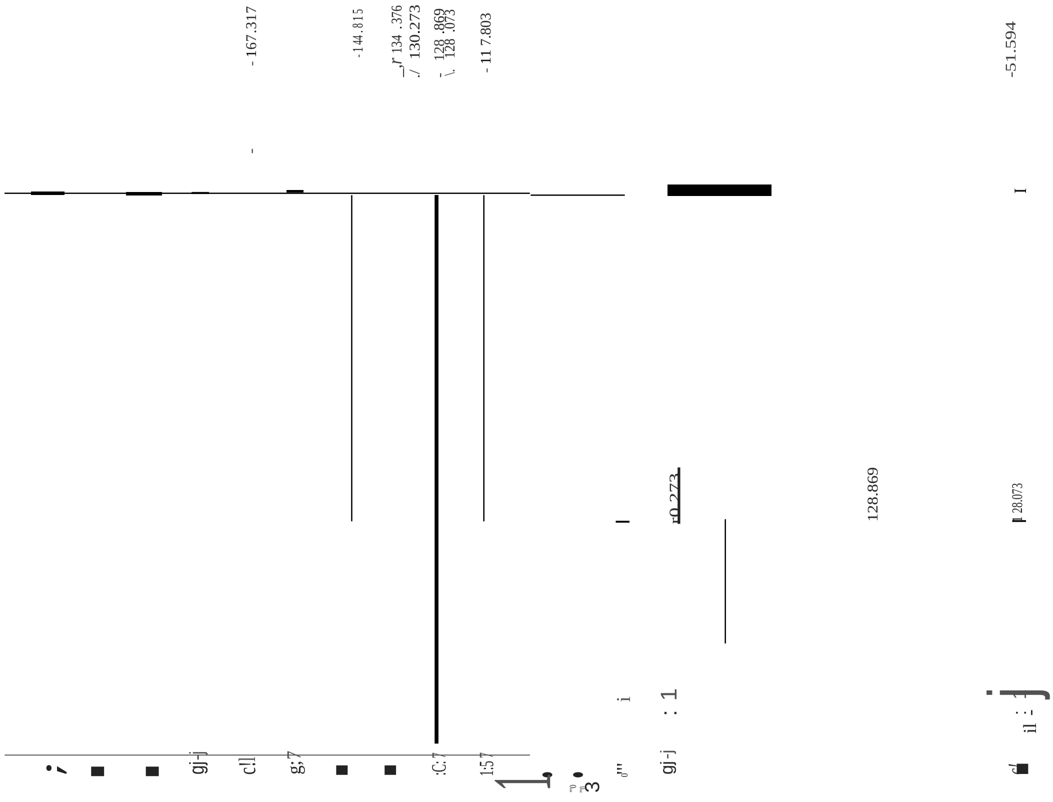

Figure S3. ^{13}C NMR (100MHz, $CDCl_3$) spectrum of of methyl cinnamate (**2**)

g;

ow

;;NJ

N

2-

:C:i
1:5 1 ec J

,..
"

------ l117.803 '''

II

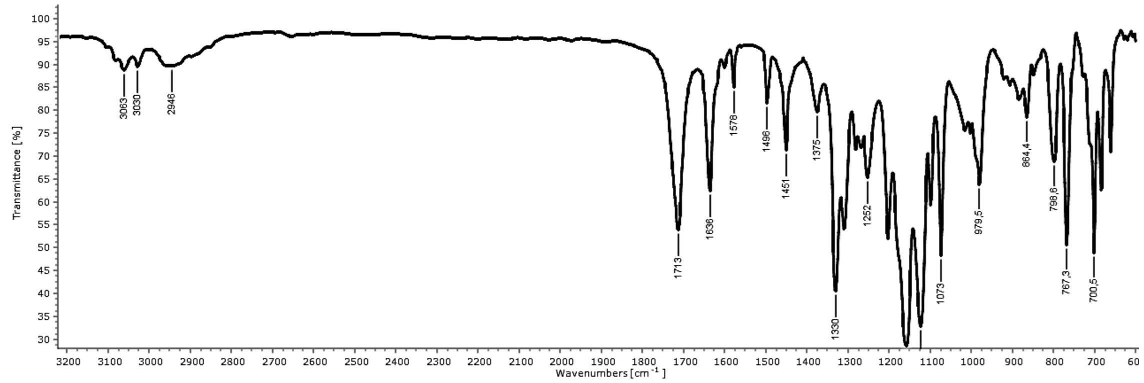

Figure S4. IR υmax (KBr, cm⁻¹) spectrum of ethyl cinnamate (**3**)

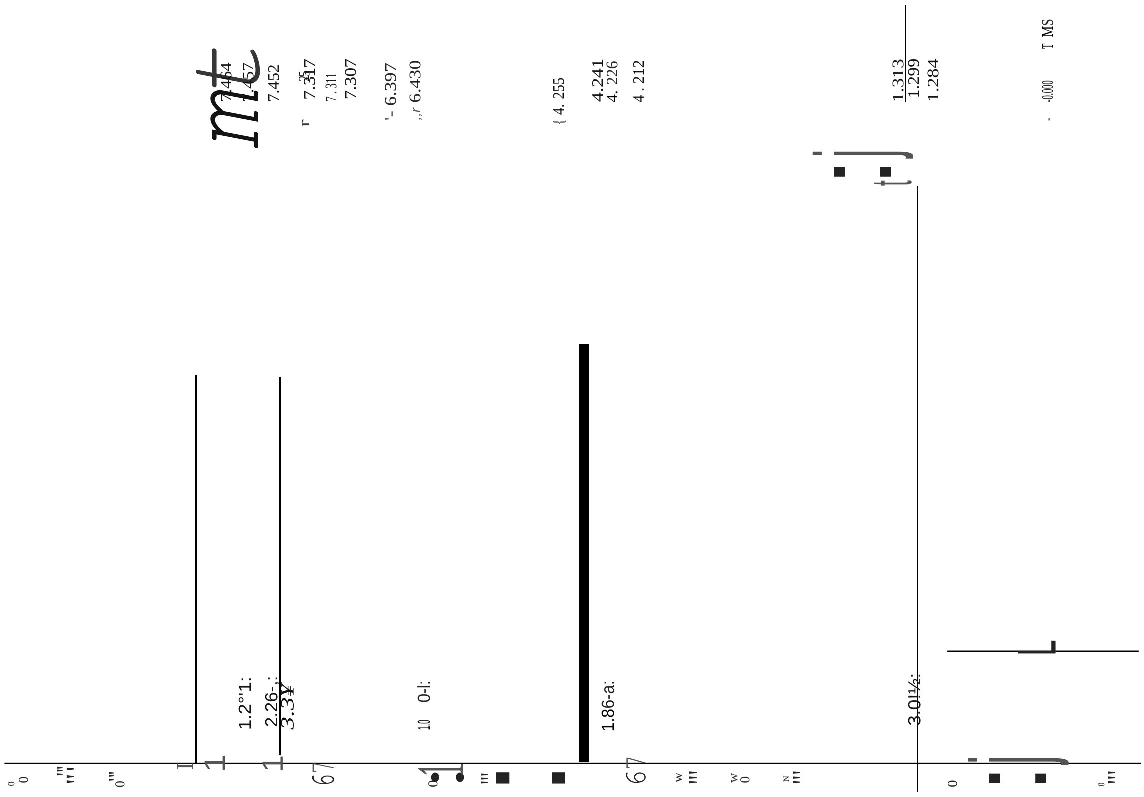

Figure S5. ^{1}H NMR (500MHz, $CDCl_3$) spectrum of ethyl cinnamate (**3**)

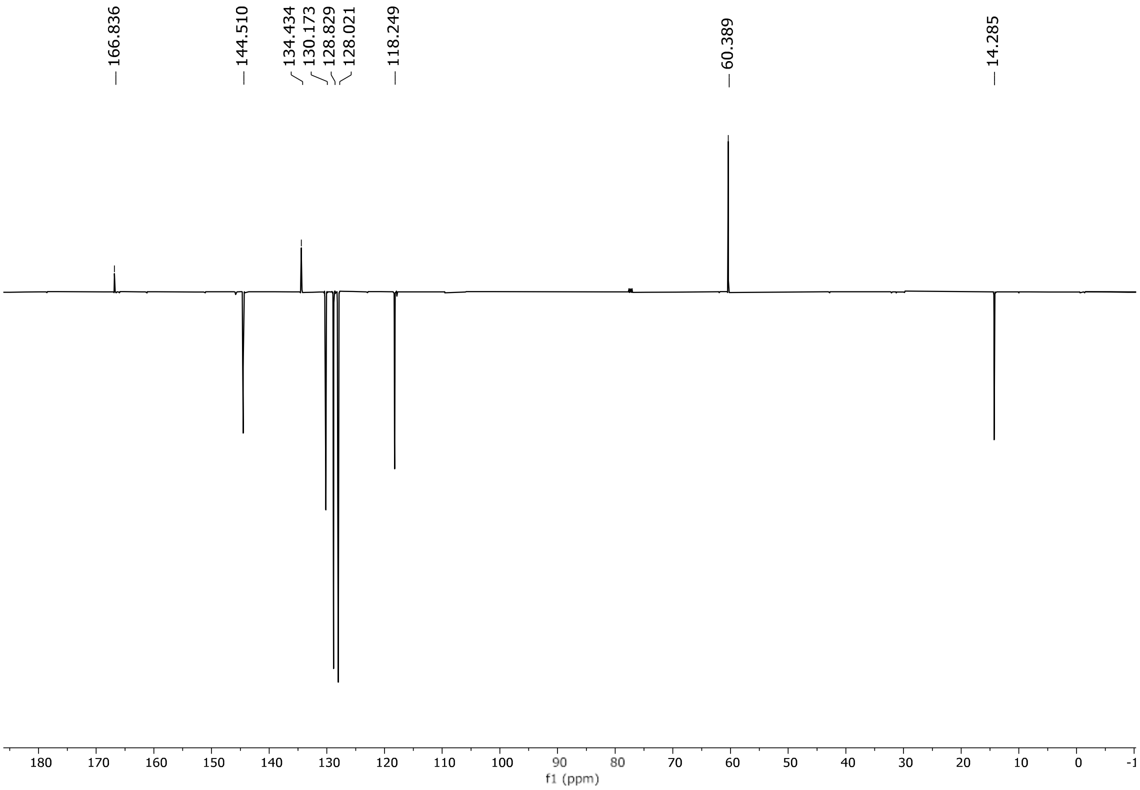

Figure S6. ^{13}C NMR (125 MHz, $CDCl_3$) spectrum of of ethyl cinnamate (**3**)

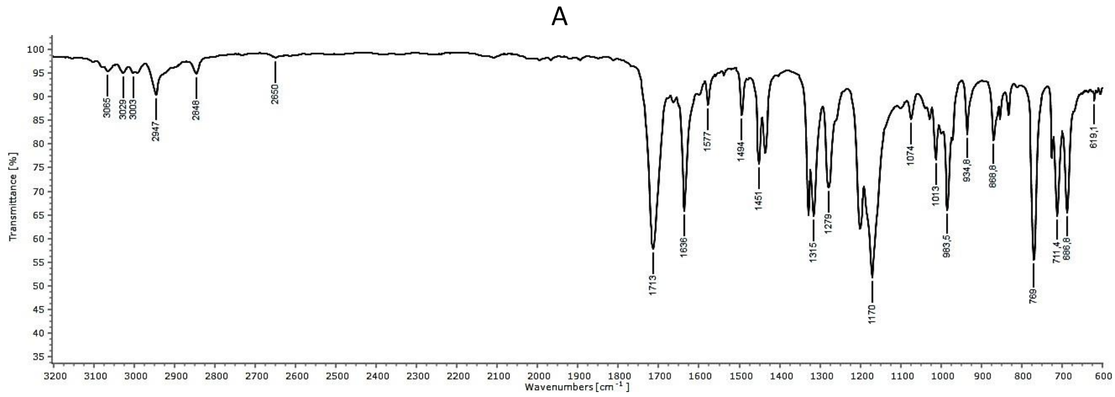

Figure S7. IR υmax (KBr, cm^{-1}) spectrum of propyl cinnamate (**4**)

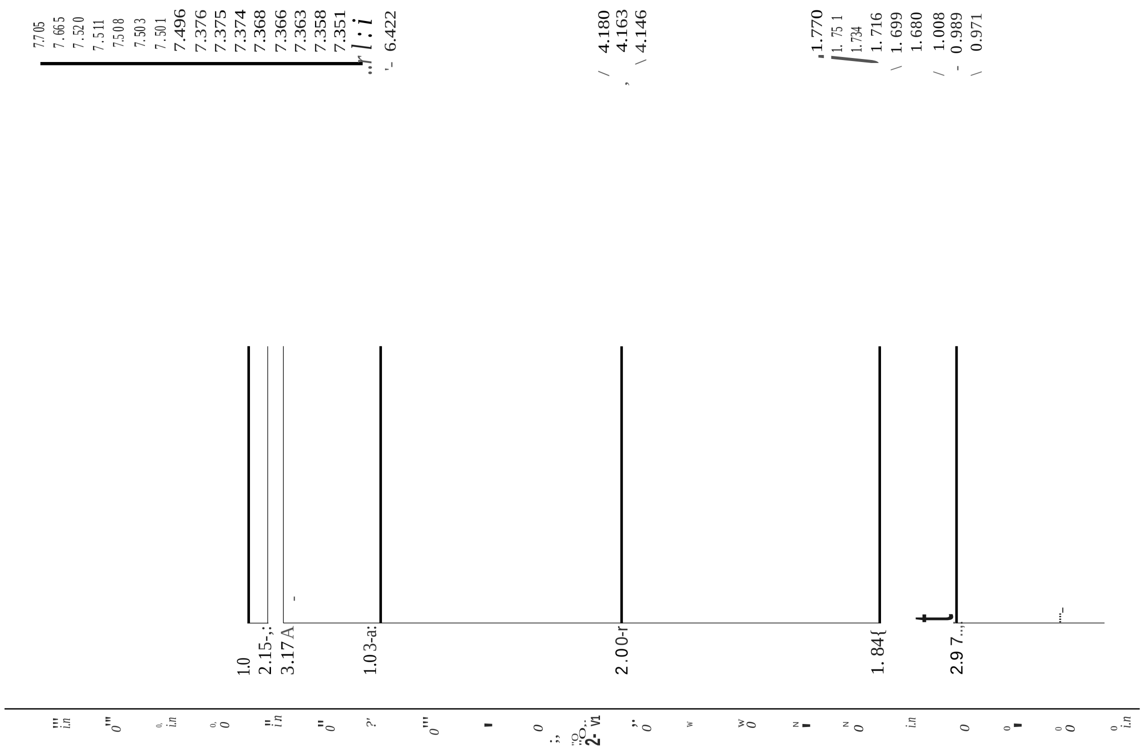

Figure S8. ^{1}H NMR (500 MHz, $CDCl_3$) spectrum of propyl cinnamate (**4**)

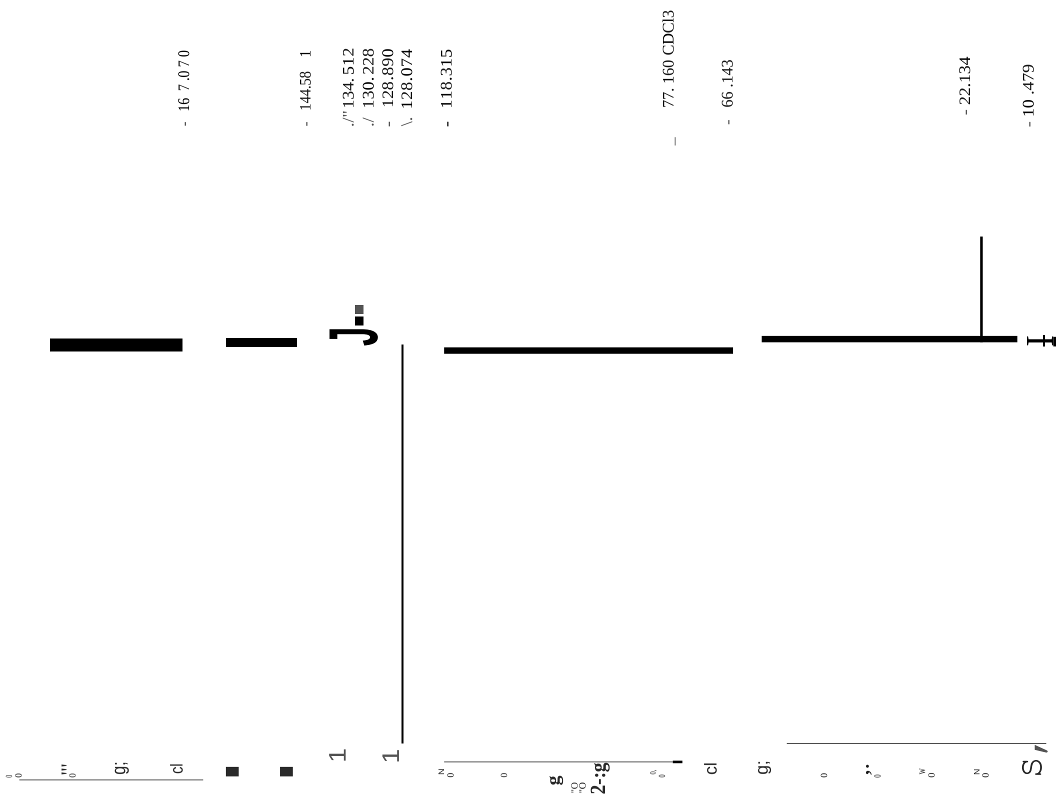

Figure S9. ^{13}C NMR (125 MHz, $CDCl_3$) spectrum of of propyl cinnamate (**4**)

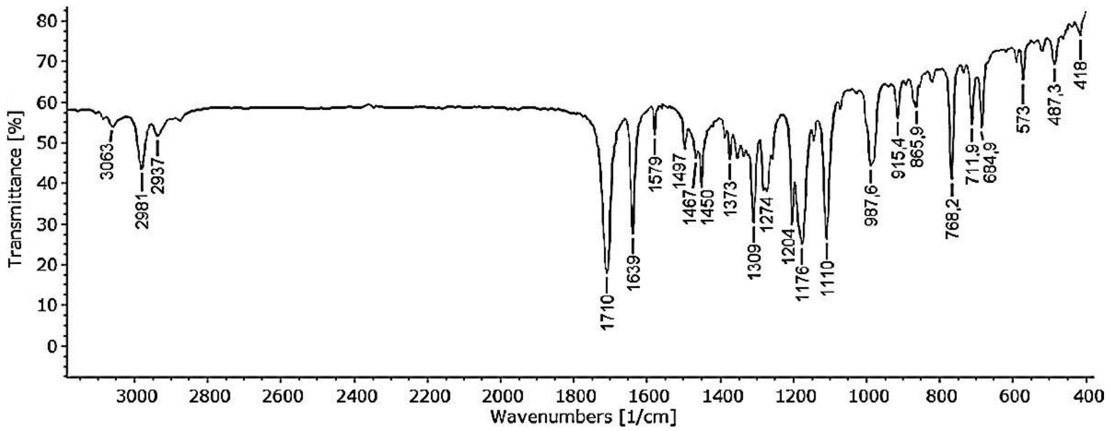

Figure S10. IR υmax (KBr, cm^{-1}) spectrum of isopropyl cinnamate (**5**)

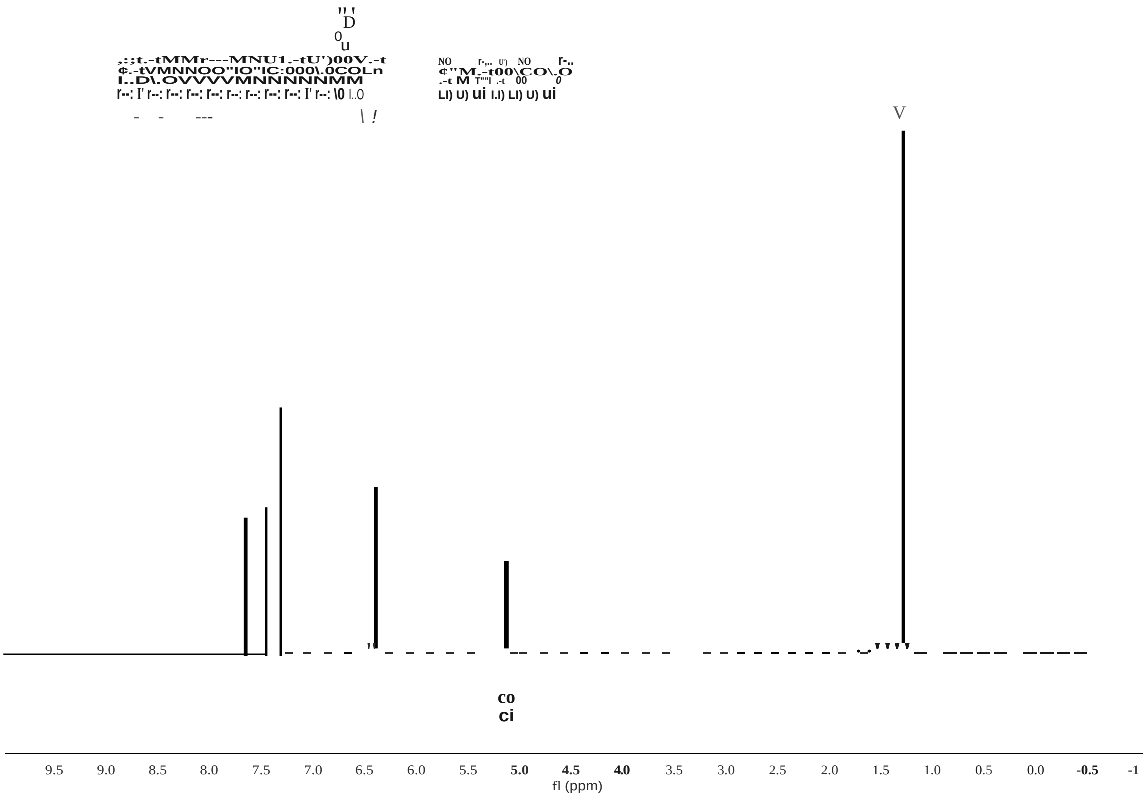

Figure S11. ^{1}H NMR (500 MHz, $CDCl_3$) spectrum of isopropyl cinnamate (**5**)

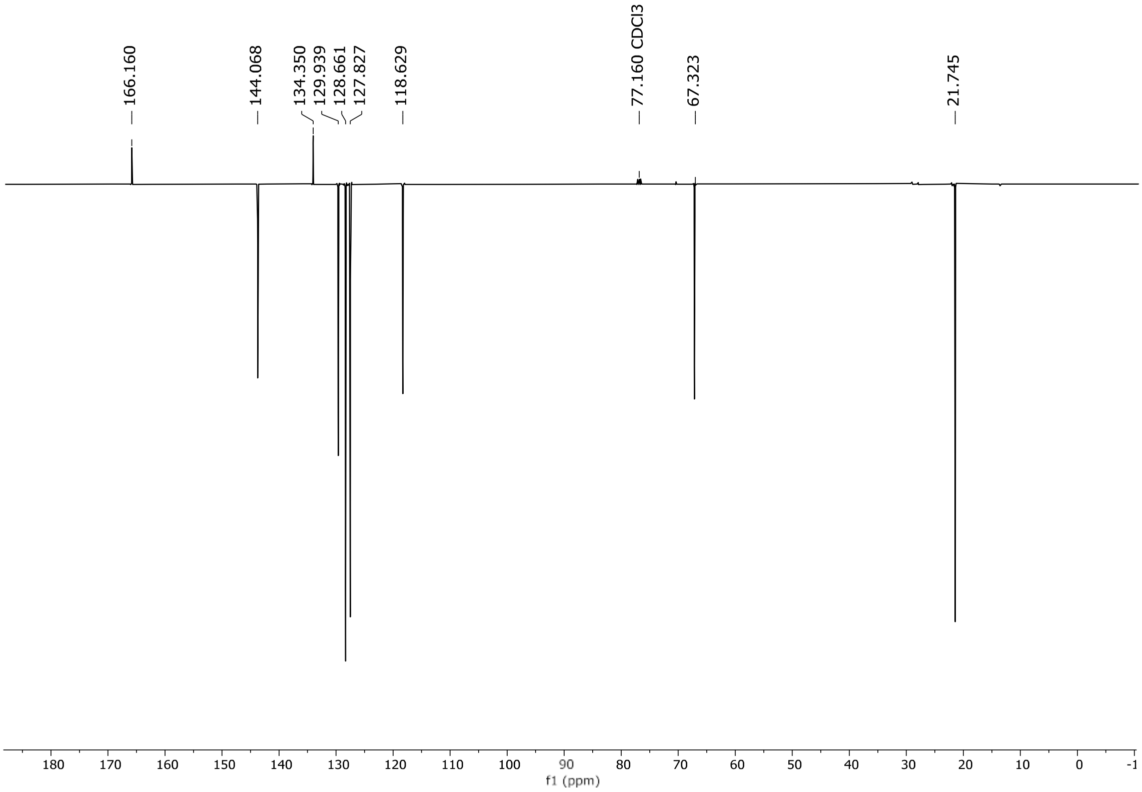

Figure S12. ^{13}C NMR (125 MHz, $CDCl_3$) spectrum of of isopropyl cinnamate (**5**)

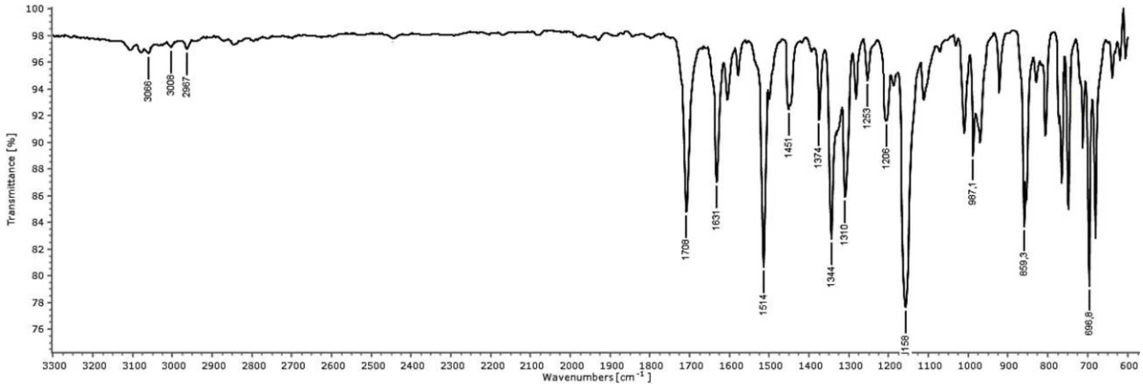

Figure S13. IR ʋmax (KBr, cm^{-1}) spectrum of butyl cinnamate (**6**)

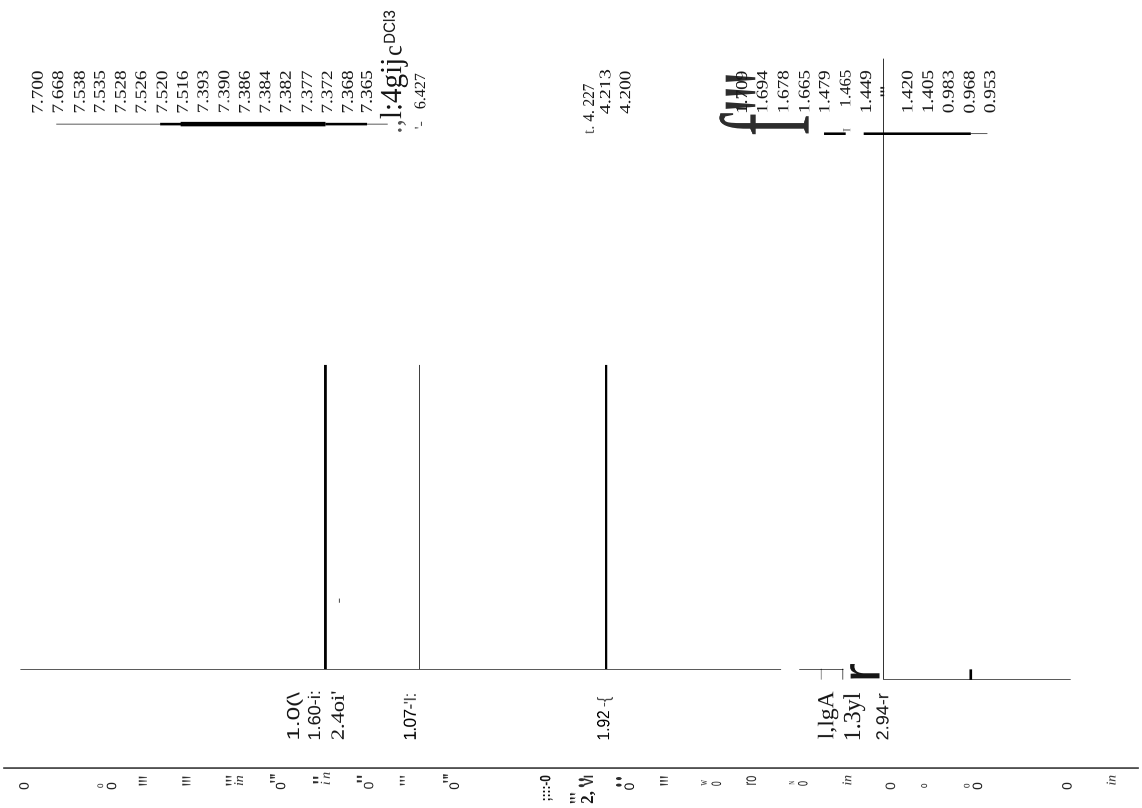

Figure S14. ^{1}H NMR (400MHz, $CDCl_3$) spectrum of butyl cinnamate (**6**)

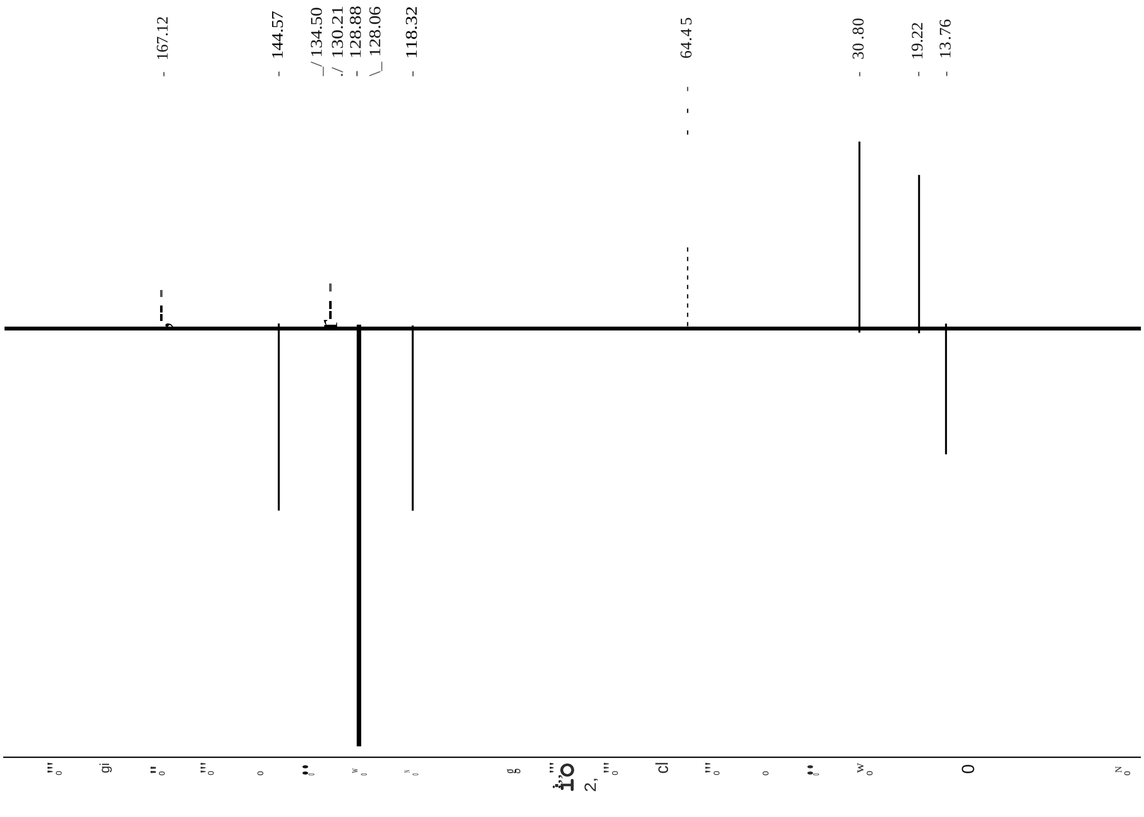

Figure S15. ^{13}C NMR (100MHz, $CDCl_3$) spectrum of butyl cinnamate (**6**)

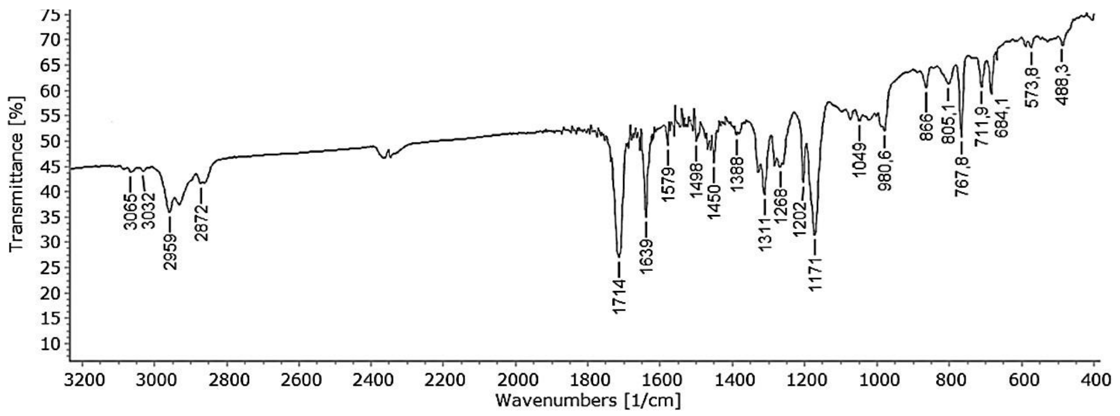

Figure S16. IR υmax (KBr, cm⁻¹) spectrum of pentyl cinnamate (**7**)

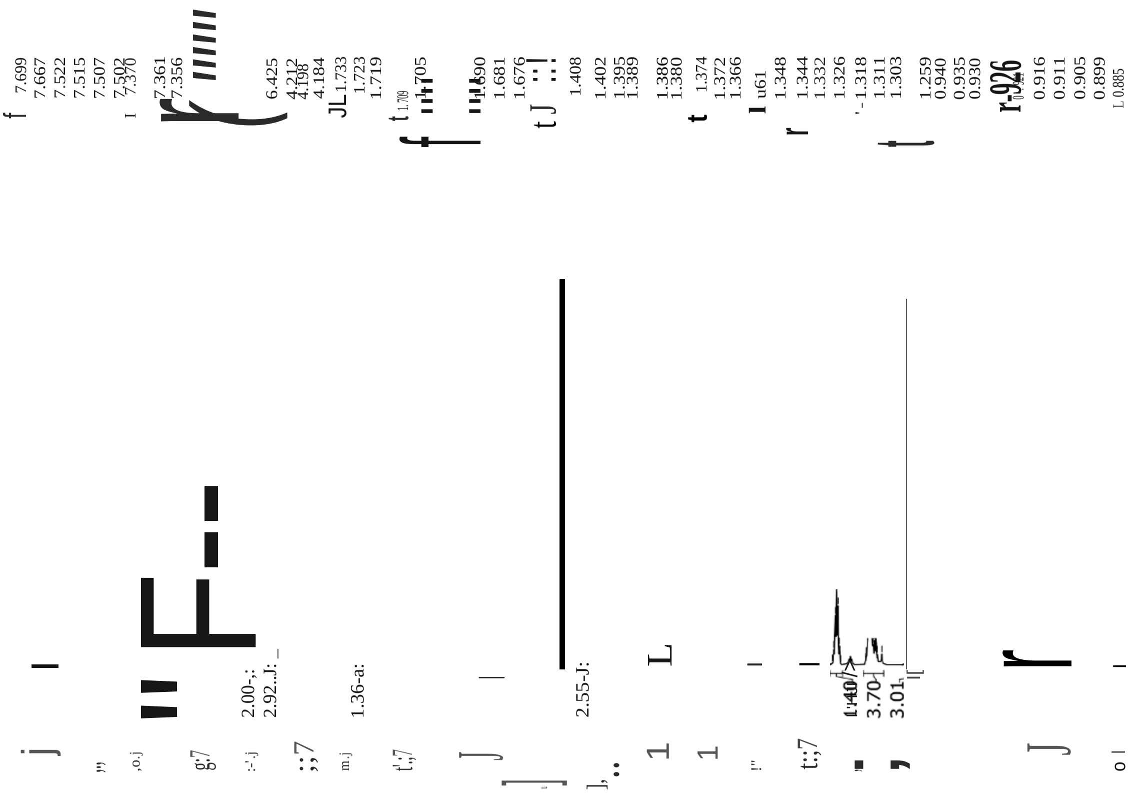

Figure S17. ^{1}H NMR (400MHz, $CDCl_3$) spectrum of pentyl cinnamate (**7**)

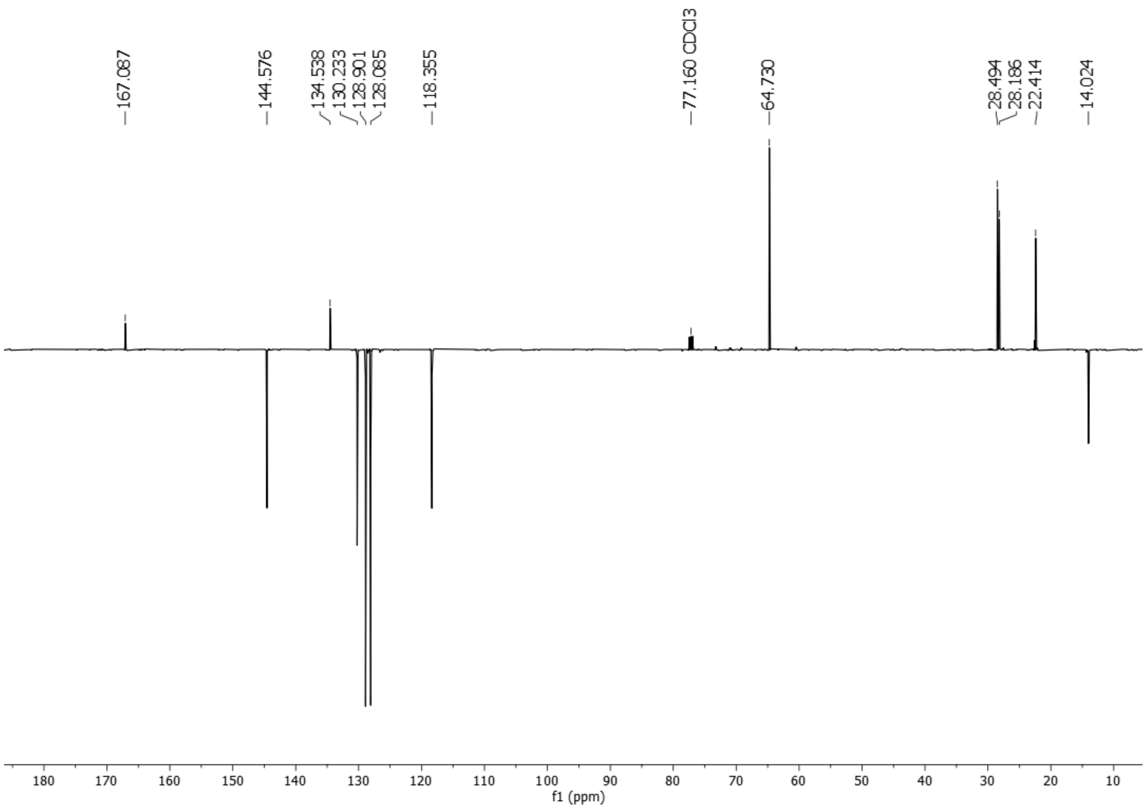

Figure S18. ^{13}C NMR (100MHz, $CDCl_3$) spectrum of pentyl cinnamate (**7**)

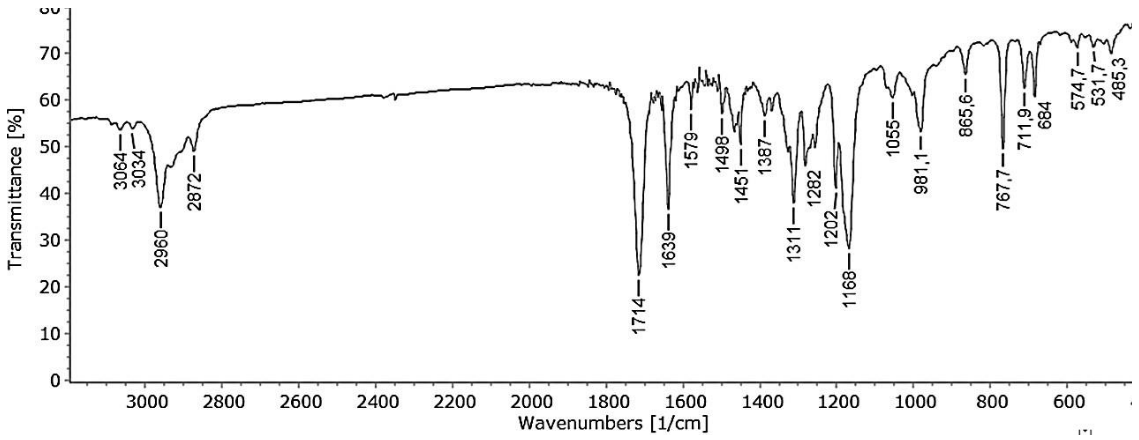

Figure S19. IR υmax (KBr, cm^{-1}) spectrum of isopentyl cinnamate (**8**)

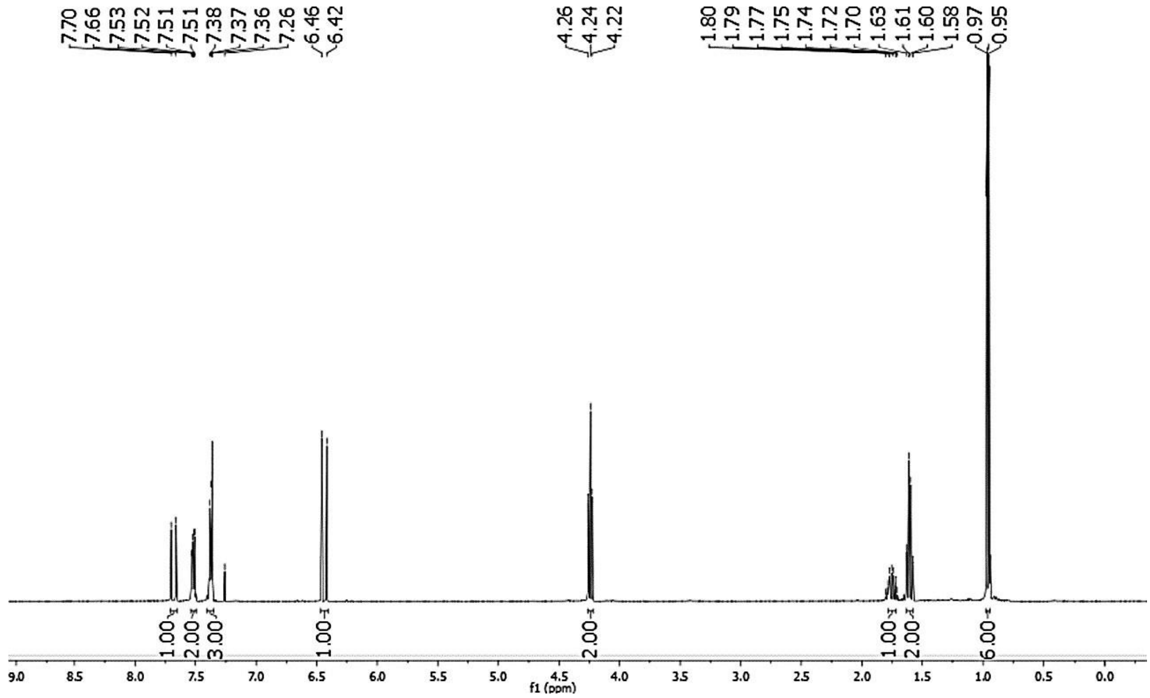

Figure S20. ^{1}H NMR (400MHz, $CDCl_3$) spectrum of isopentyl cinnamate (**8**)

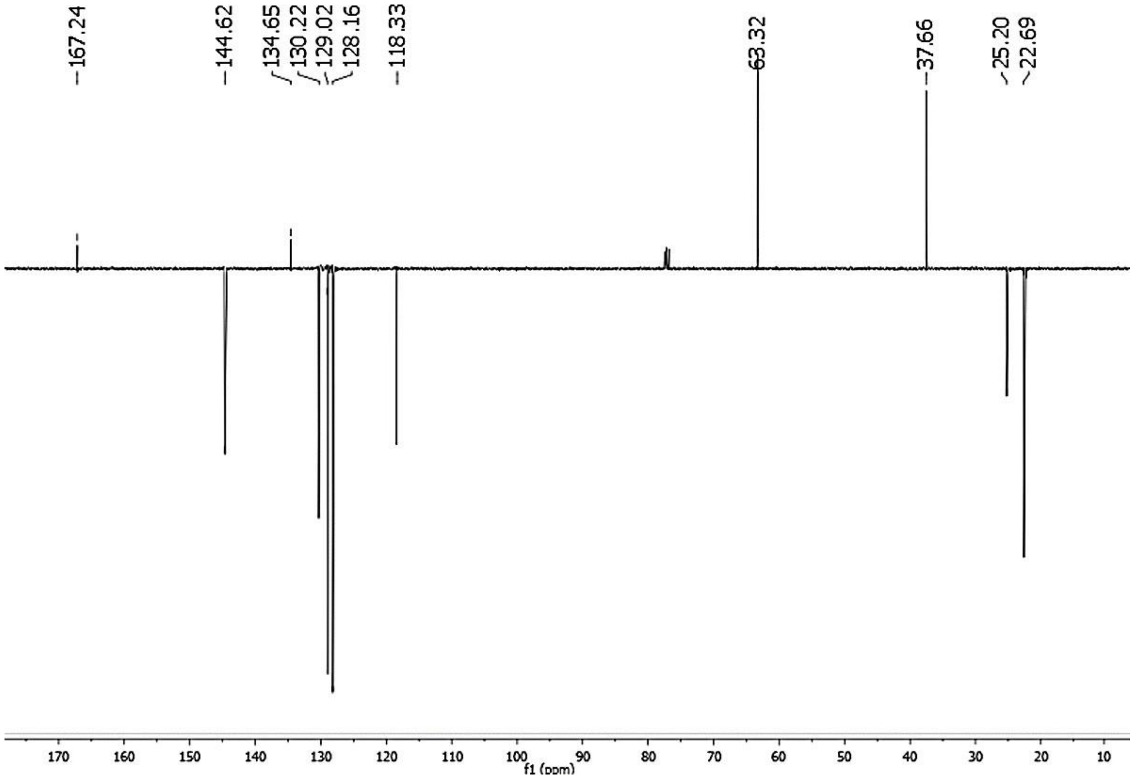

Figure S21. ^{13}C NMR (100MHz, $CDCl_3$) spectrum of isopentyl cinnamate (**8**)

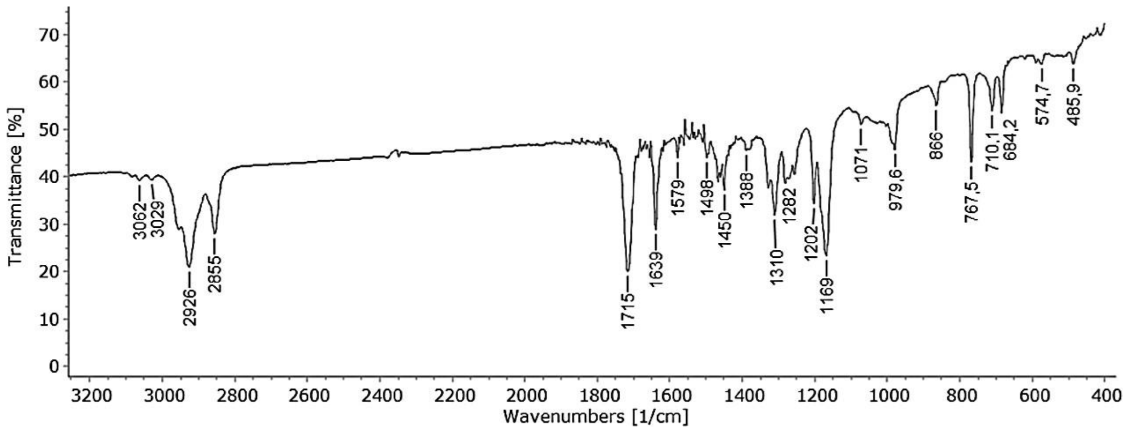

Figure S22. IR υmax (KBr, cm^{-1}) spectrum of decyl cinnamate (**9**)

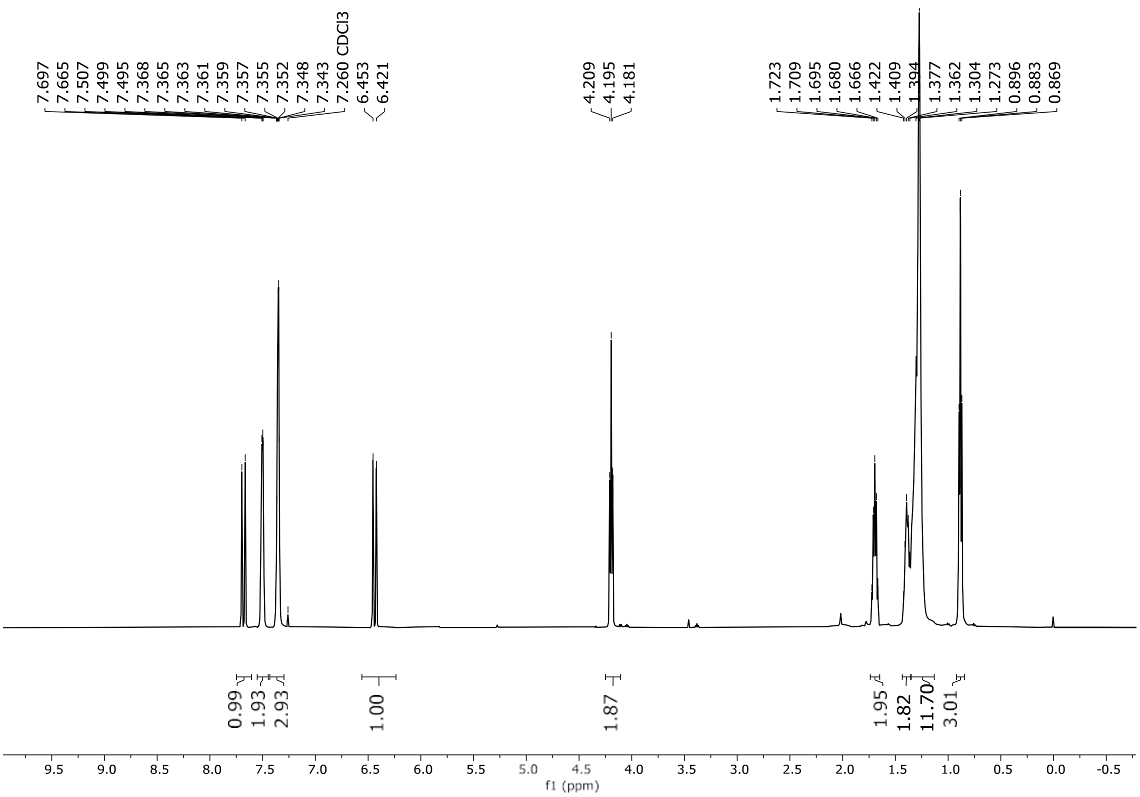

Figure S23. ^1H NMR (500 MHz, $CDCl_3$) spectrum of decyl cinnamate (**9**)

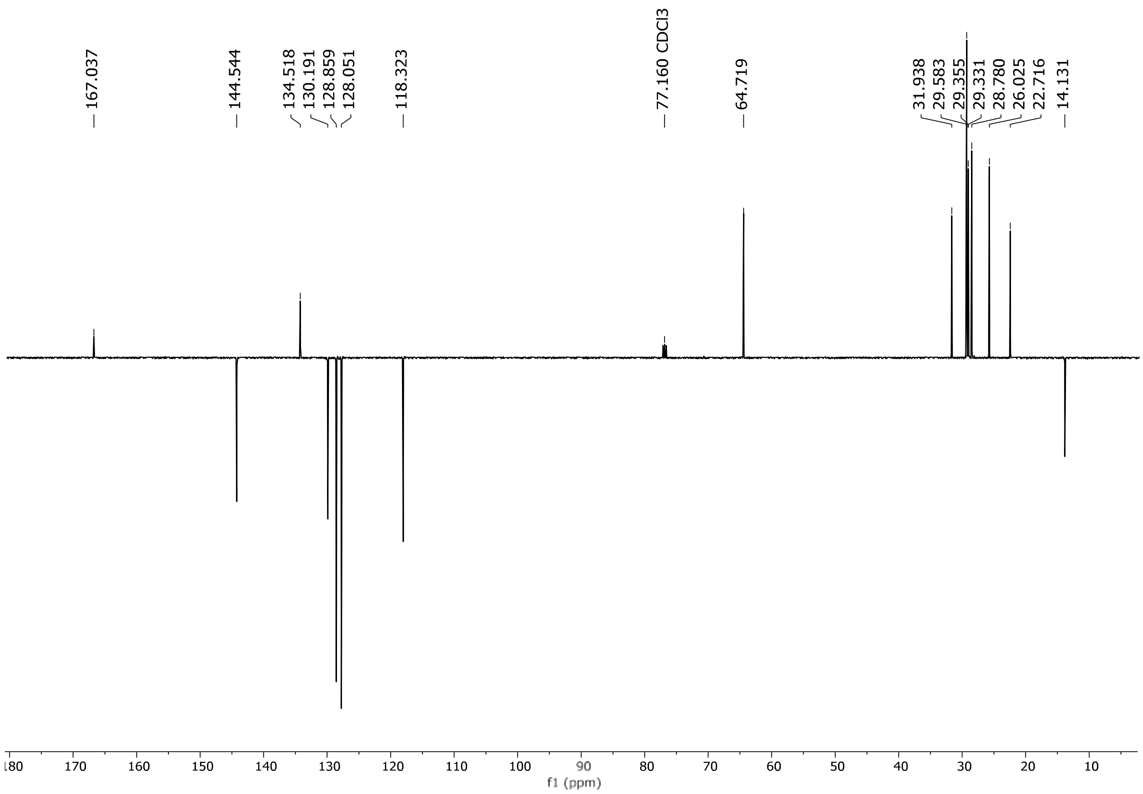

Figure S24. ^{13}C NMR (125 MHz, $CDCl_3$) spectrum of decyl cinnamate (**9**)

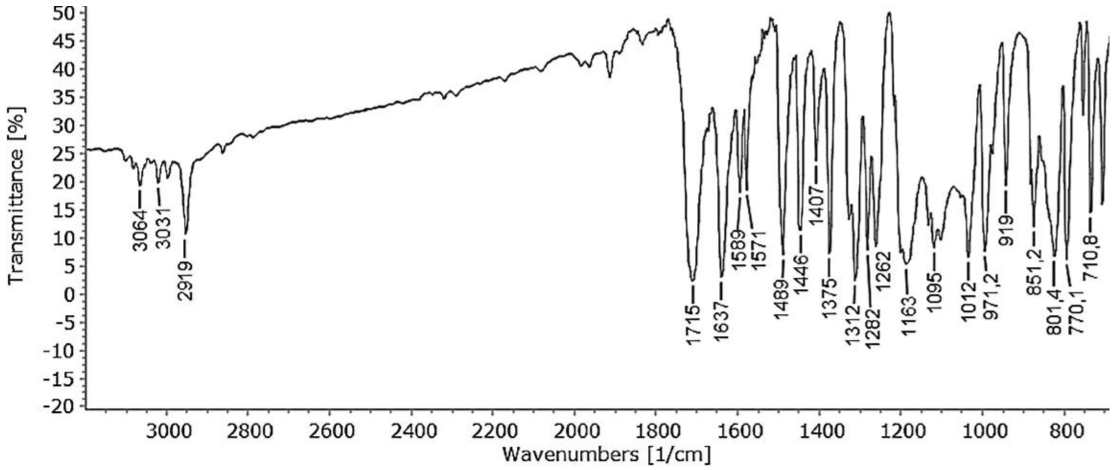

Figure S25. IR υmax (KBr, cm^{-1}) spectrum of benzyl cinnamate (**10**)

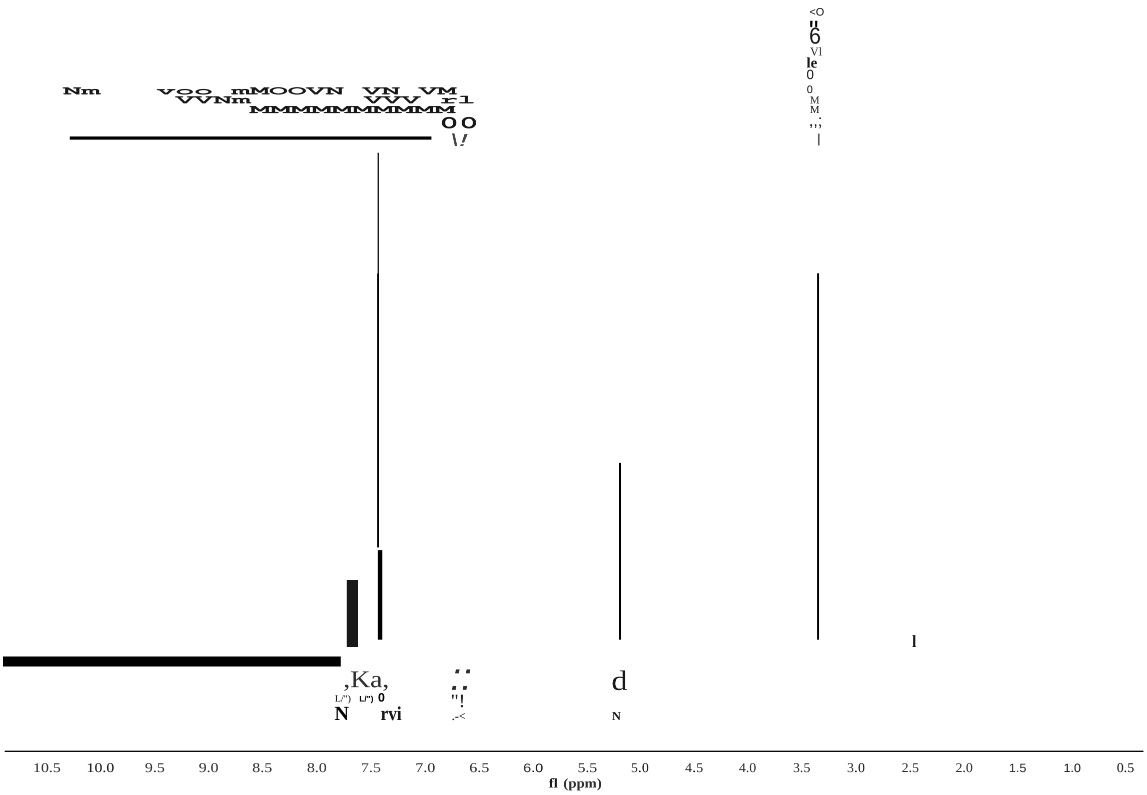

Figure S26. ^{1}H NMR (400MHz, $CDCl_3$) spectrum of benzyl cinnamate (**10**)

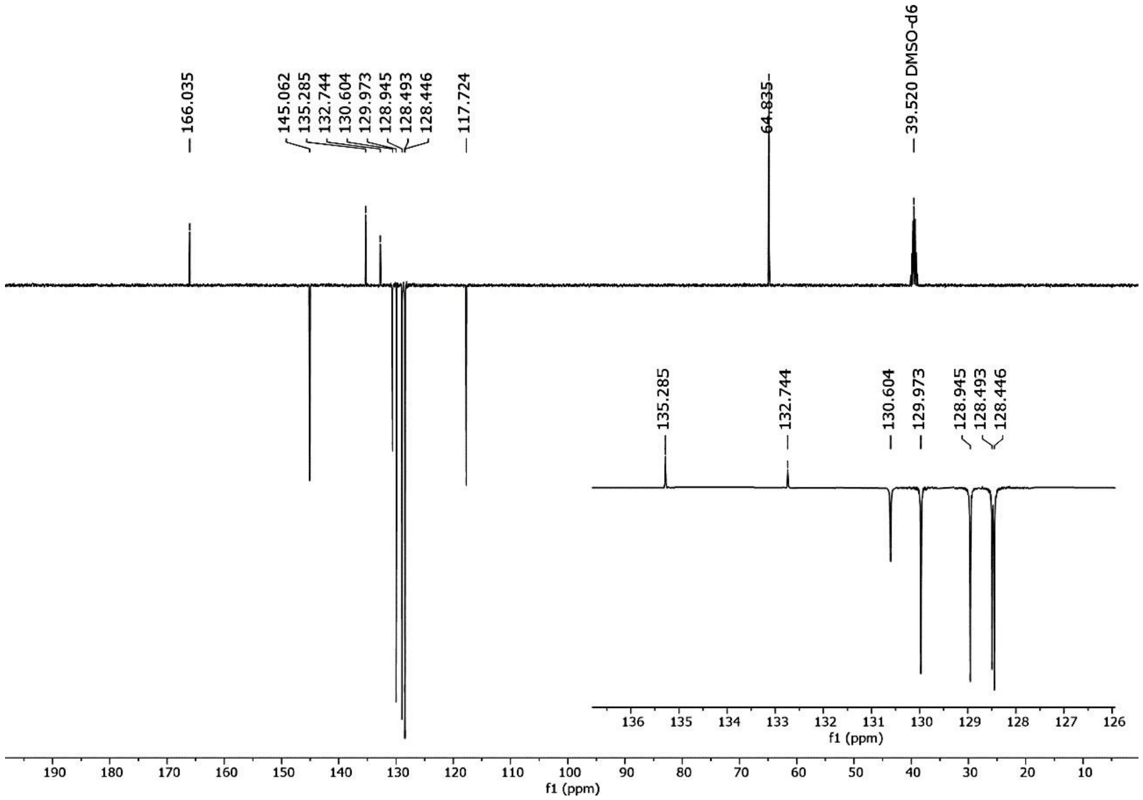

Figure S27. ^{13}C NMR (100 MHz, $CDCl_3$) spectrum of benzyl cinnamate (**10**)

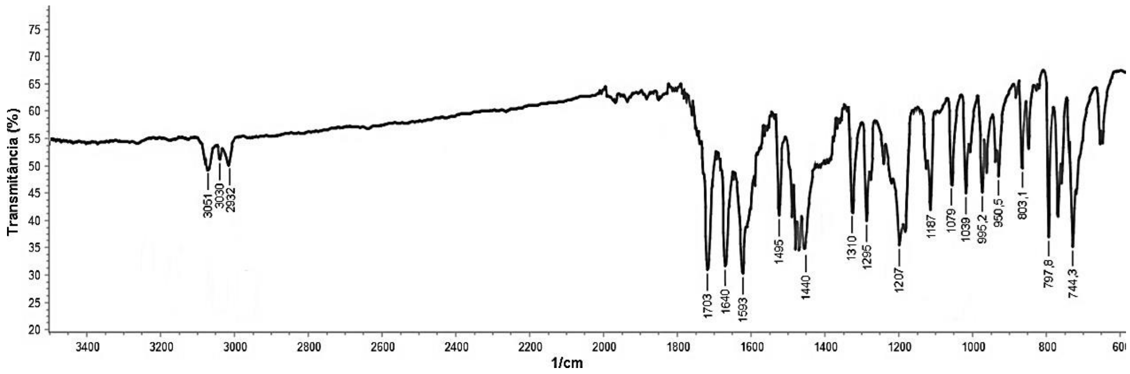

Figure S28. IR υmax (KBr, cm^{-1}) spectrum of 4-methylbenzyl cinnamate (**11**)

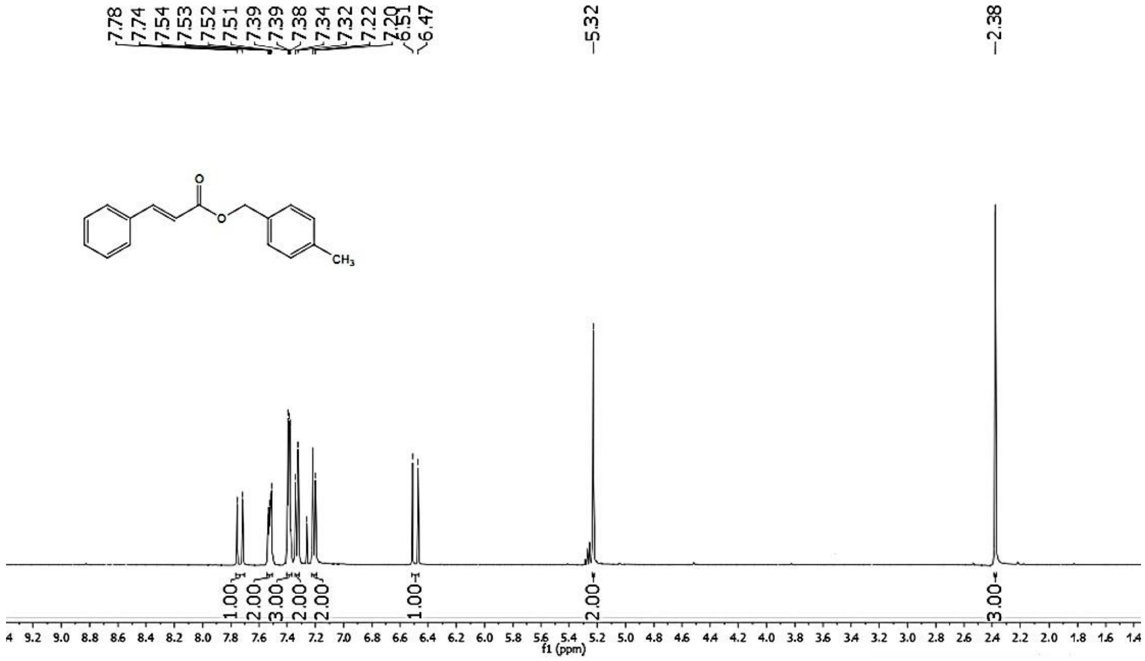

Figure S29. ^{1}H NMR (400MHz, $CDCl_3$) spectrum of 4-methylbenzyl cinnamate (**11**)

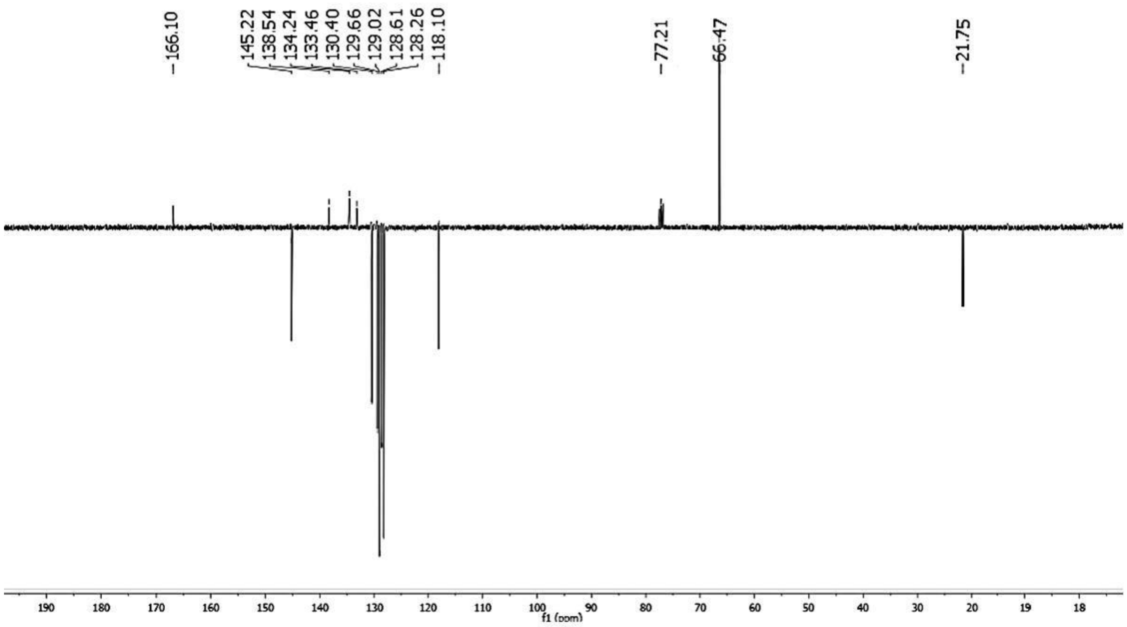

Figure S30. ^{13}C NMR (100MHz, $CDCl_3$) spectrum of 4-methylbenzyl cinnamate (**11**)

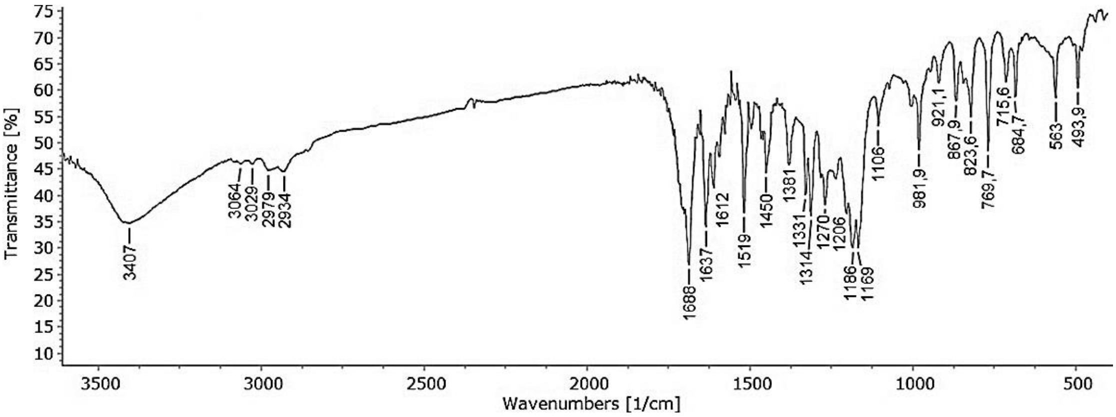

Figure S31. IR υmax (KBr, cm^{-1}) spectrum of 4-hydroxybenzyl cinnamate (**12**)

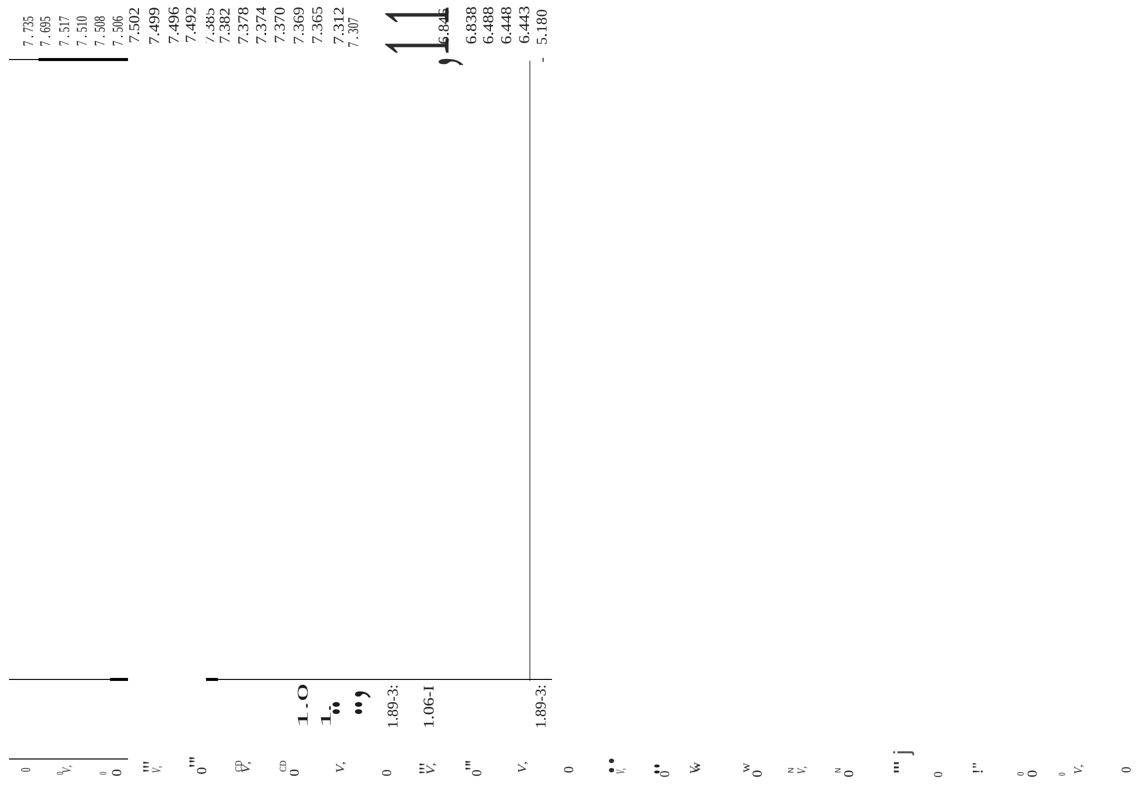

Figure S32. ^{1}H NMR (400MHz, $CDCl_3$) spectrum of 4-hydroxybenzyl cinnamate (**12**)

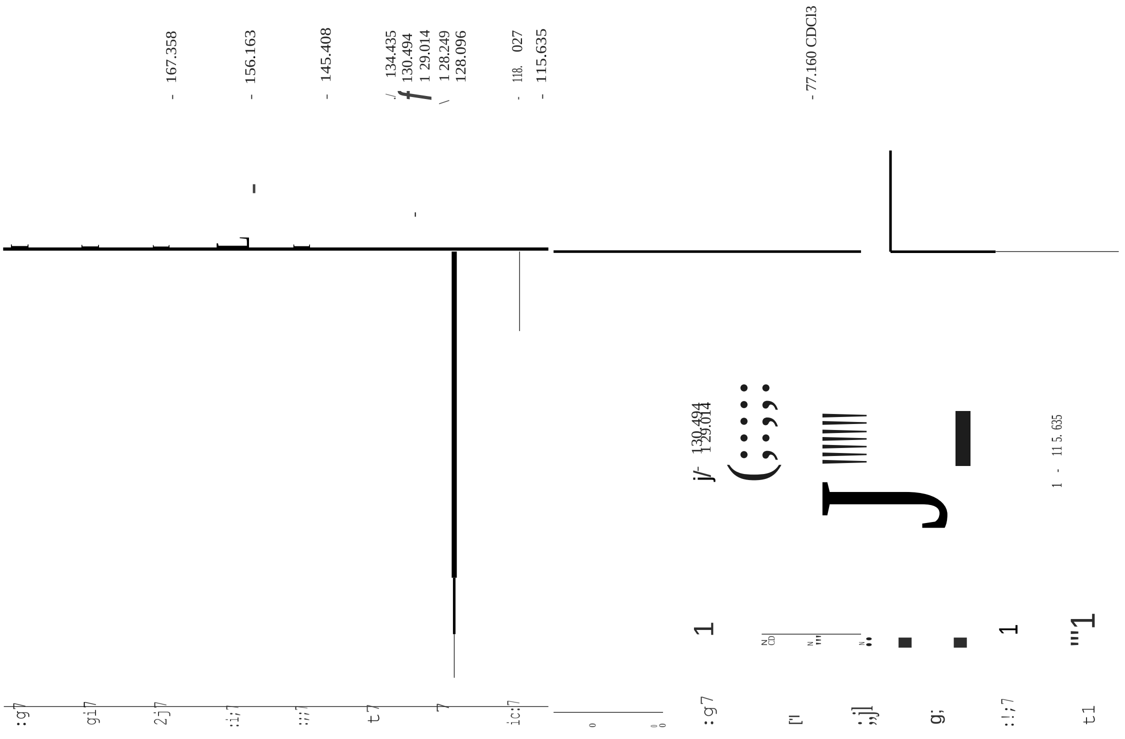

Figure S33. ^{13}C NMR (100MHz, $CDCl_3$) spectrum of 4-hydroxybenzyl cinnamate (**12**)

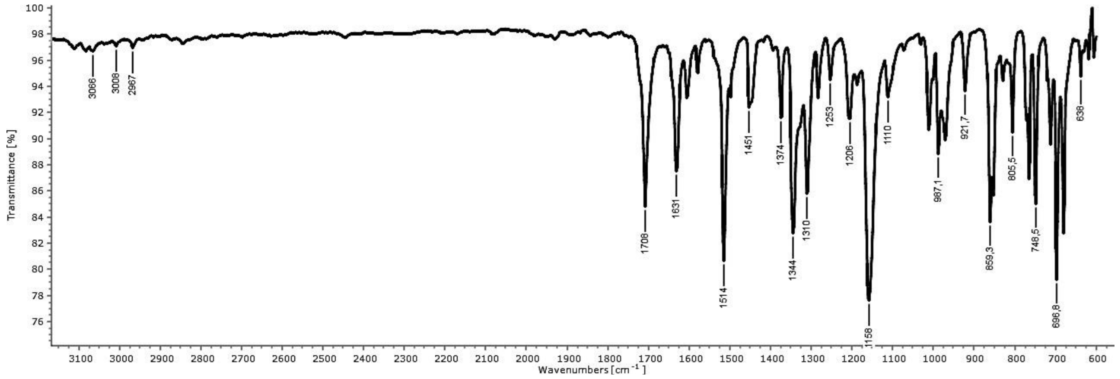

Figure S34. IR ʋmax (KBr, cm^{-1}) spectrum of 4-nitrobenzyl cinnamate (**13**)

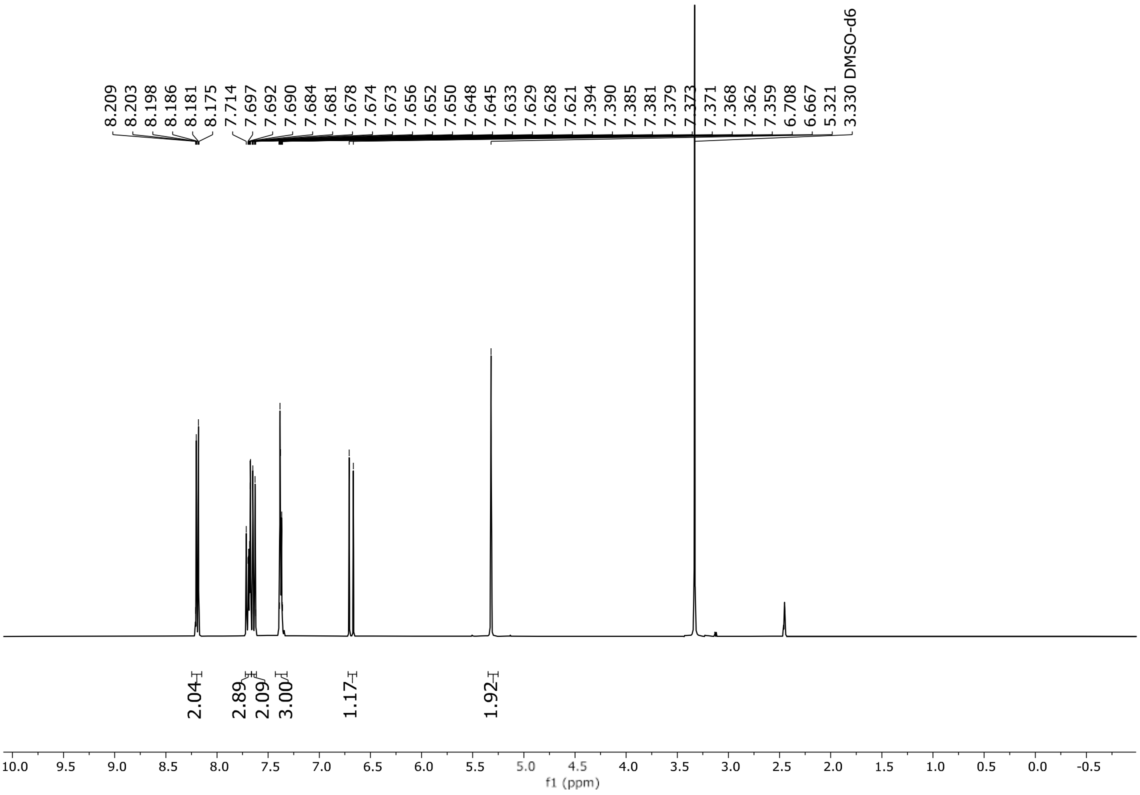

Figure S35. ^{1}H NMR (400MHz, DMSO-d_6) spectrum of 4-nitrobenzyl cinnamate (**13**)

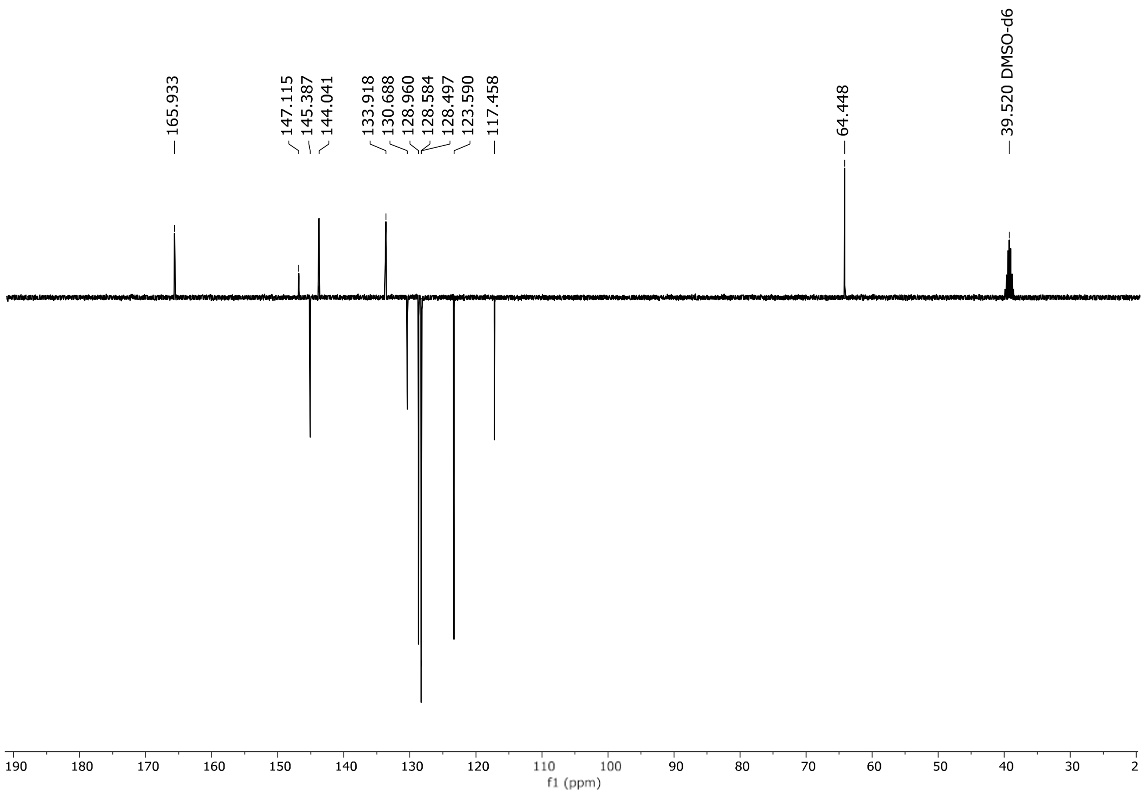

Figure S36. ^{13}C NMR (100MHz, DMSO-d_6) spectrum of 4-nitrobenzyl cinnamate (**13**)

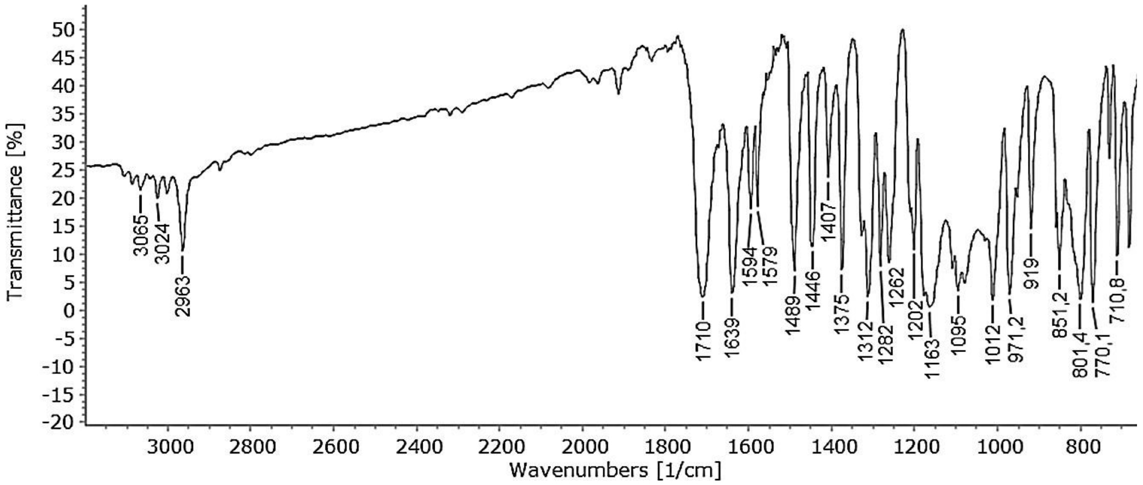

Figure S37. IR υmax (KBr, cm^{-1}) spectrum of 4-chlorobenzyl cinnamate (**14**)

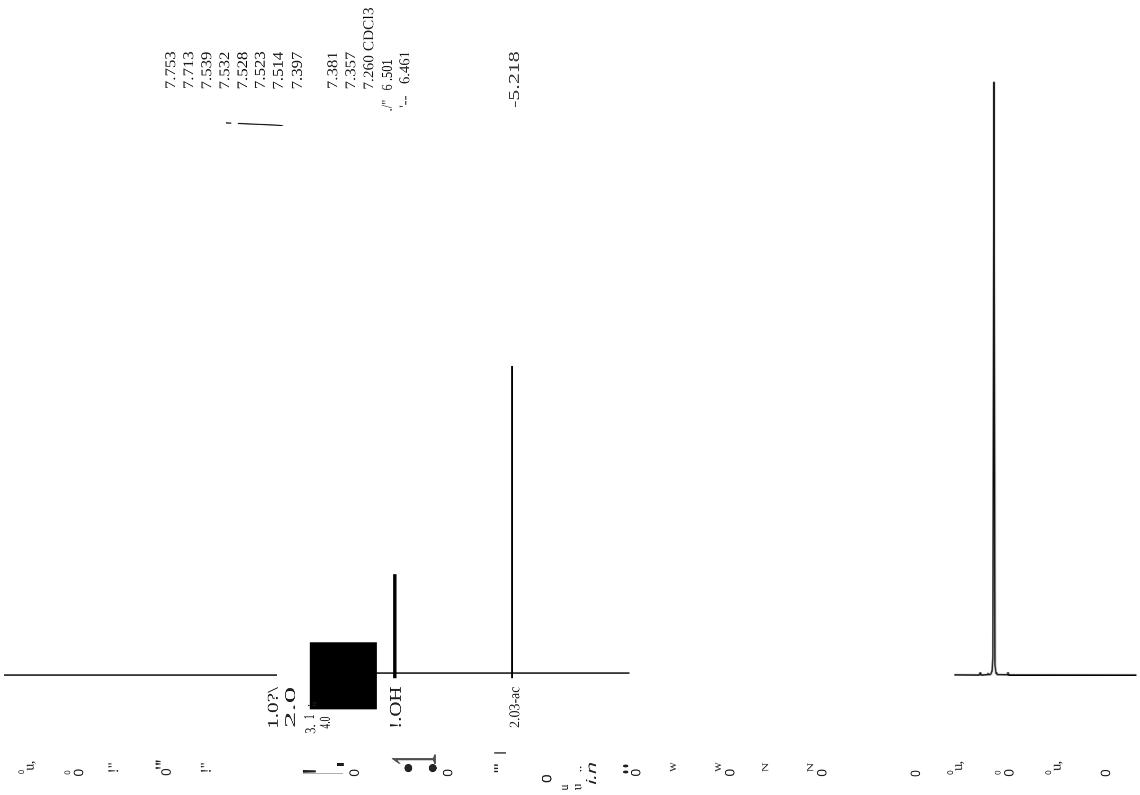

Figure S38. ^{1}H NMR (400MHz, $CDCl_3$) spectrum of 4-chlorobenzyl cinnamate (**14**)

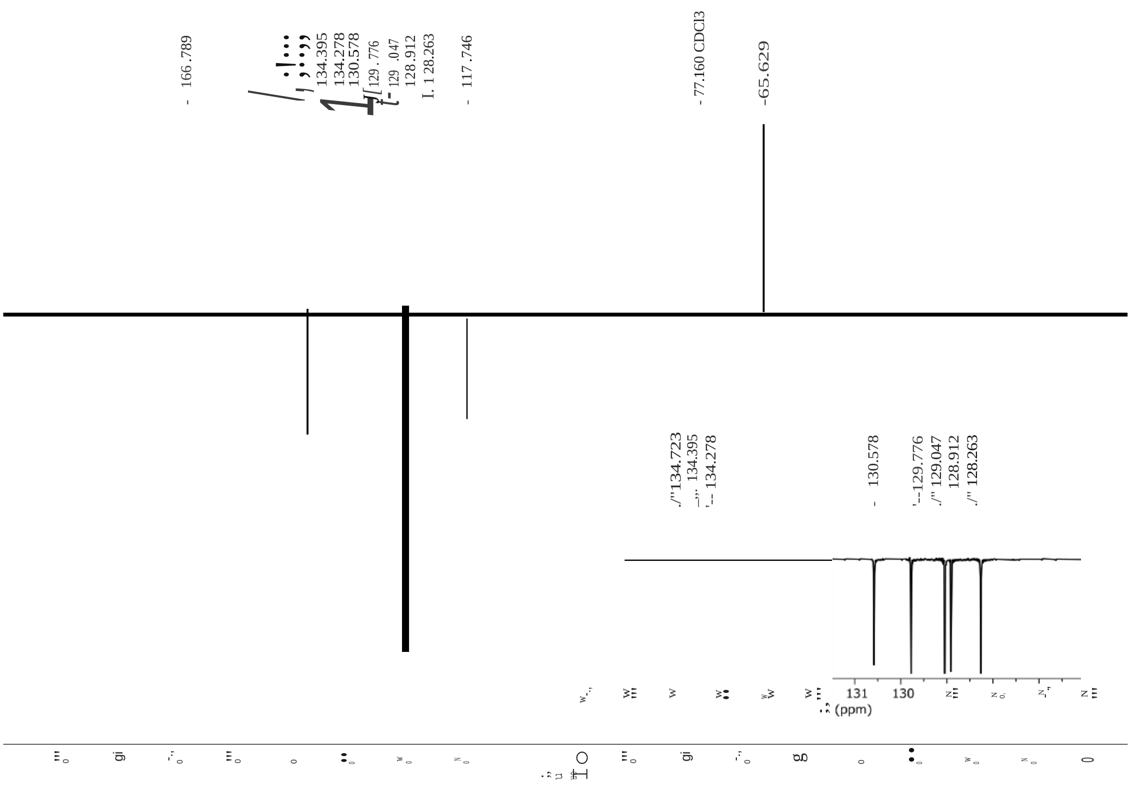

Figure S39. ^{13}C NMR (100MHz, $CDCl_3$) spectrum of 4-chlorobenzyl cinnamate (**14**)

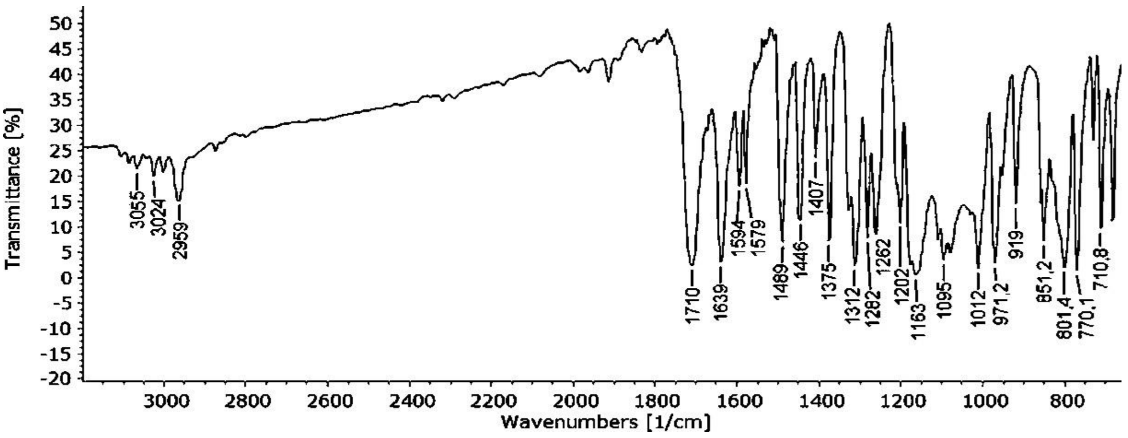

Figure S40. IR υmax (KBr, cm⁻¹) spectrum of piperonyl cinnamate (**15**)

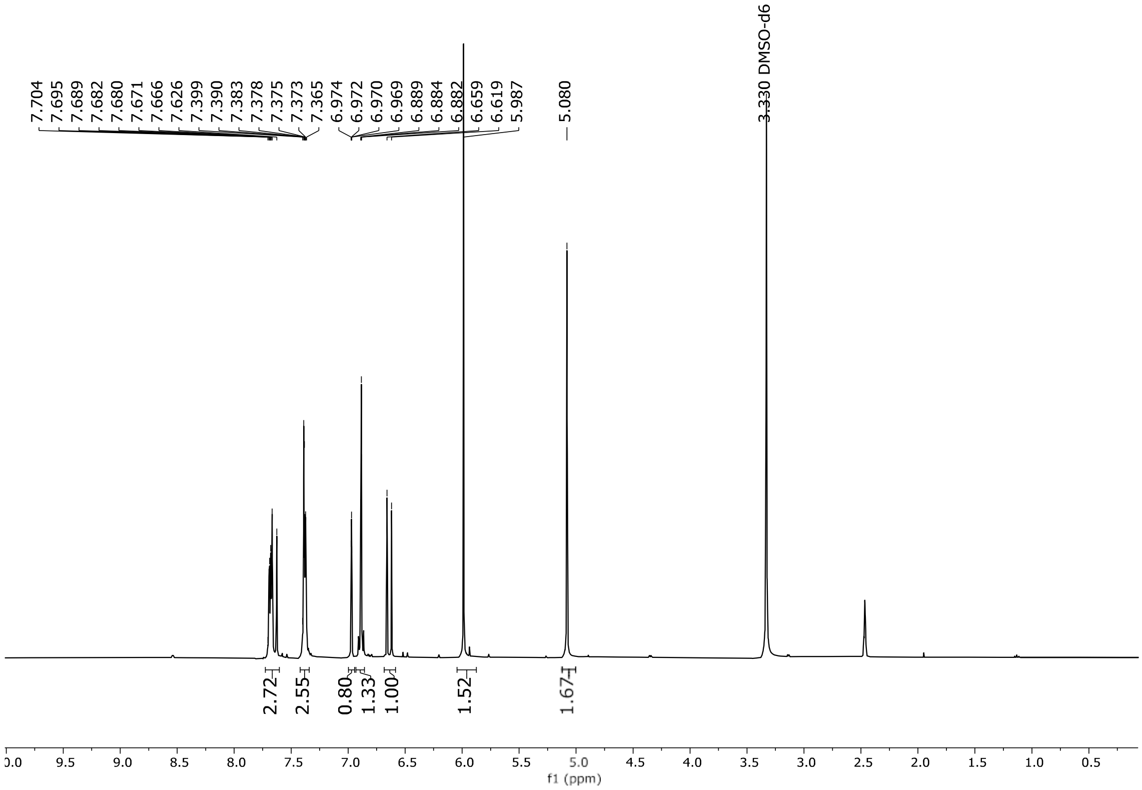

Figure S41. ^{1}H NMR (400MHz, DMSO-d_6) spectrum of piperonyl cinnamate (**15**)

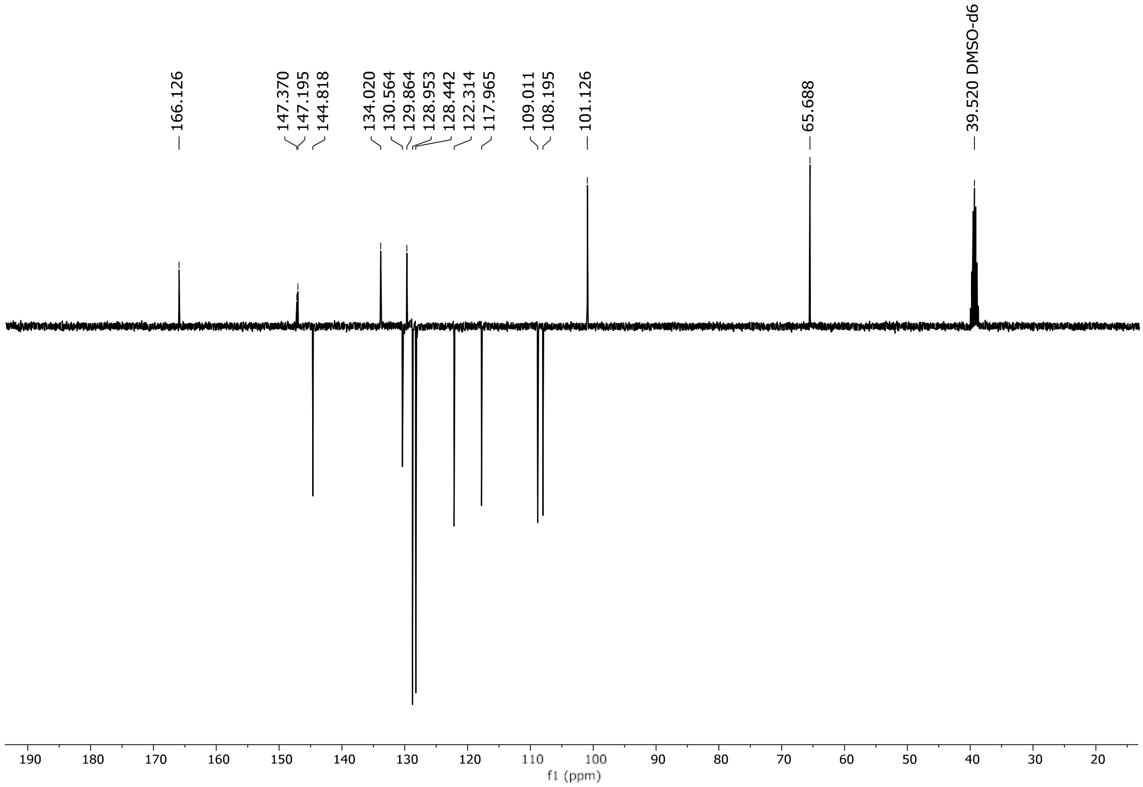

Figure S42. ^{13}C NMR (100MHz, DMSO-d_6) spectrum of piperonyl cinnamate (**15**)

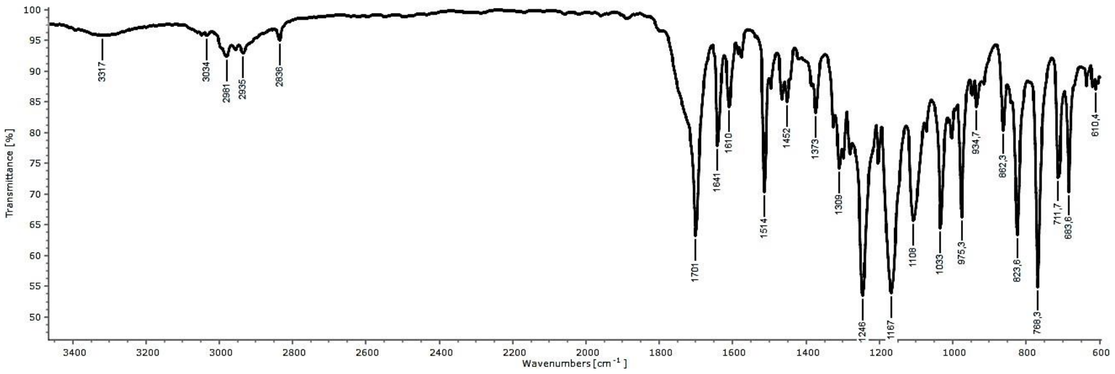

Figure S43. IR υmax (KBr, cm^{-1}) spectrum of 4-hydroxy-3-methoxy-benzyl cinnamate (**16**)

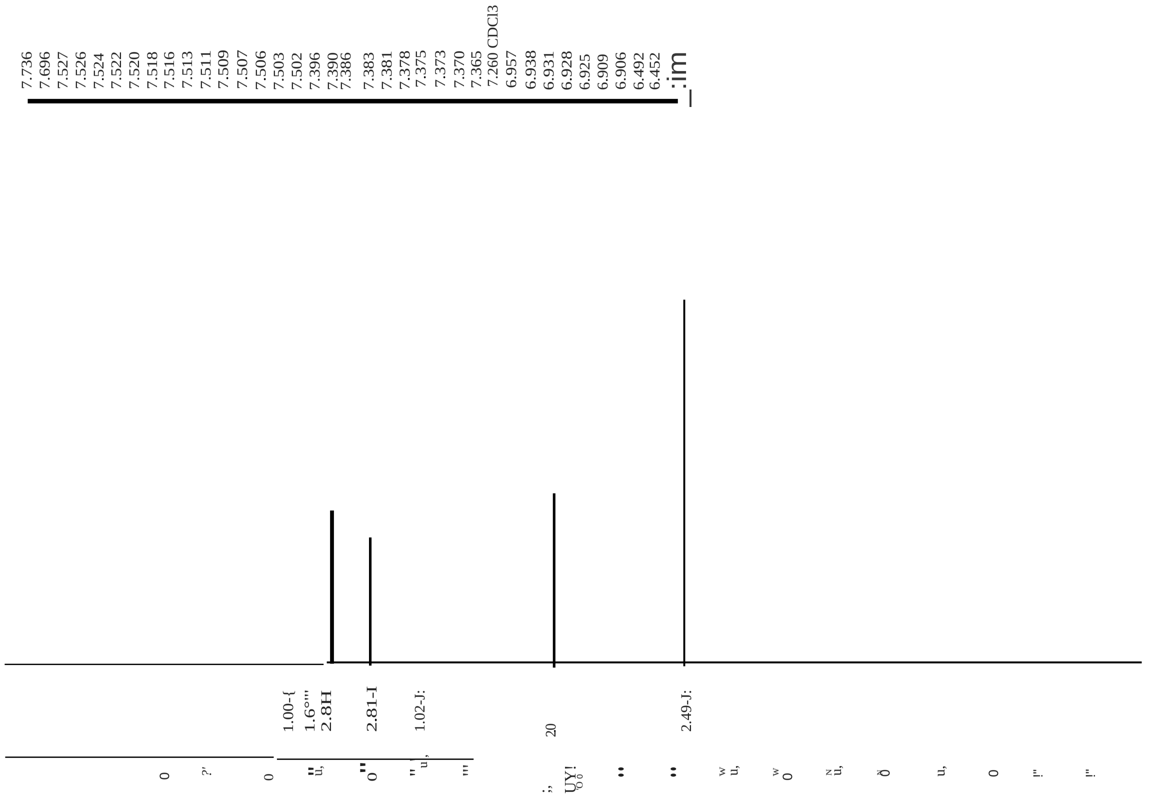

Figure S44. ^{1}H NMR (400MHz, $CDCl_3$) spectrum of 4-hydroxy-3-methoxy-benzyl cinnamate (**16**)

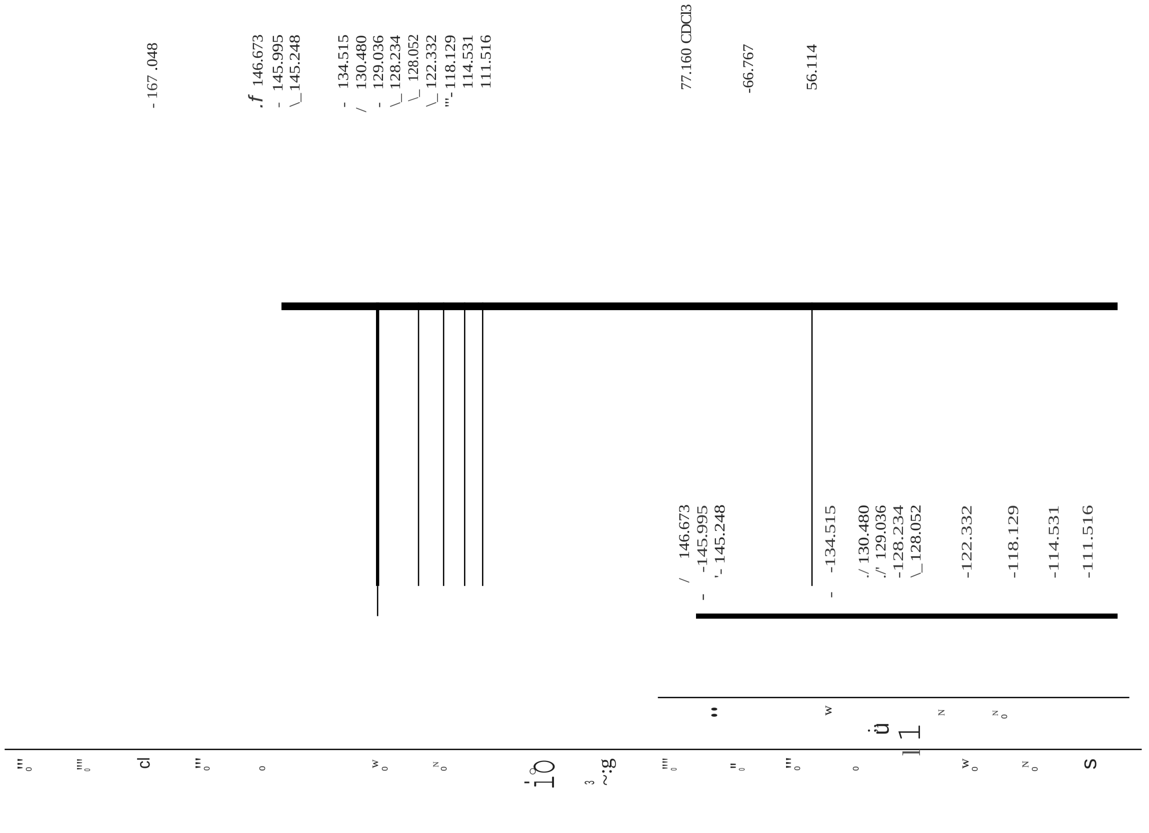

Figure S45. ^{13}C NMR (100MHz, $CDCl_3$) spectrum of 4-hydroxy-3-methoxy-benzyl cinnamate (**16**)

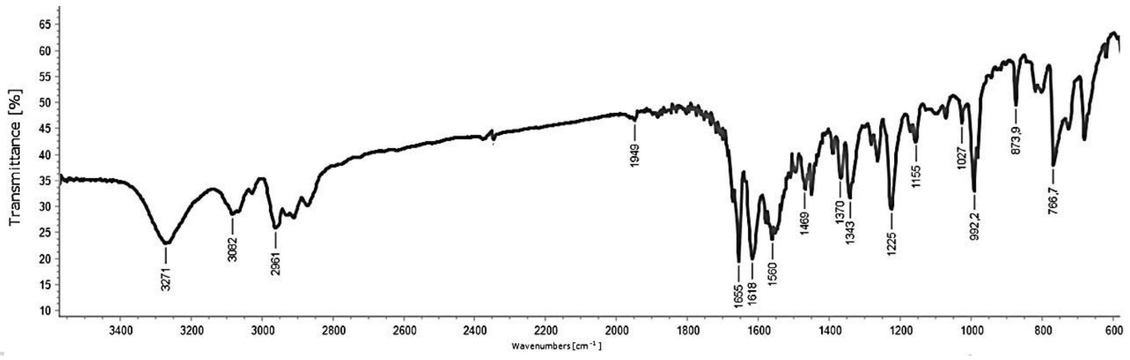

Figure S46. IR υmax (KBr, cm^{-1}) spectrum of isobutylcinnamamide (**17**)

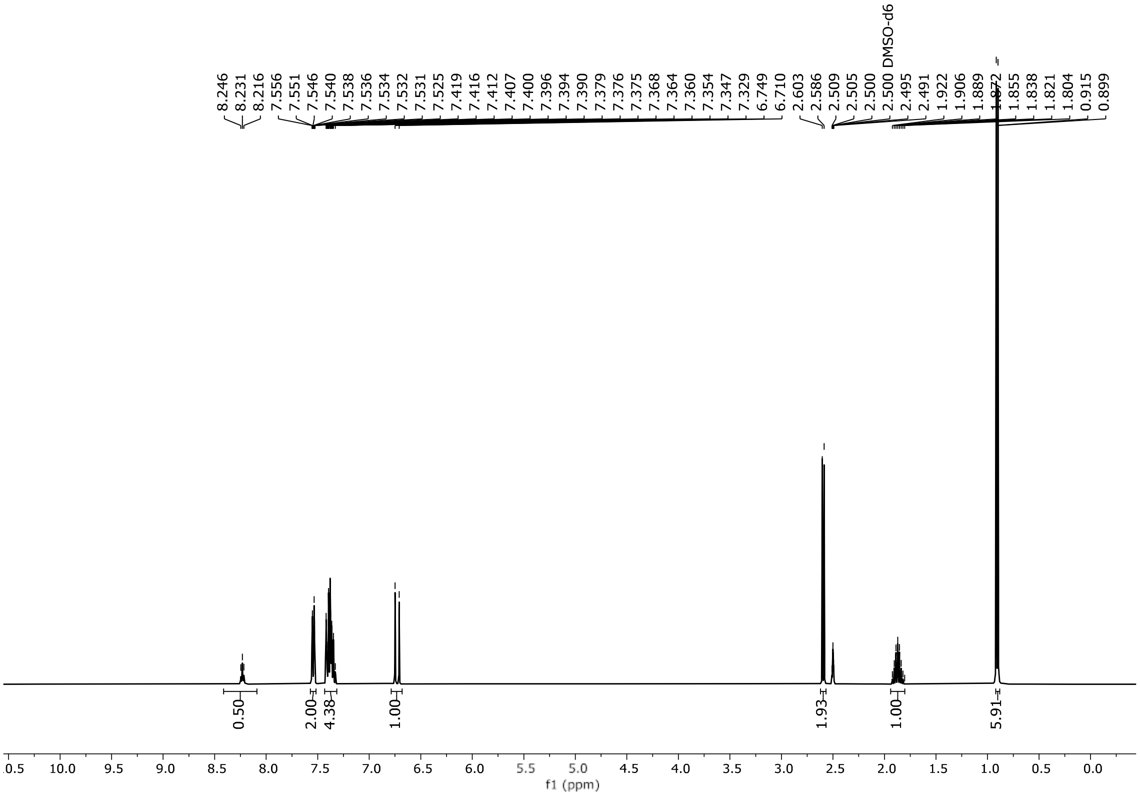

Figure S47. ^{1}H NMR (400MHz, DMSO-d$_6$) spectrum of isobutylcinnamamide (**17**)

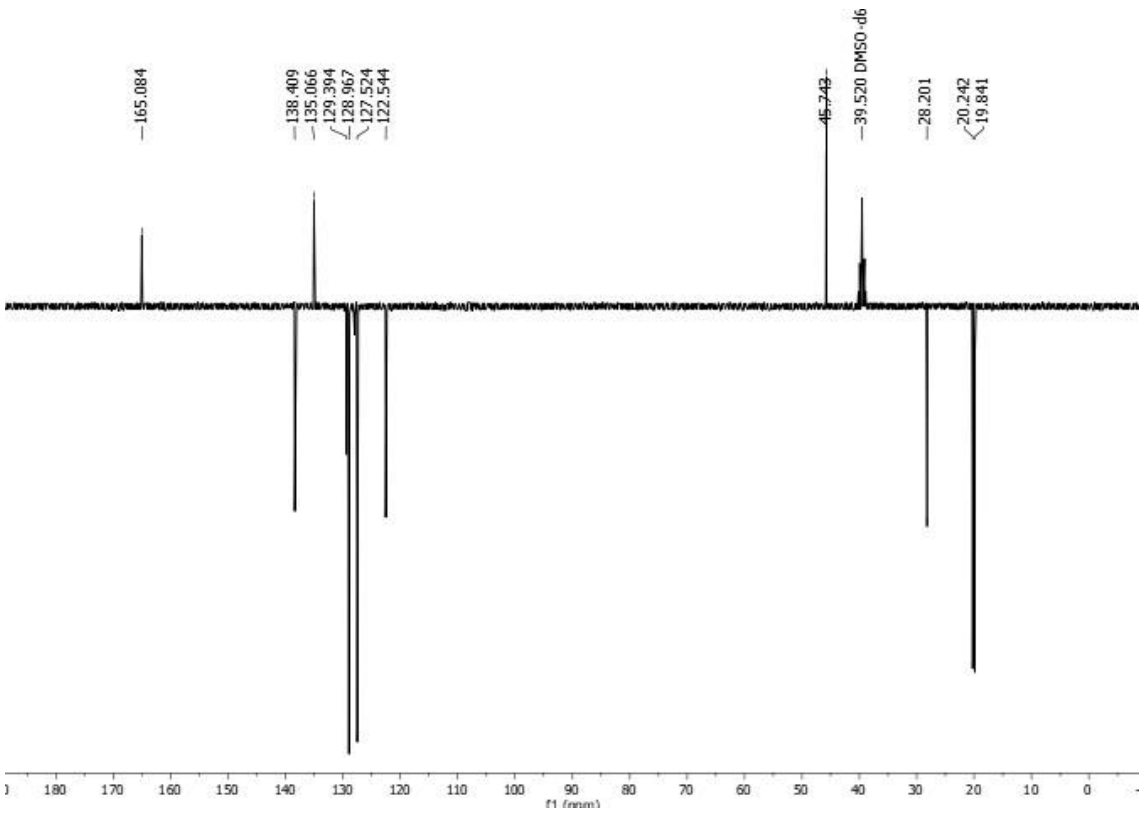

Figure S48. ^{13}C NMR (100MHz, DMSO-d_6) spectrum of isobutylcinnamamide (**17**)

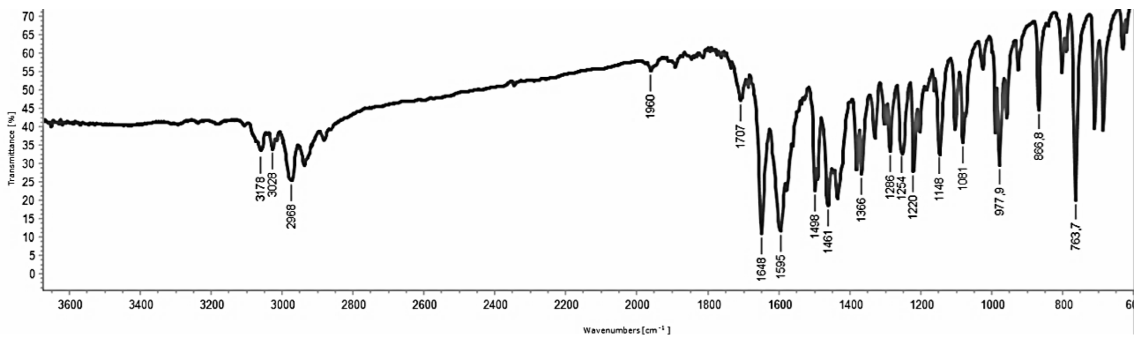

Figure S49. IR υmax (KBr, cm^{-1}) spectrum of diethylcinnamamide (**18**)

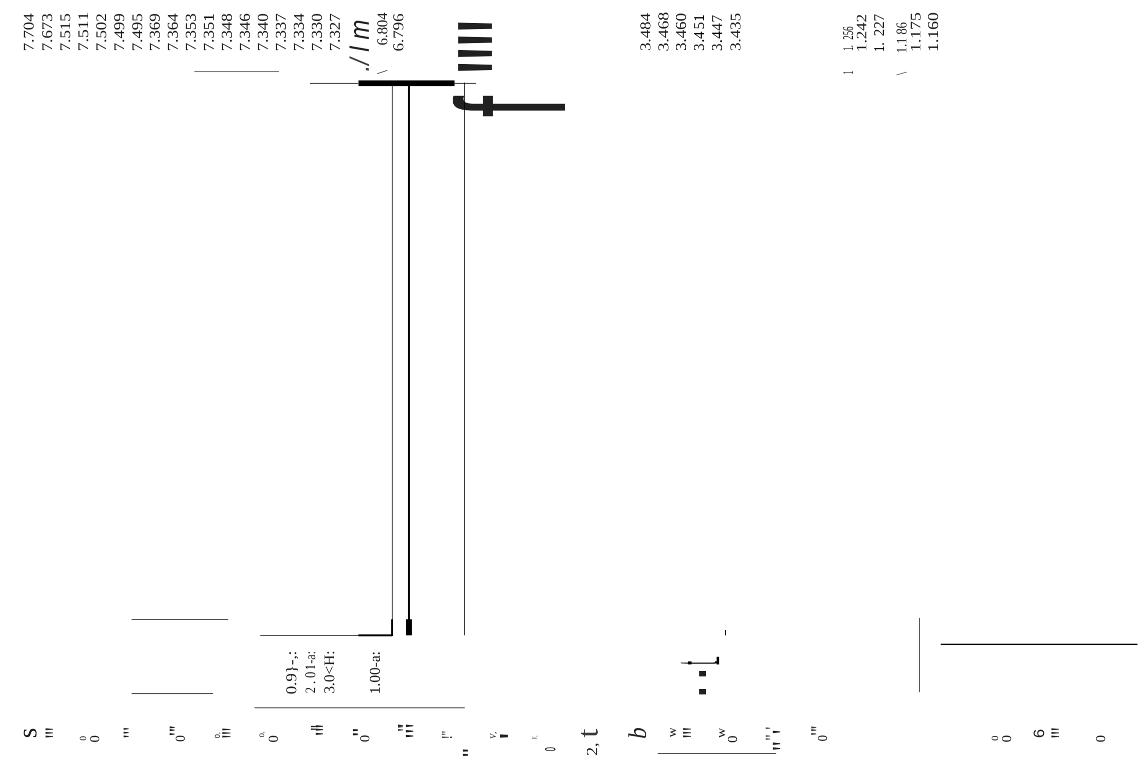

Figure S50. ^{1}H NMR (500MHz, $CDCl_3$) spectrum of diethylcinnamamide (**18**)

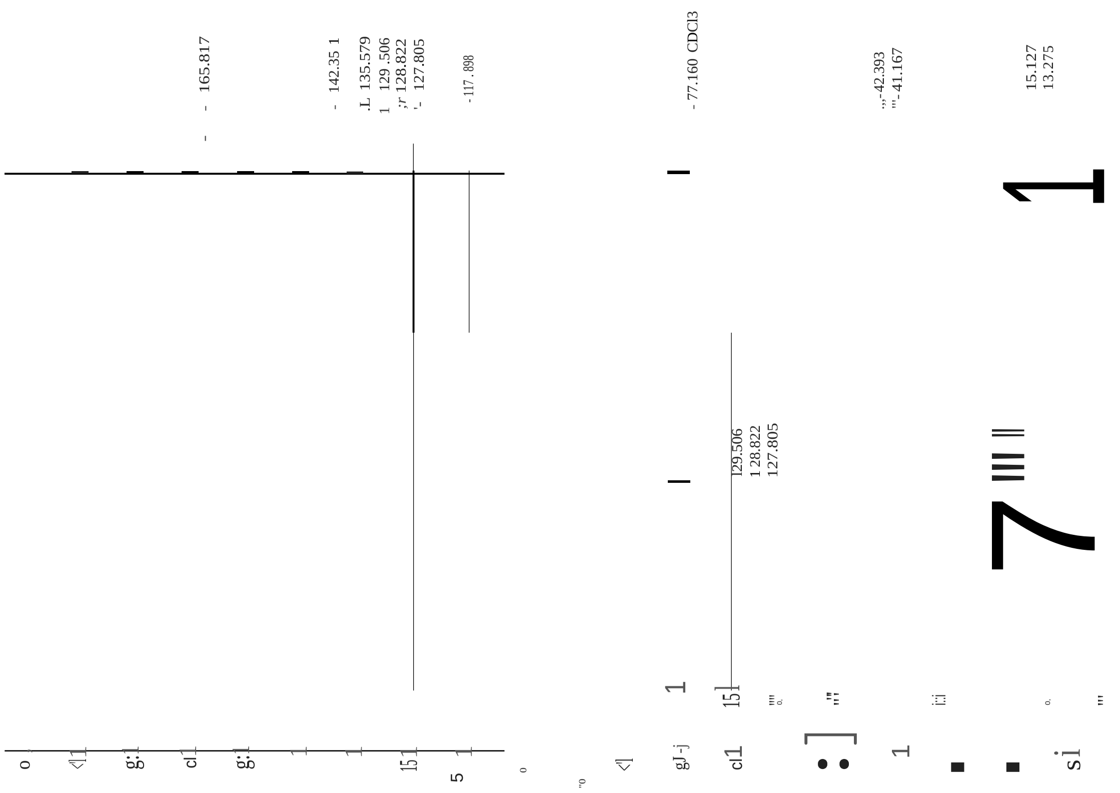

Figure S51. ^{13}C NMR (125 MHz, $CDCl_3$) spectrum of diethylcinnamamide (**18**)

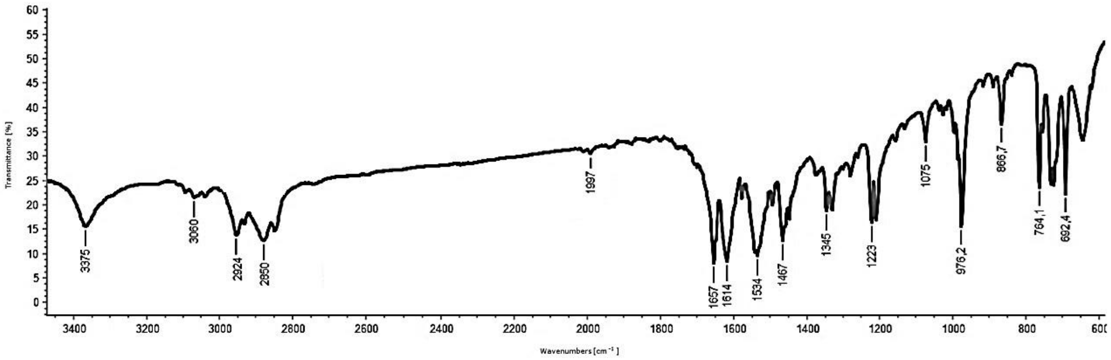

Figure S52. IR υmax (KBr, cm^{-1}) spectrum of butylcinnamamide (**19**)

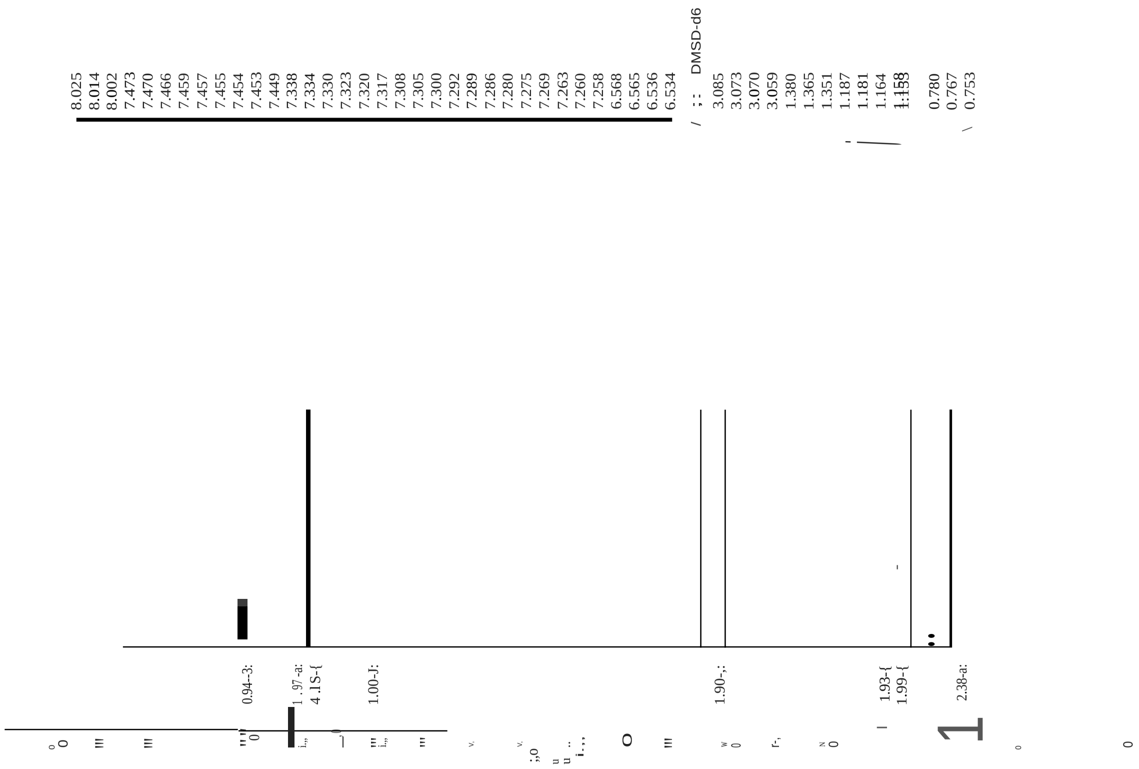

Figure S53. ^{1}H NMR (500MHz, DMSO-d_6) spectrum of butylcinnamamide (**19**)

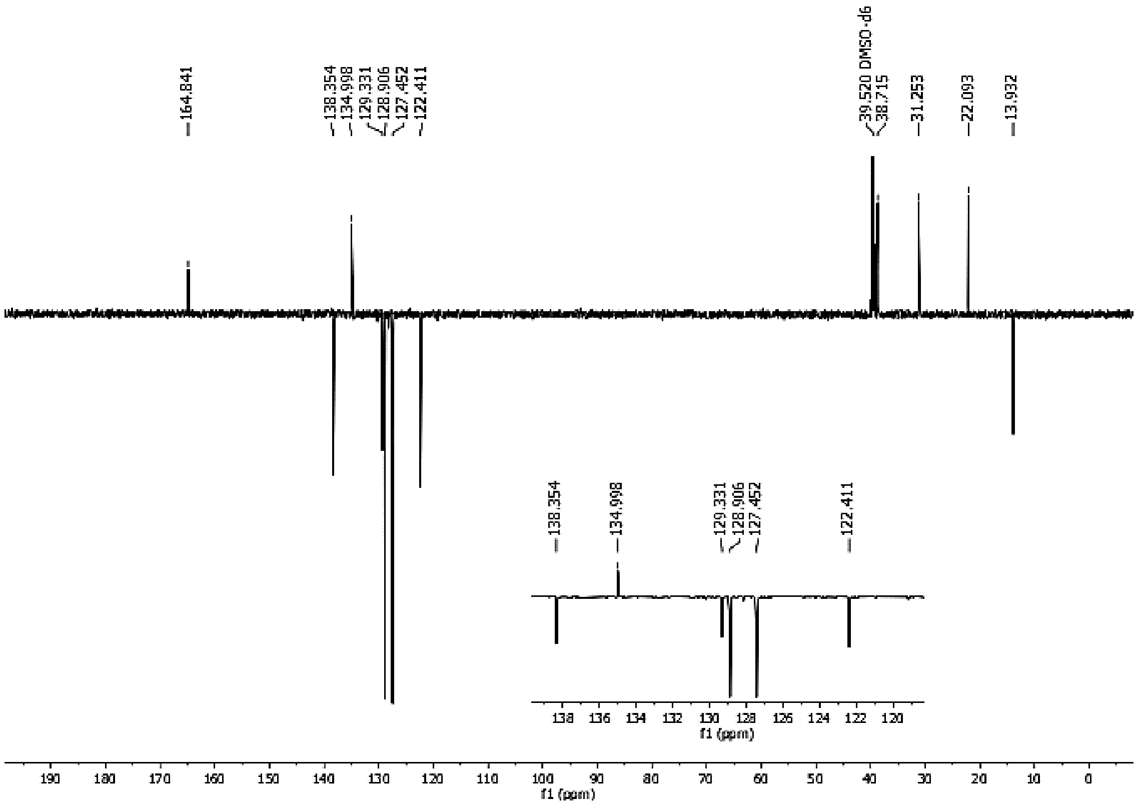

Figure S54. ^{13}C NMR (125 MHz, DMSO-d_6) spectrum of butylcinnamamide (**19**)

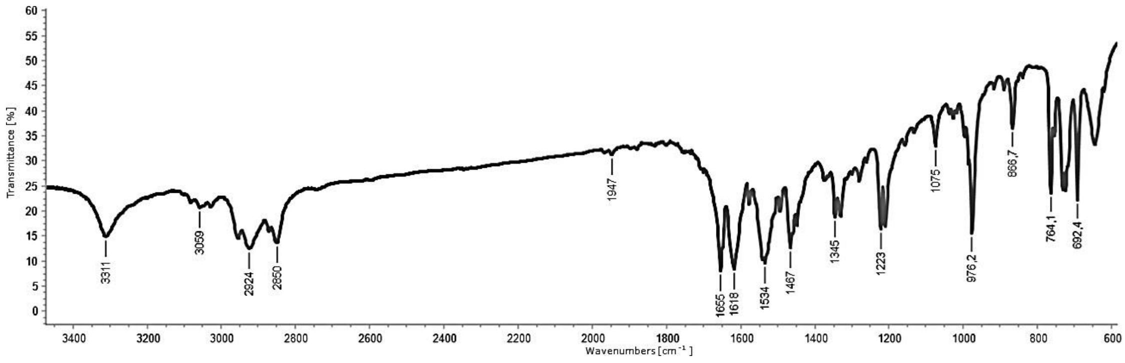

Figure S55. IR υmax (KBr, cm^{-1}) spectrum of hexylcinnamamide (**20**)

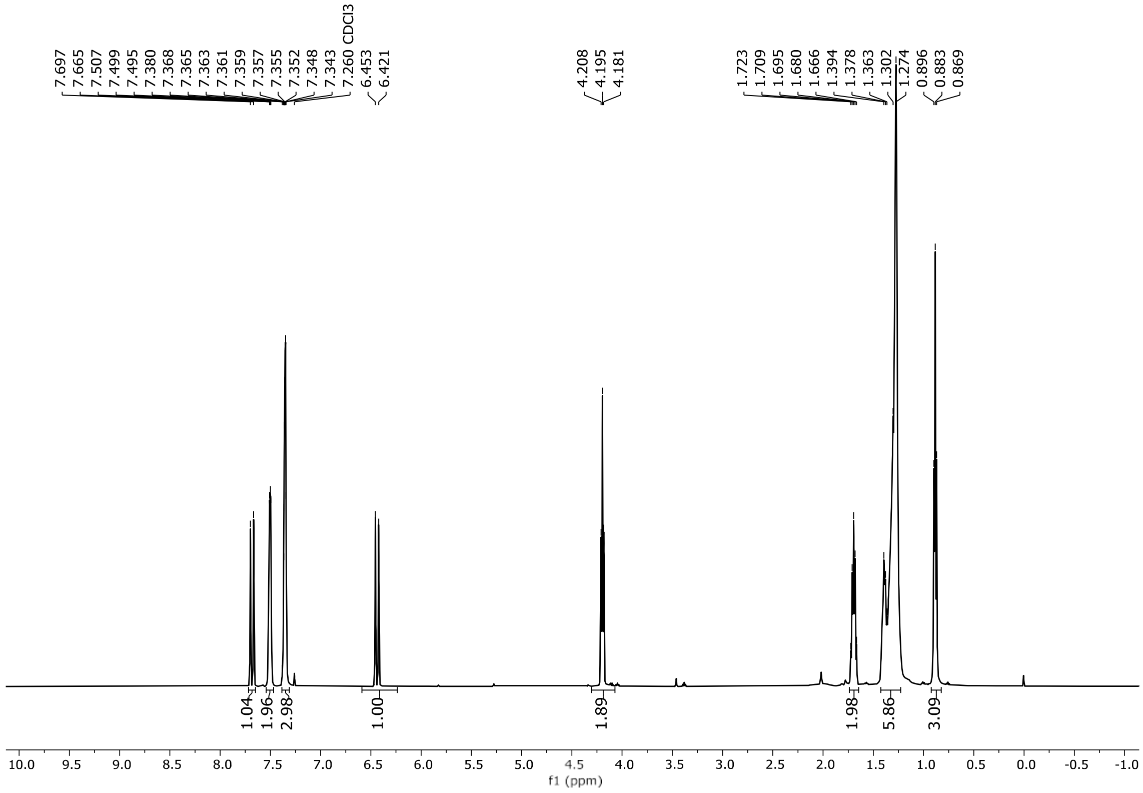

Figure S56. ^{1}H NMR (400MHz, DMSO-d$_6$) spectrum of hexylcinnamamide (**20**)

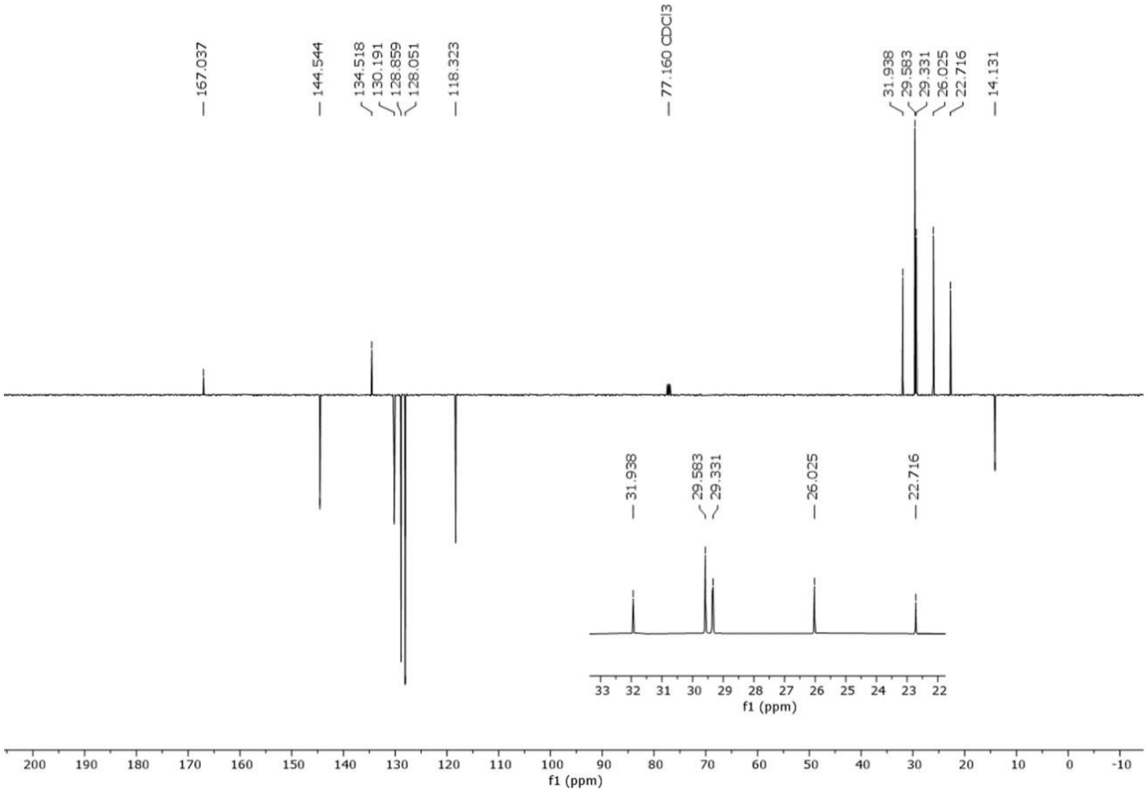

Figure S57. ^{13}C NMR (100MHz, DMSO-d$_6$) spectrum of hexylcinnamamide (**20**)

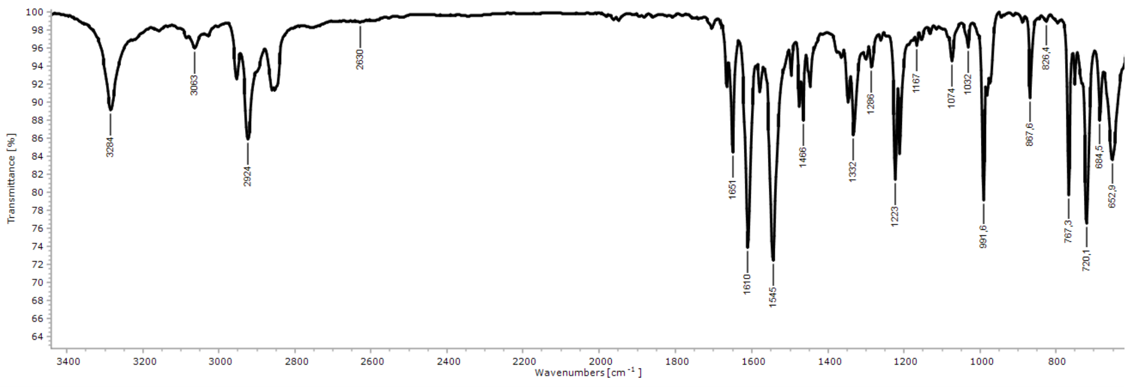

Figure S58. IR υmax (KBr, cm^{-1}) spectrum of octylcinnamamide (**21**)

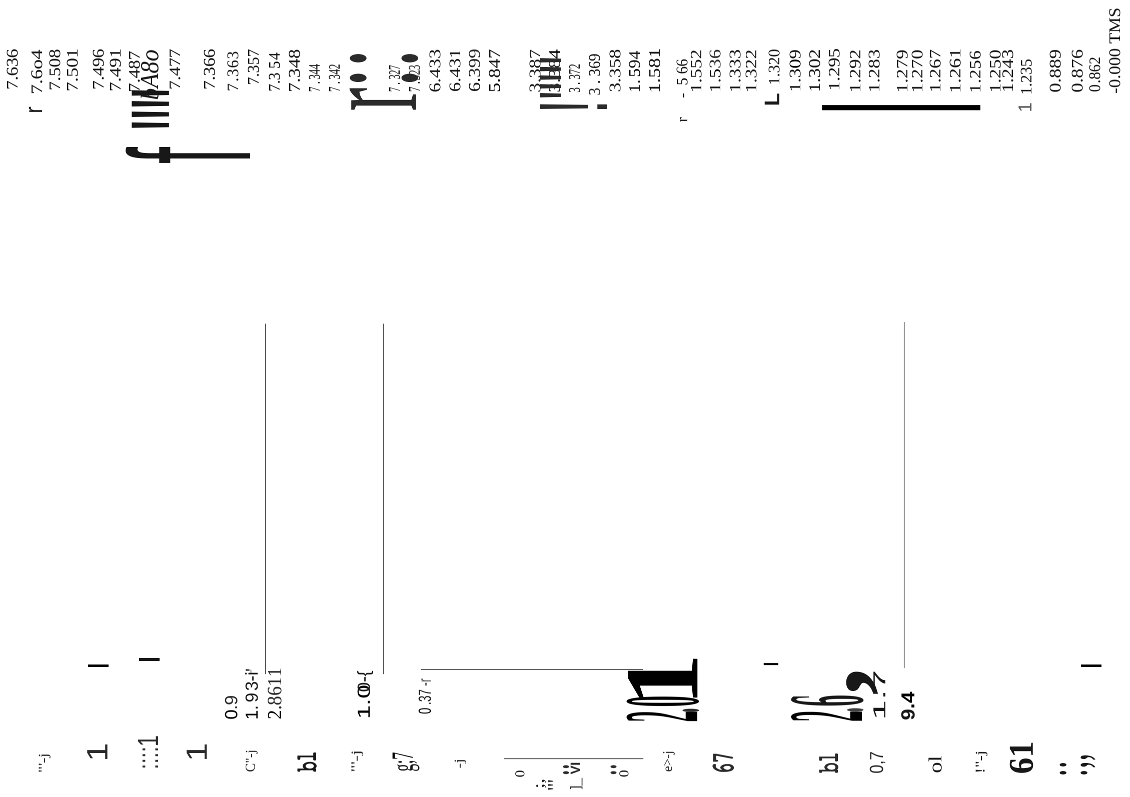

Figure S59. ^{1}H NMR (500 MHz, DMSO-d_6) spectrum of octylcinnamamide (**21**)

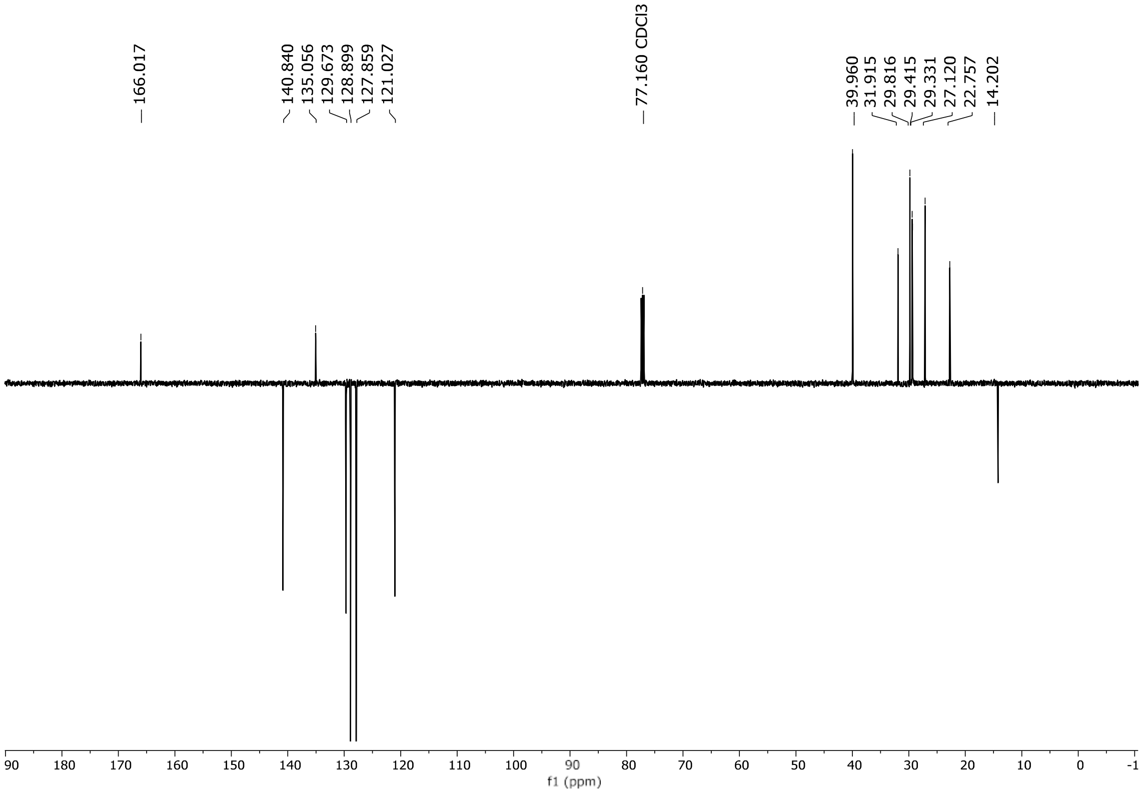

Figure S60. ^{13}C NMR (125 MHz, DMSO-d$_6$) spectrum of octylcinnamamide (**21**)

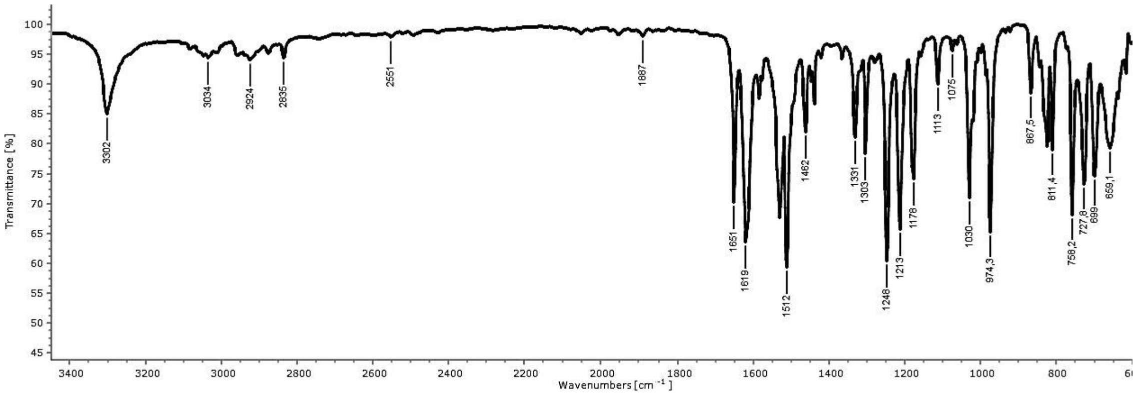

Figure S61. IR υmax (KBr, cm^{-1}) spectrum of decylcinnamamide (**22**)

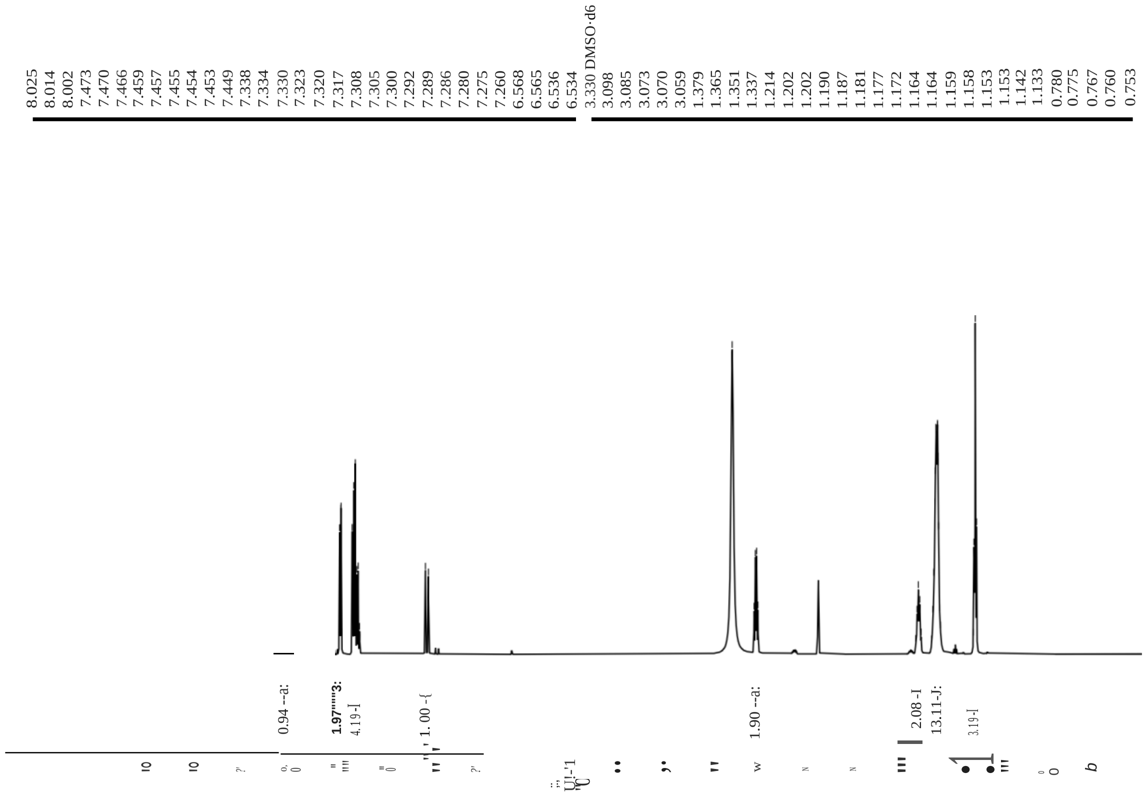

Figure S62. ^{1}H NMR (500MHz, $CDCl_3$) spectrum of decylcinnamamide (**22**)

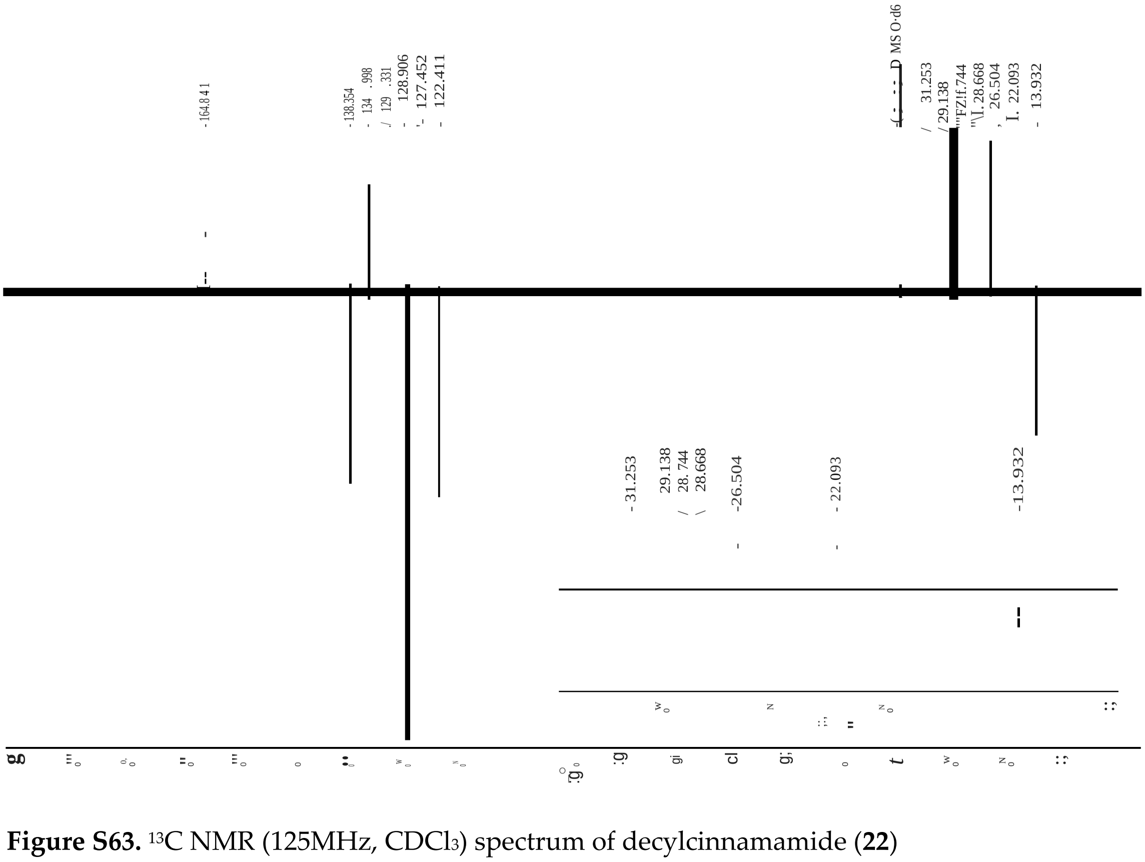

Figure S63. ^{13}C NMR (125MHz, $CDCl_3$) spectrum of decylcinnamamide (**22**)

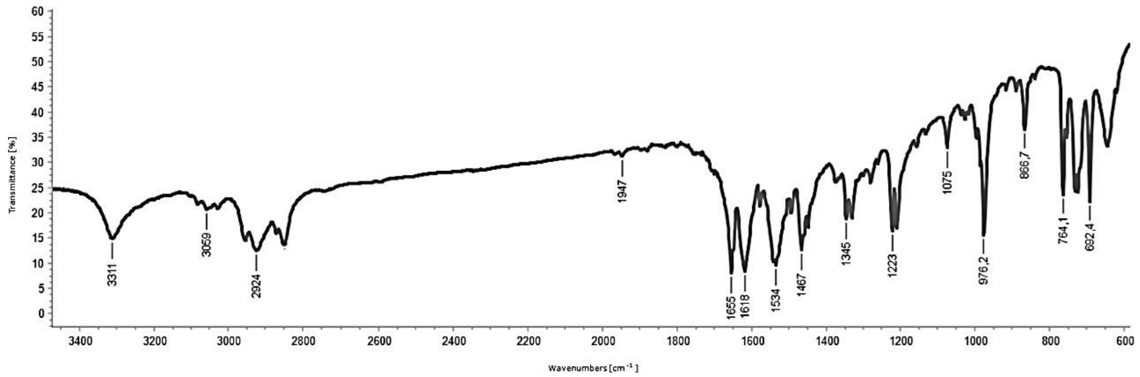

Figure S64. IR υmax (KBr, cm^{-1}) spectrum of hexadecylcinnamamide (**23**)

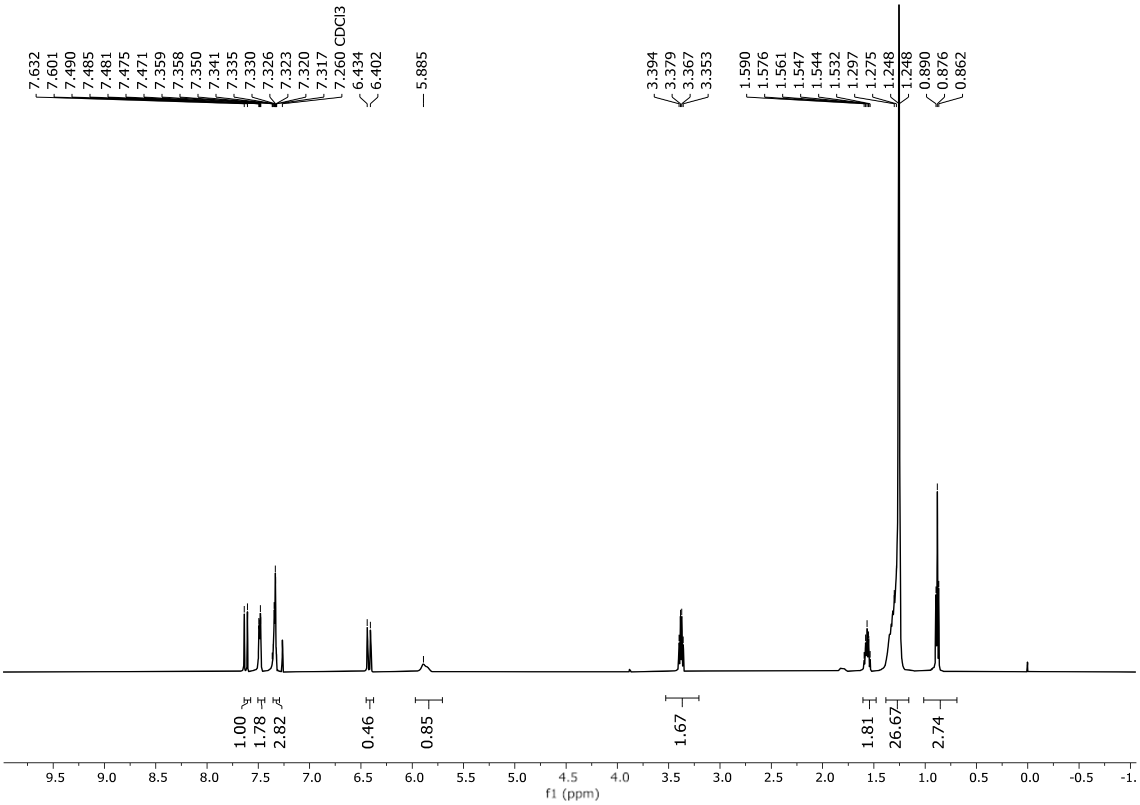

Figure S65. ^{1}H NMR (500MHz, $CDCl_3$) spectrum of hexadecylcinnamamide (**23**)

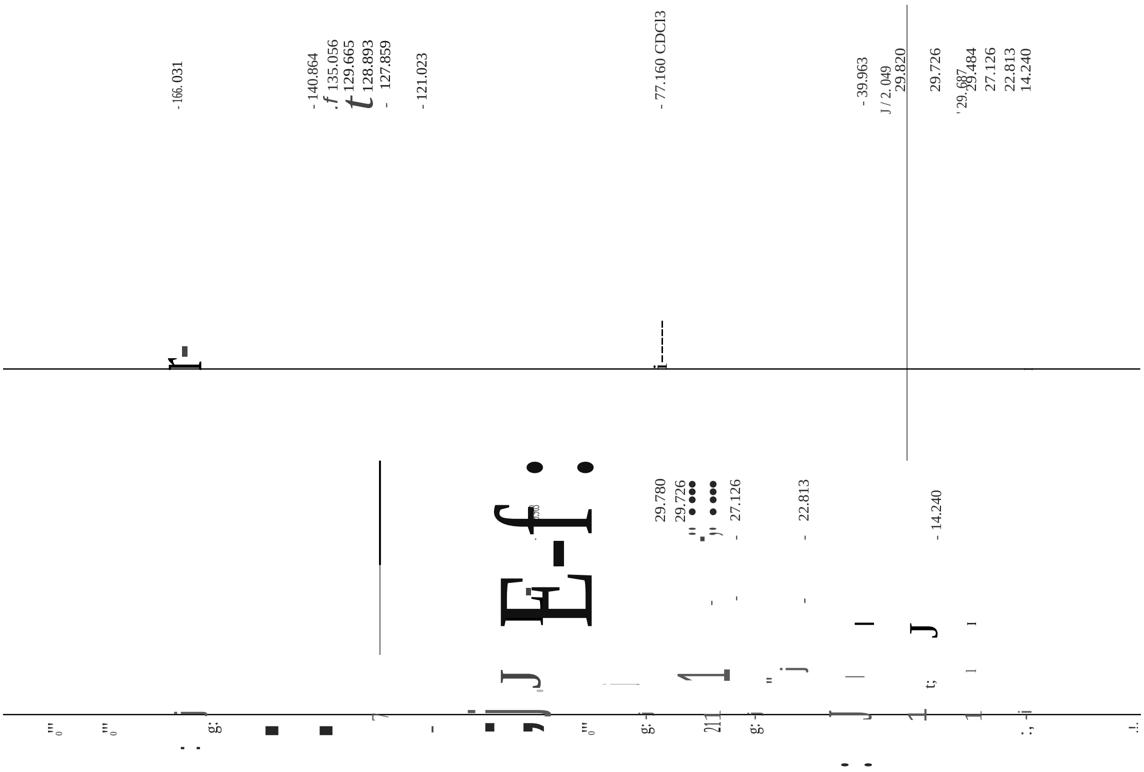

Figure S66. ^{13}C NMR (125 MHz, $CDCl_3$) spectrum of hexadecylcinnamamide (**23**)

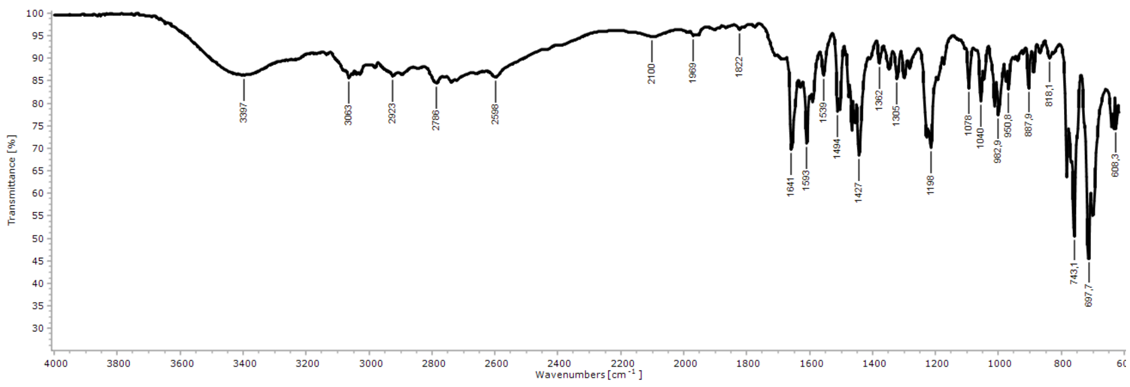

Figure S67. IR υmax (KBr, cm^{-1}) spectrum of oleylcinnamamide (**24**)

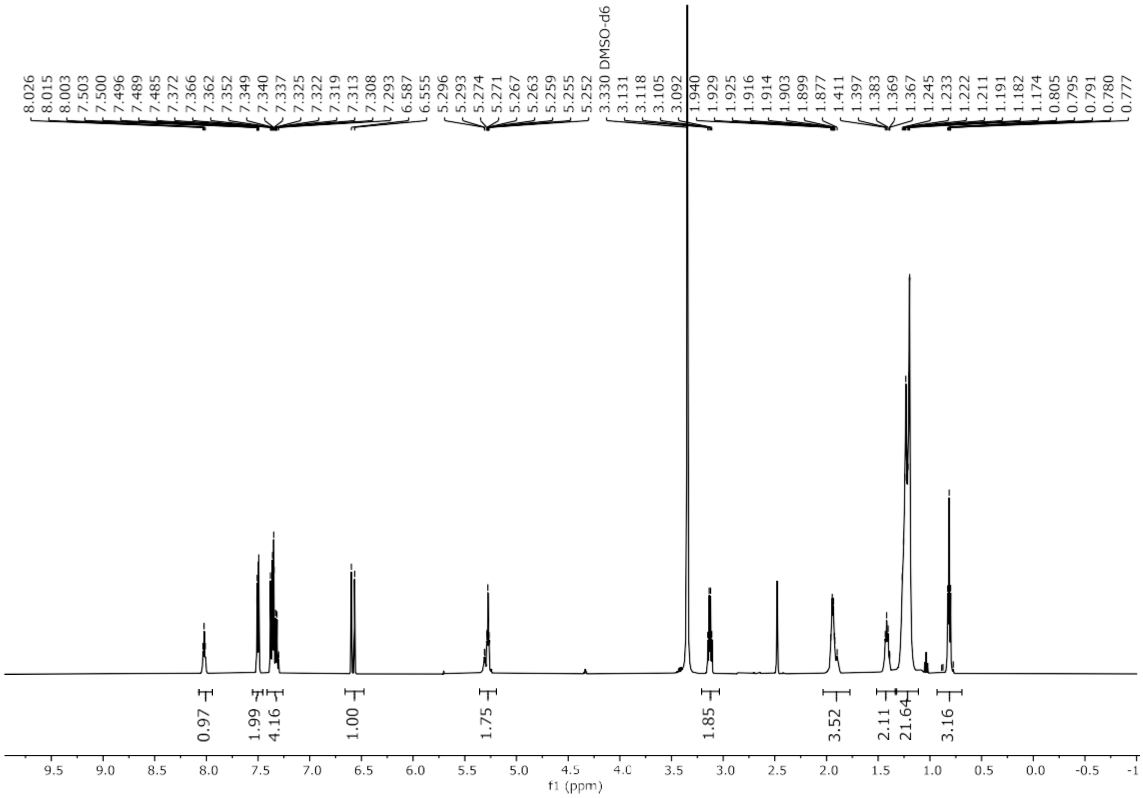

Figure S68. ^{1}H NMR (500MHz, $CDCl_3$) spectrum of oleylcinnamamide (**24**)

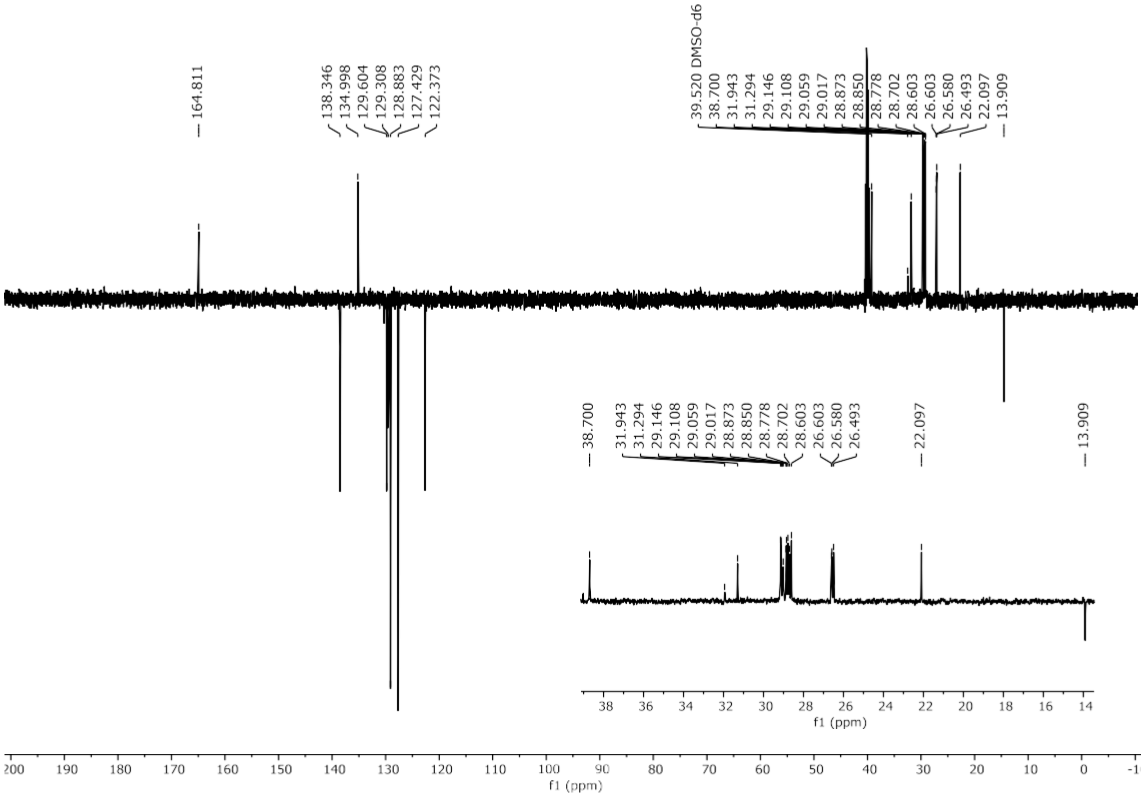

Figure S69. ^{13}C NMR (125 MHz, $CDCl_3$) spectrum of oleylcinnamamide (**24**)

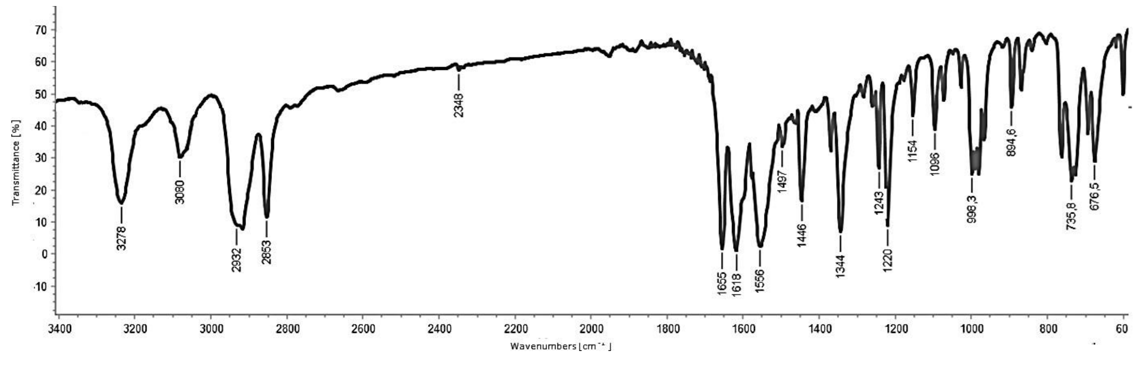

Figure S70. IR υmax (KBr, cm^{-1}) spectrum of cyclohexylcinnamamide (**25**)

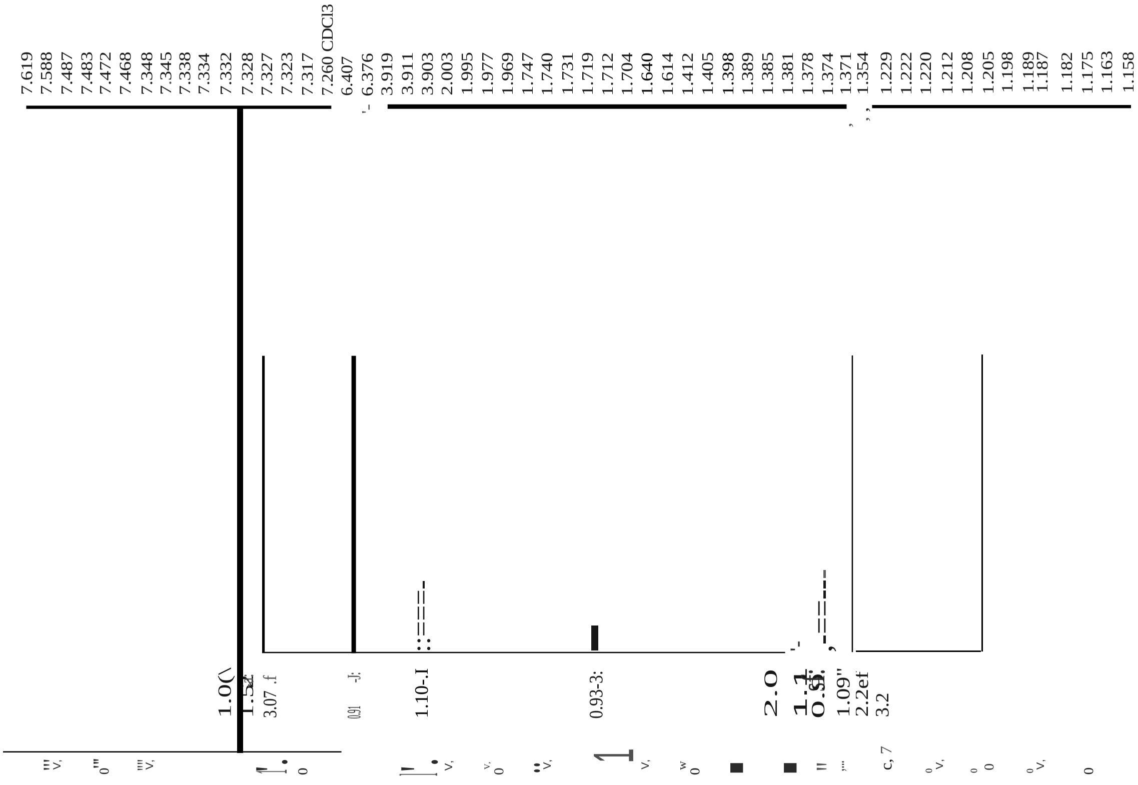

Figure S71. ^{1}H NMR (500MHz, $CDCl_3$) spectrum of cyclohexylcinnamamide (**25**)

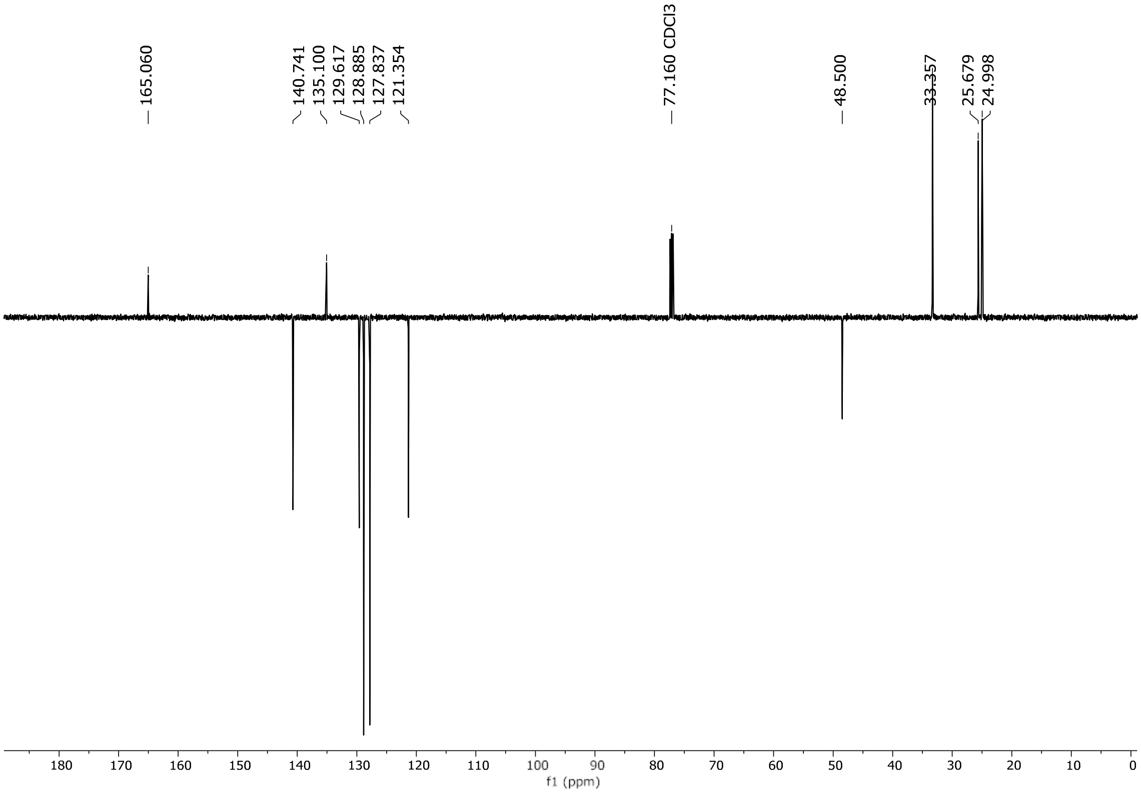

Figure S72. ^{13}C NMR (125 MHz, $CDCl_3$) spectrum of cyclohexylcinnamamide (**25**)

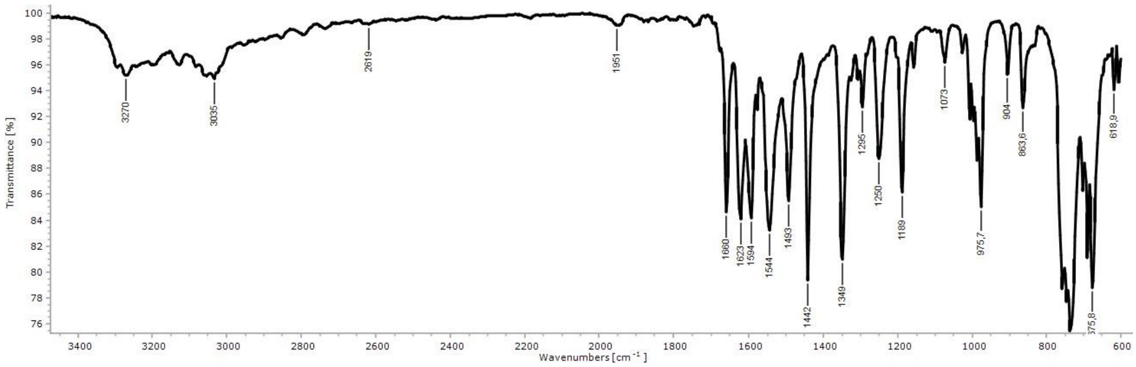

Figure S73. IR υmax (KBr, cm^{-1}) spectrum of phenylcinnamamide (**26**)

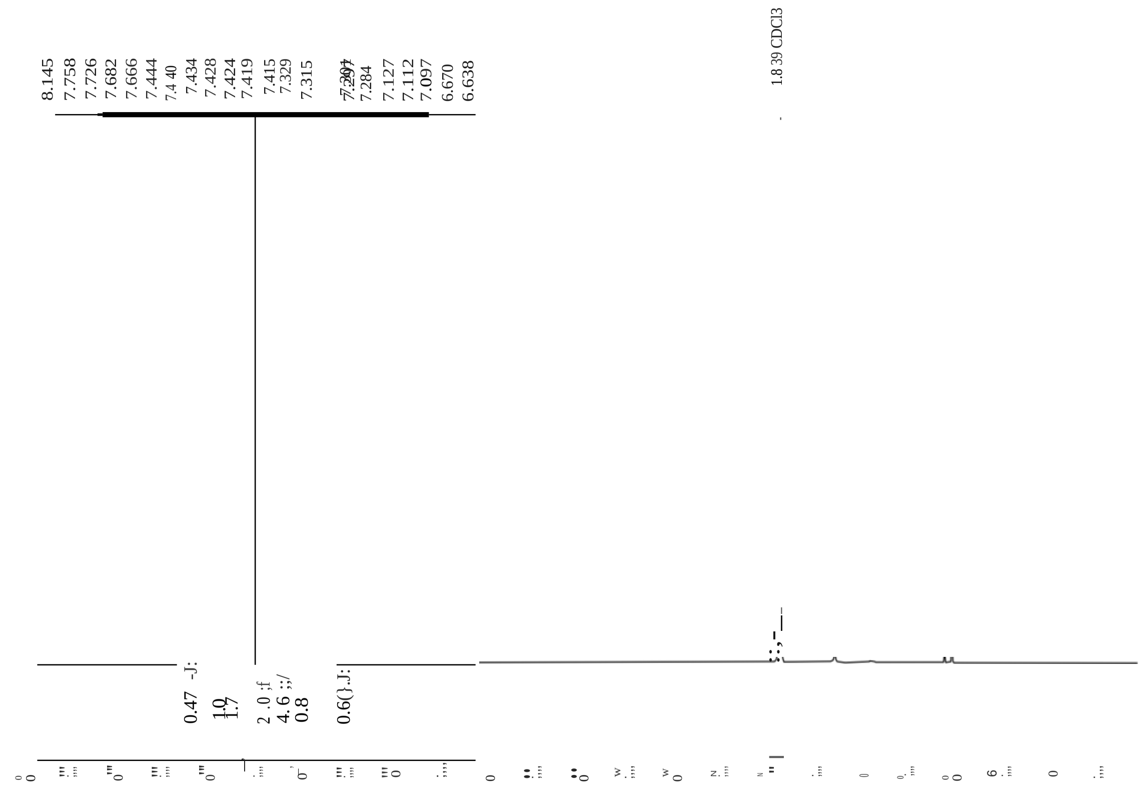

Figure S74. ^1H NMR (400MHz, $CDCl_3$) spectrum of phenylcinnamamide (**26**)

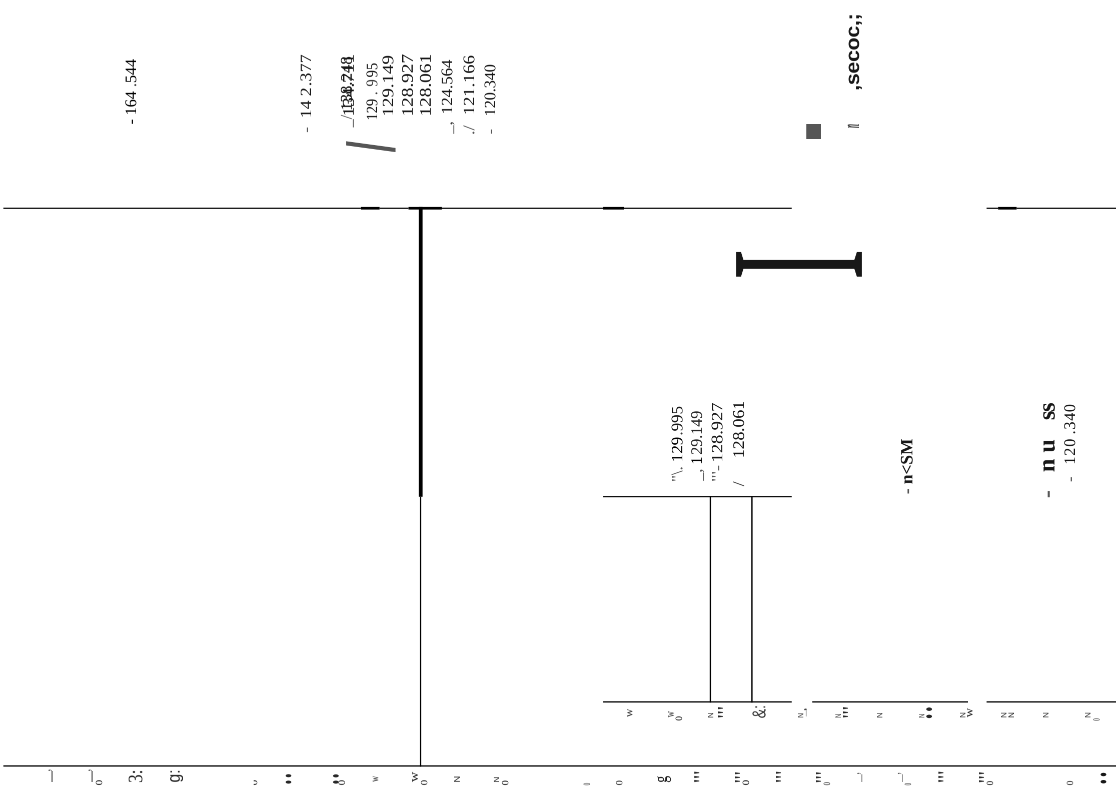

Figure S75. ^{13}C NMR (100MHz, $CDCl_3$) spectrum of phenylcinnamamide (**26**)

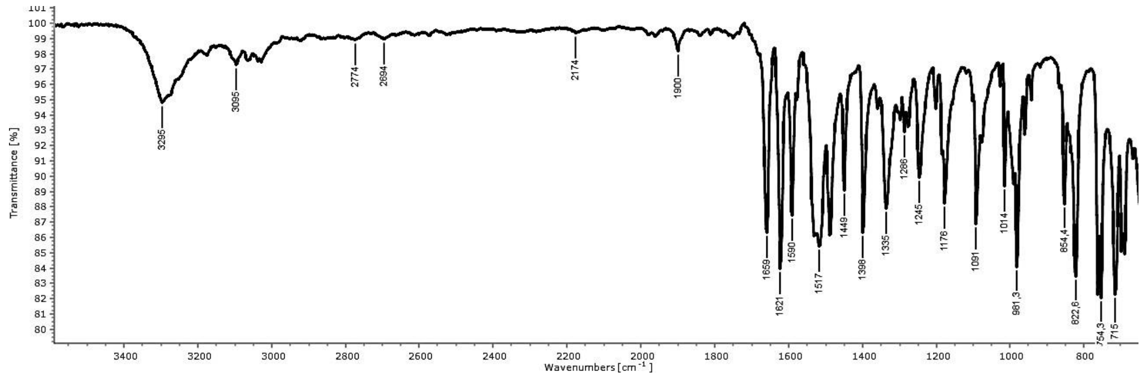

Figure S76. IR υmax (KBr, cm^{-1}) spectrum of 4-chlorophenylcinnamamide (**27**)

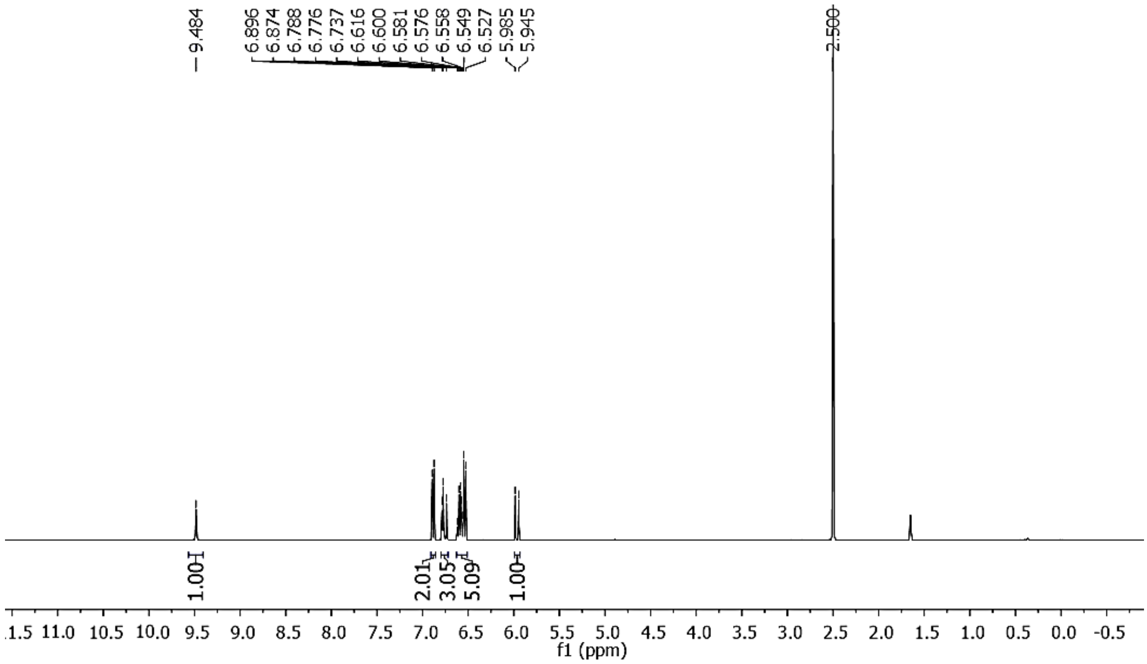

Figure S77. ^{1}H NMR (400MHz, DMSO-d_6) spectrum of 4-chlorophenylcinnamamide (**27**)

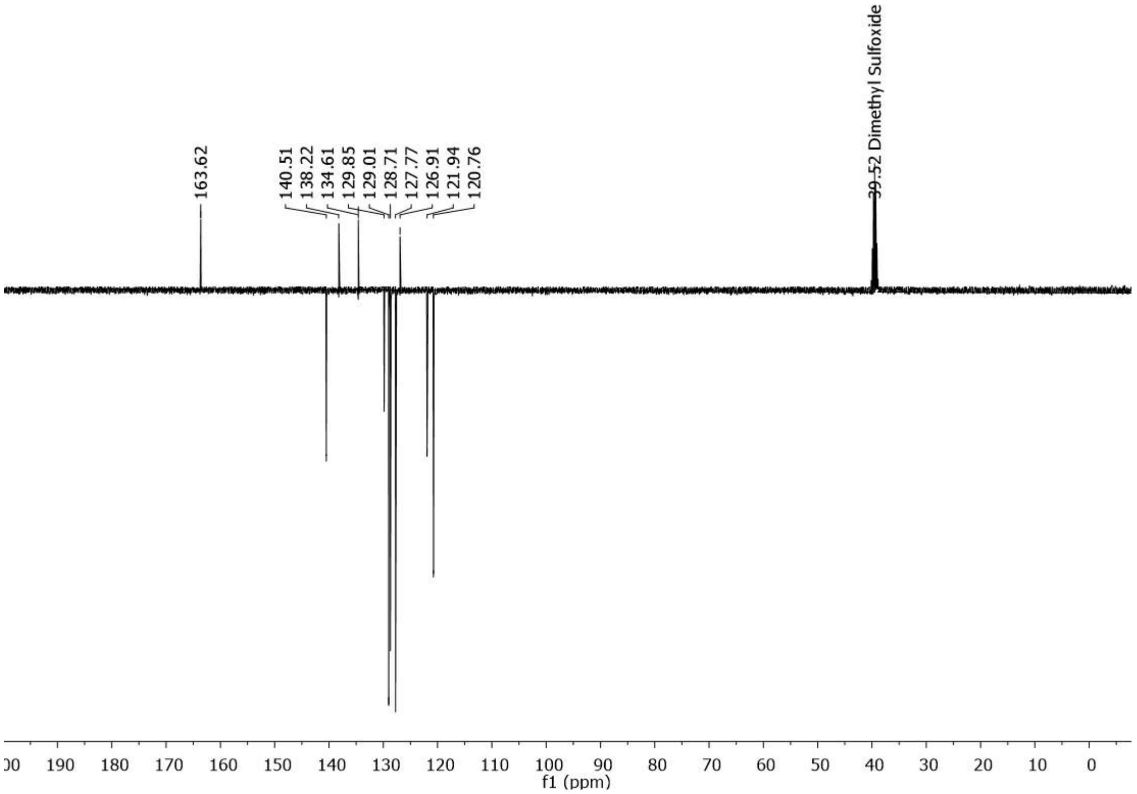

Figure S78. ^{13}C NMR (100MHz, DMSO-d_6) spectrum of 4-chlorophenylcinnamamide (**27**)

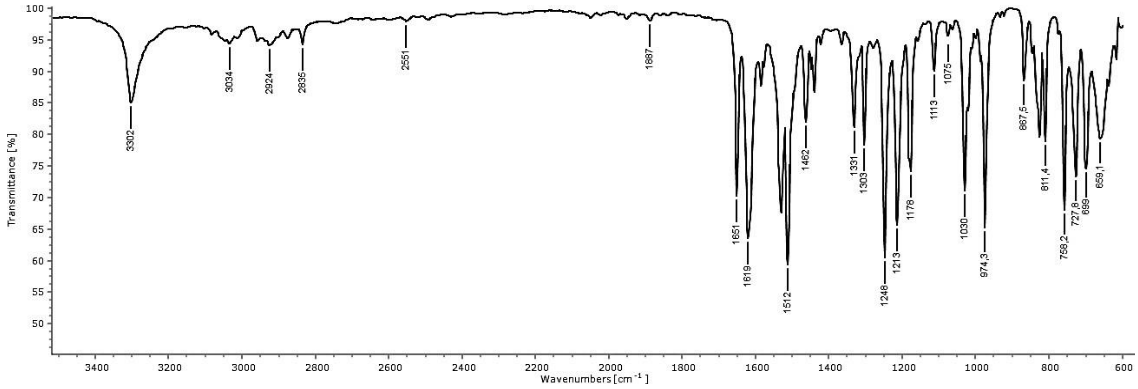

Figure S79. IR υmax (KBr, cm^{-1}) spectrum of benzylcinnamamide (**28**)

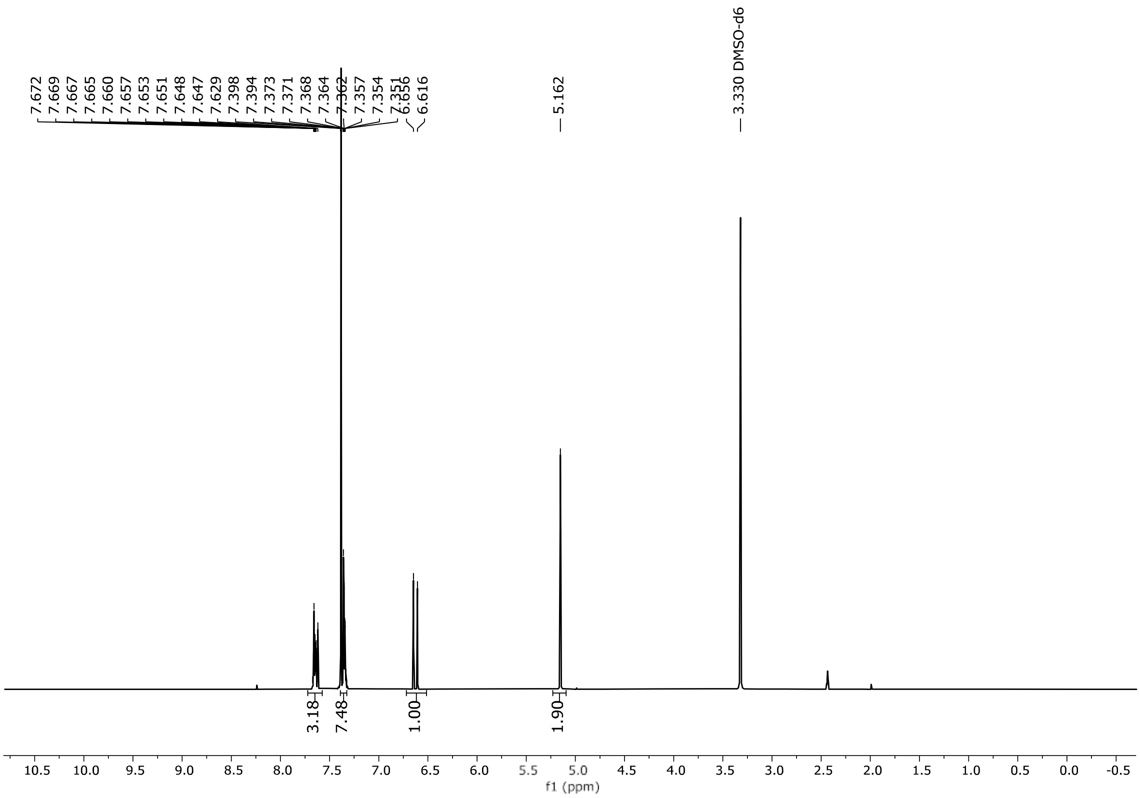

Figure S80. ^{1}H NMR (500MHz, $CDCl_3$) spectrum of benzylcinnamamide (**28**)

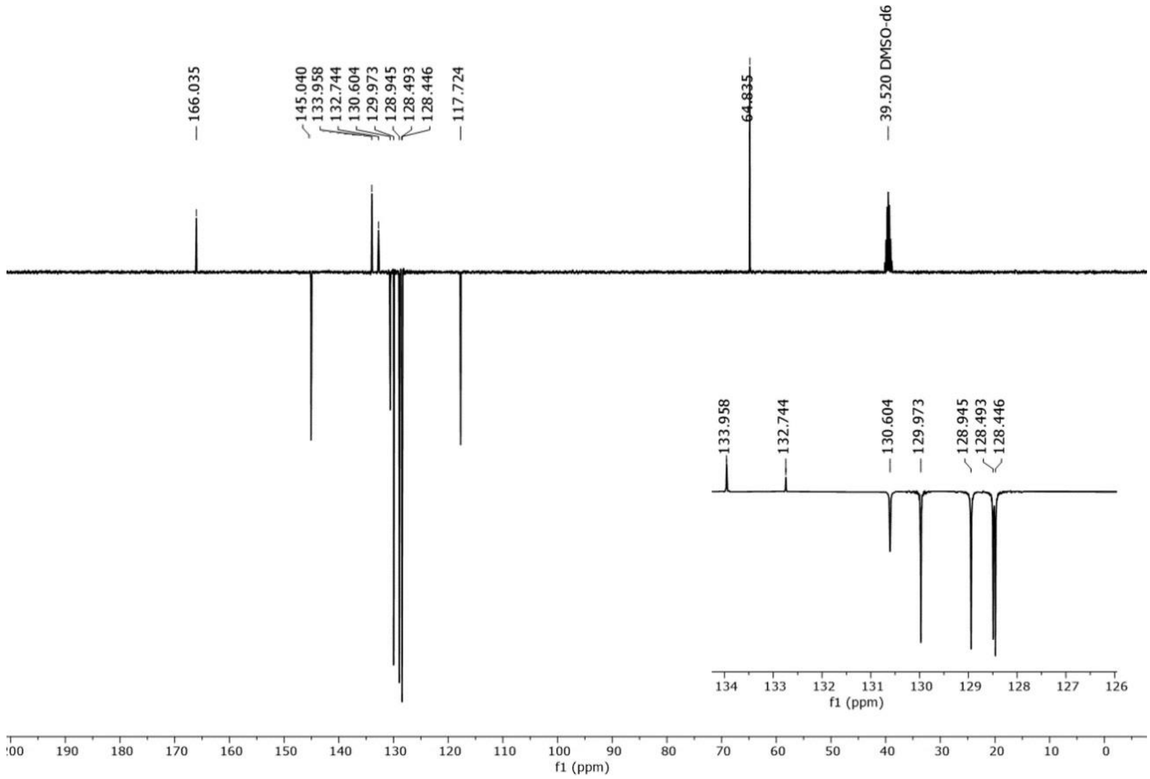

Figure S81. ^{13}C NMR (125 MHz, $CDCl_3$) spectrum of benzylcinnamamide (**28**)

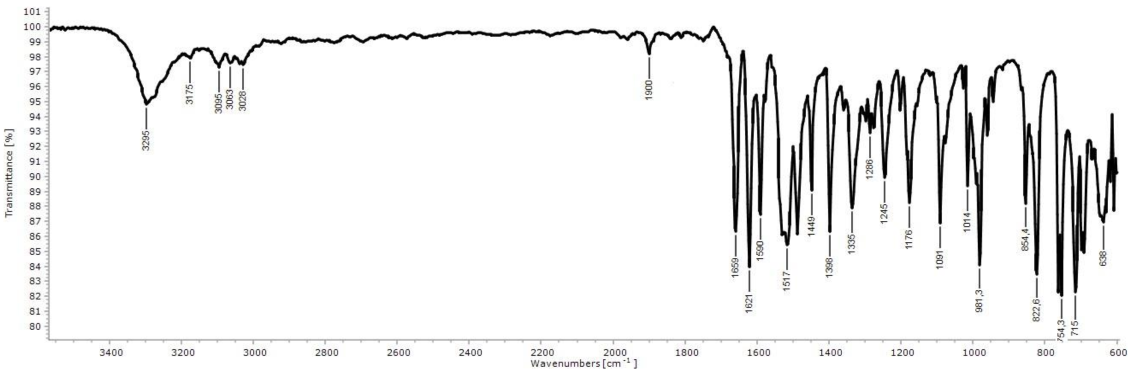

Figure S82. IR υmax (KBr, cm^{-1}) spectrum of 4-chlorobenzylcinnamamide (**29**)

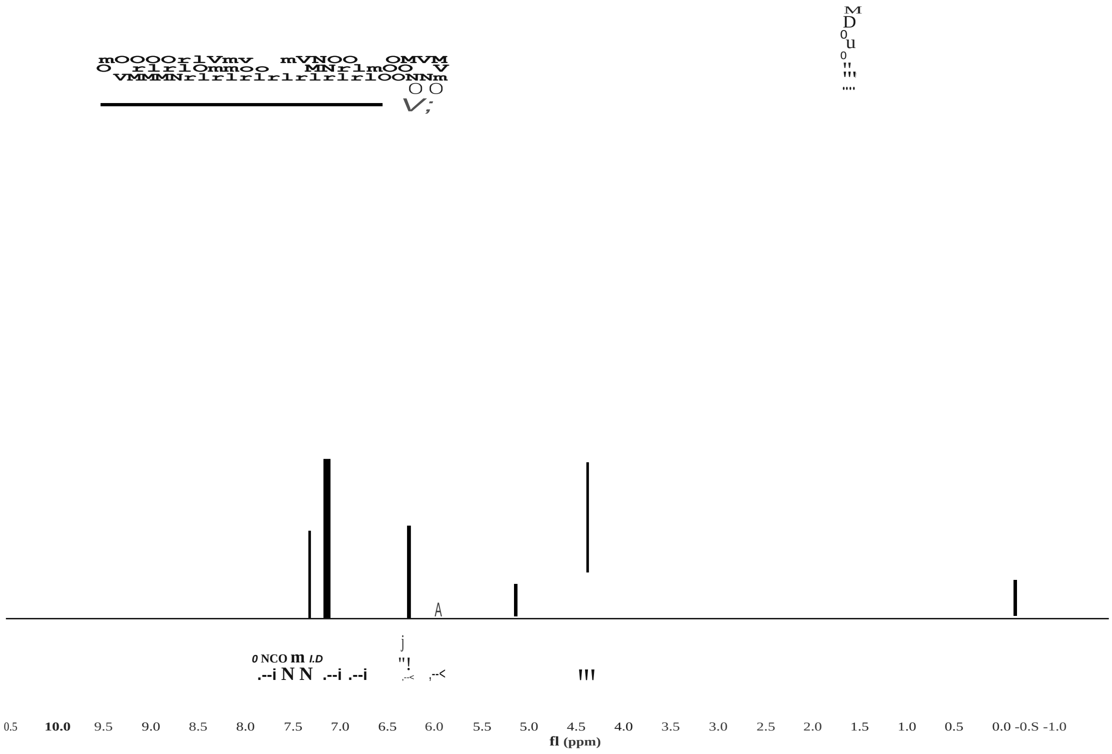

Figure S83. ^{1}H NMR (500MHz, $CDCl_3$) spectrum of 4-chlorobenzylcinnamamide (**29**)

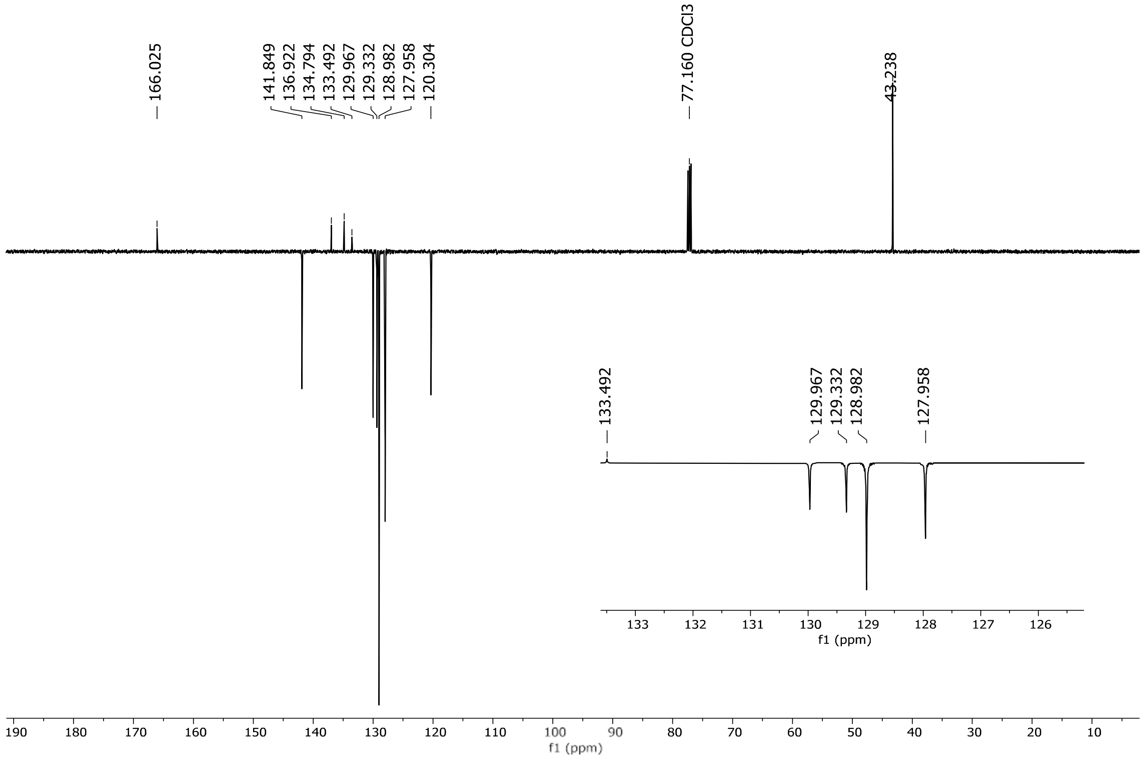

Figure S84. ^{13}C NMR (125 MHz, $CDCl_3$) spectrum of 4-chlorobenzylcinnamamide (**29**)

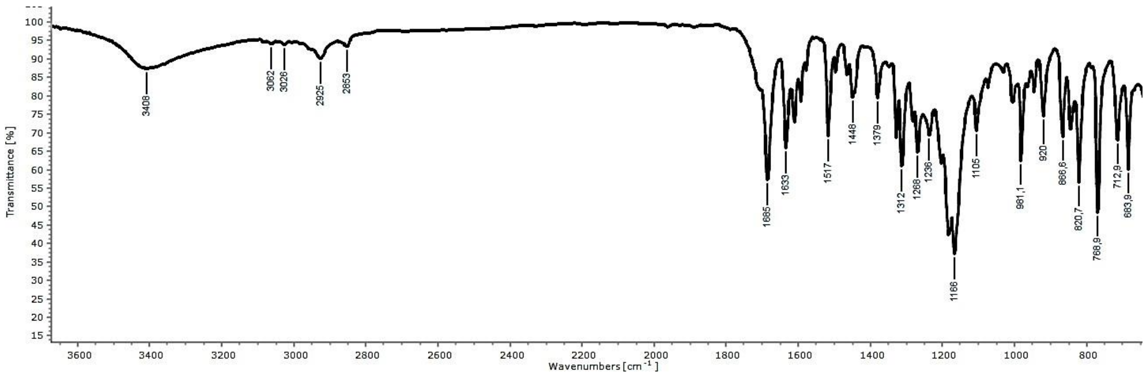

Figure S85. IR υmax (KBr, cm^{-1}) spectrum of 4-hydroxyphenylcinnamamide (**30**)

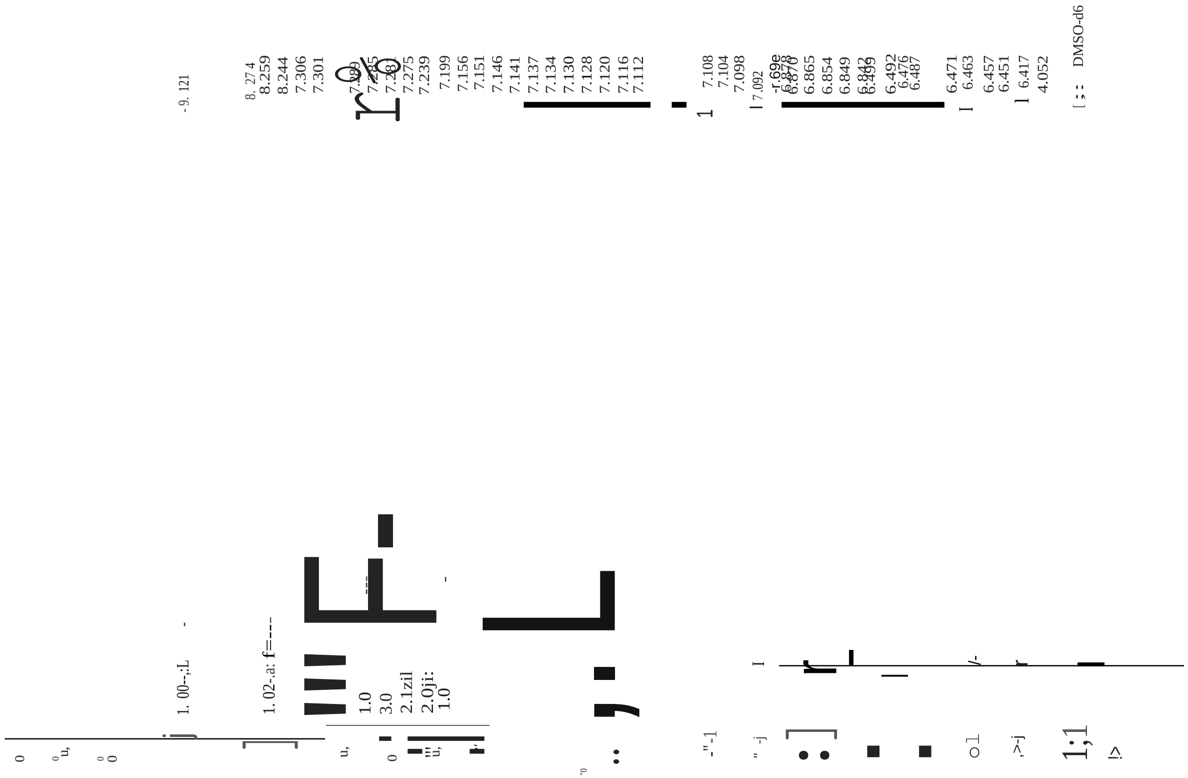

Figure S86. ^{1}H NMR (400MHz, DMSO-d_6) spectrum of 4-hydroxyphenylcinnamamide (**30**)

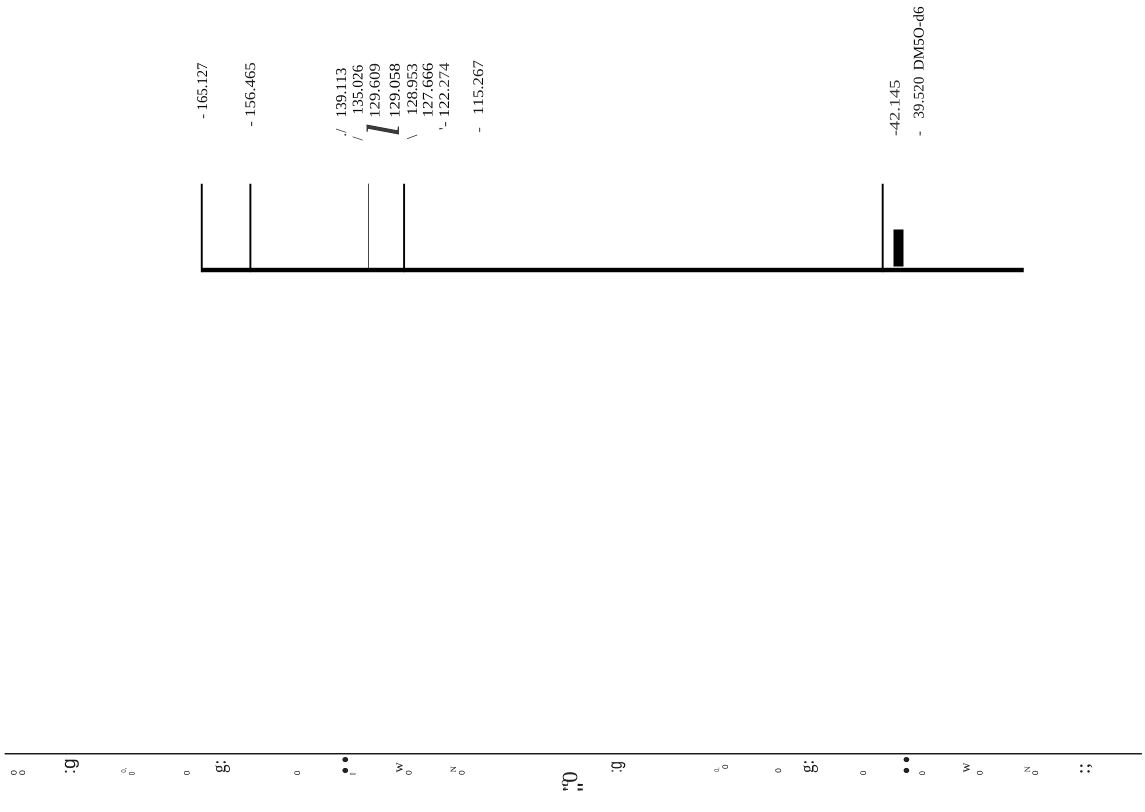

Figure S87. ^{13}C NMR (100MHz, DMSO-d_6) spectrum of 4-hydroxyphenylcinnamamide (**30**)

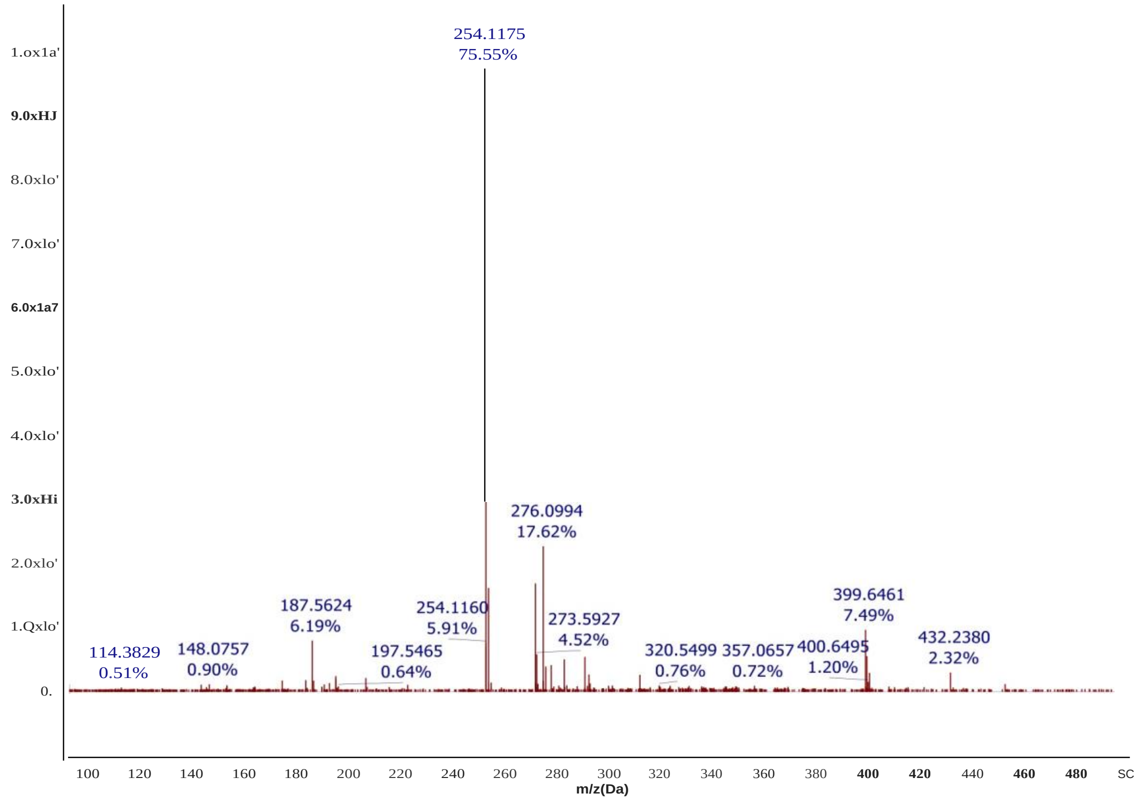

Figure S88. MALDI-TOF high resolution mass spectrum of 4-hydroxybenzylcinnamamide (**30**)

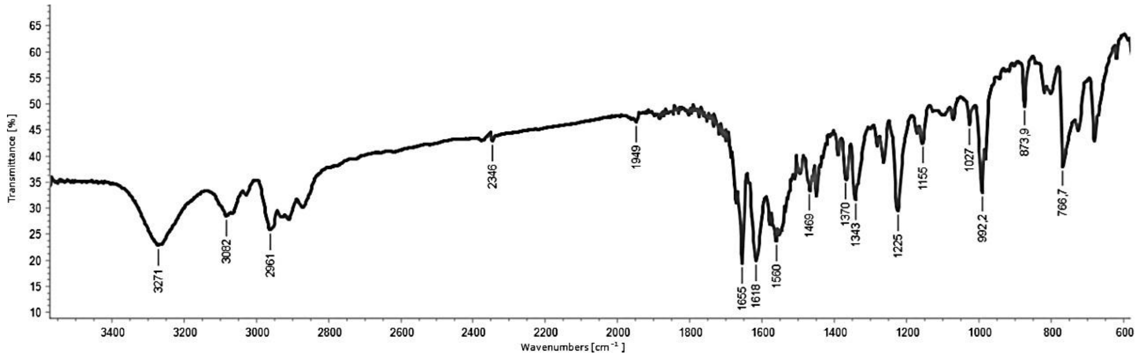

Figure S89. IR υmax (KBr, cm^{-1}) spectrum of 4-methoxyphenylcinnamamide (**31**)

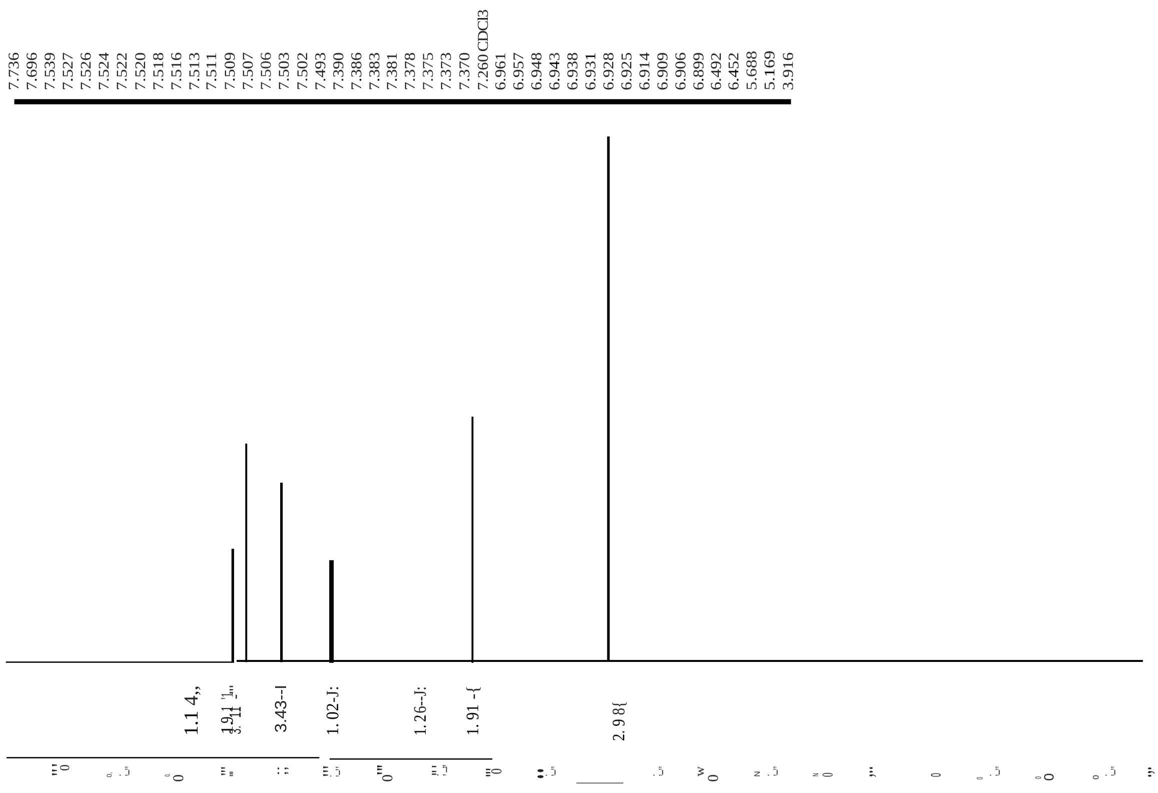

Figure S90. ^{1}H NMR (400MHz, DMSO-d$_6$) spectrum of 4-methoxyphenylcinnamamide (**31**)

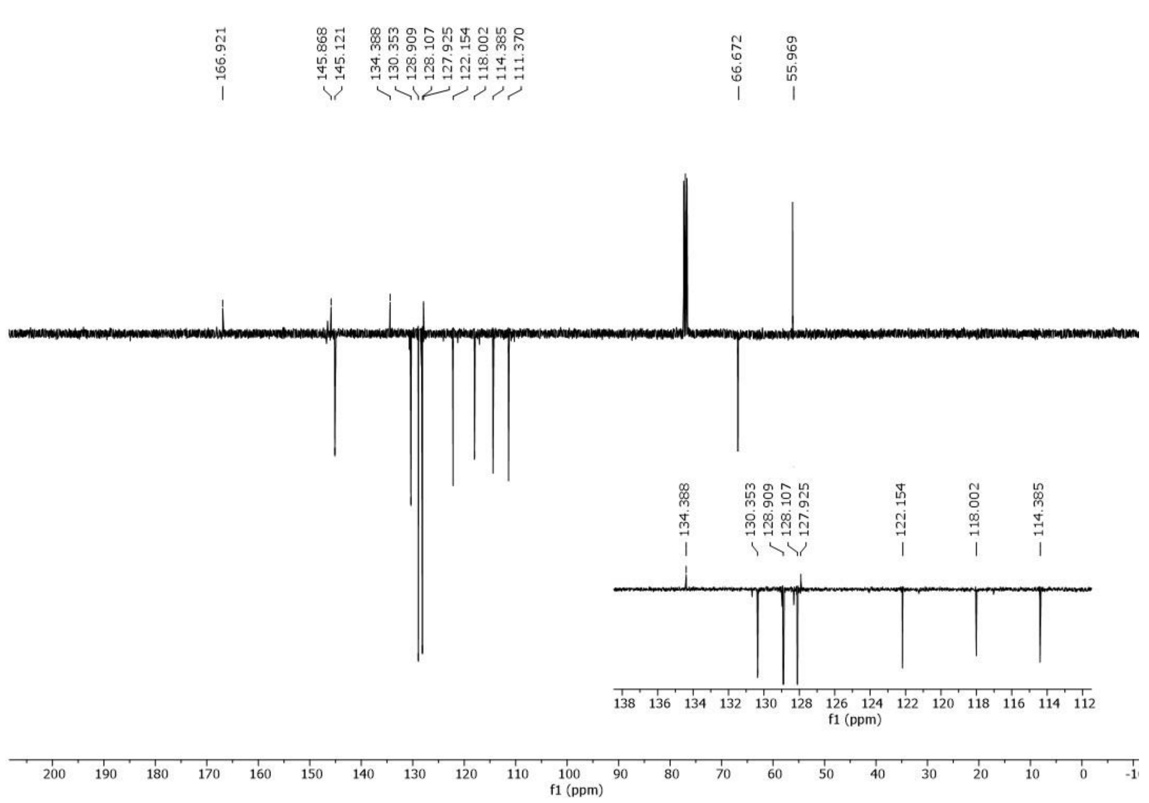

Figure S91. ^{13}C NMR (100MHz, DMSO-d$_6$) spectrum of 4-methoxyphenylcinnamamide (**31**)

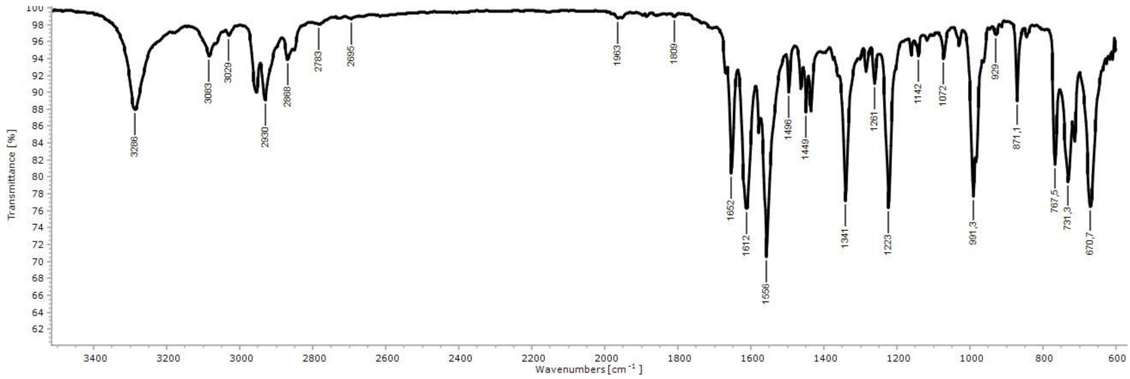

Figure S92. IR υmax (KBr, cm^{-1}) spectrum of 4-isopropylbenzylcinnamamide (**32**)

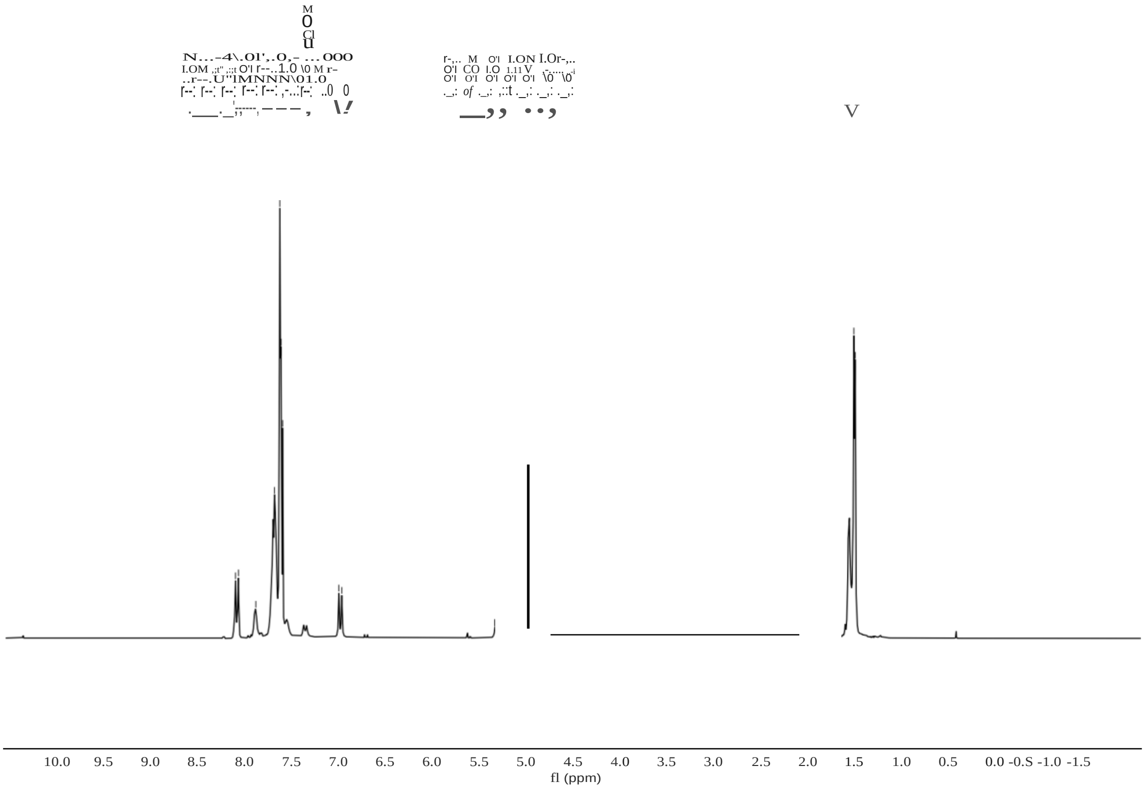

Figure S93. ^1H NMR (500MHz, $CDCl_3$) spectrum of 4-isopropylbenzylcinnamamide (**32**)

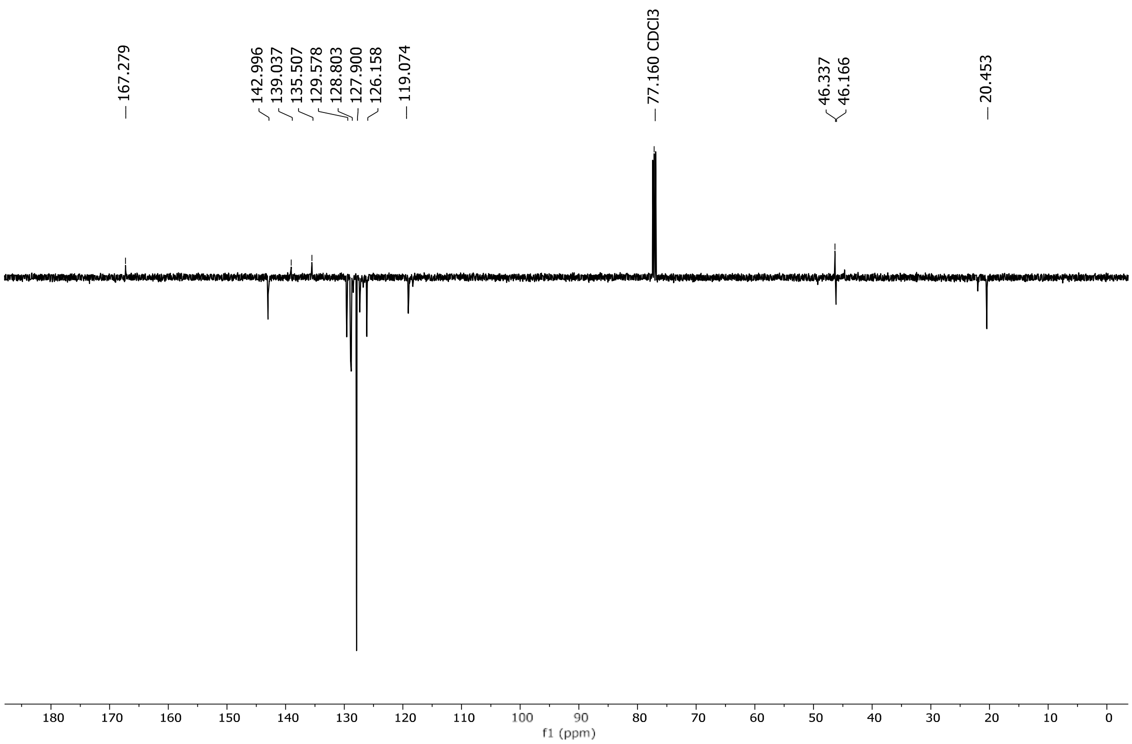

Figure S94. ^{13}C NMR (125 MHz, $CDCl_3$) spectrum of 4-isopropylbenzylcinnamamide (**32**)

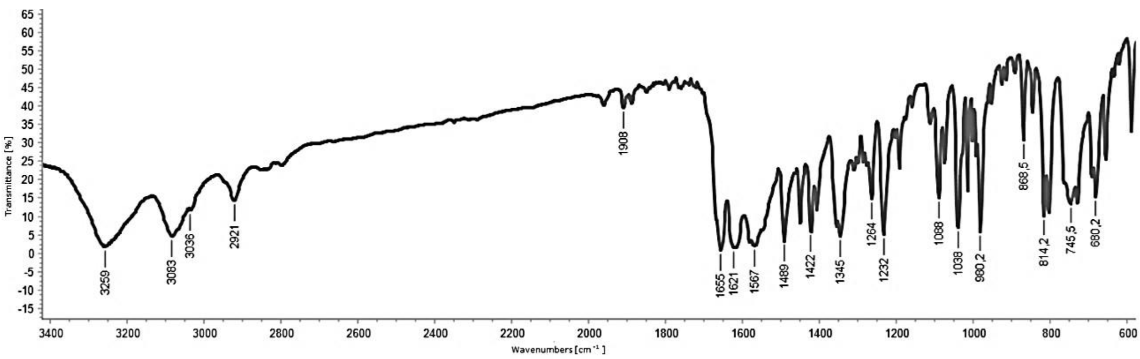

Figure S95. IR υmax (KBr, cm^{-1}) spectrum of 4-methylbenzylcinnamamide (**33**)

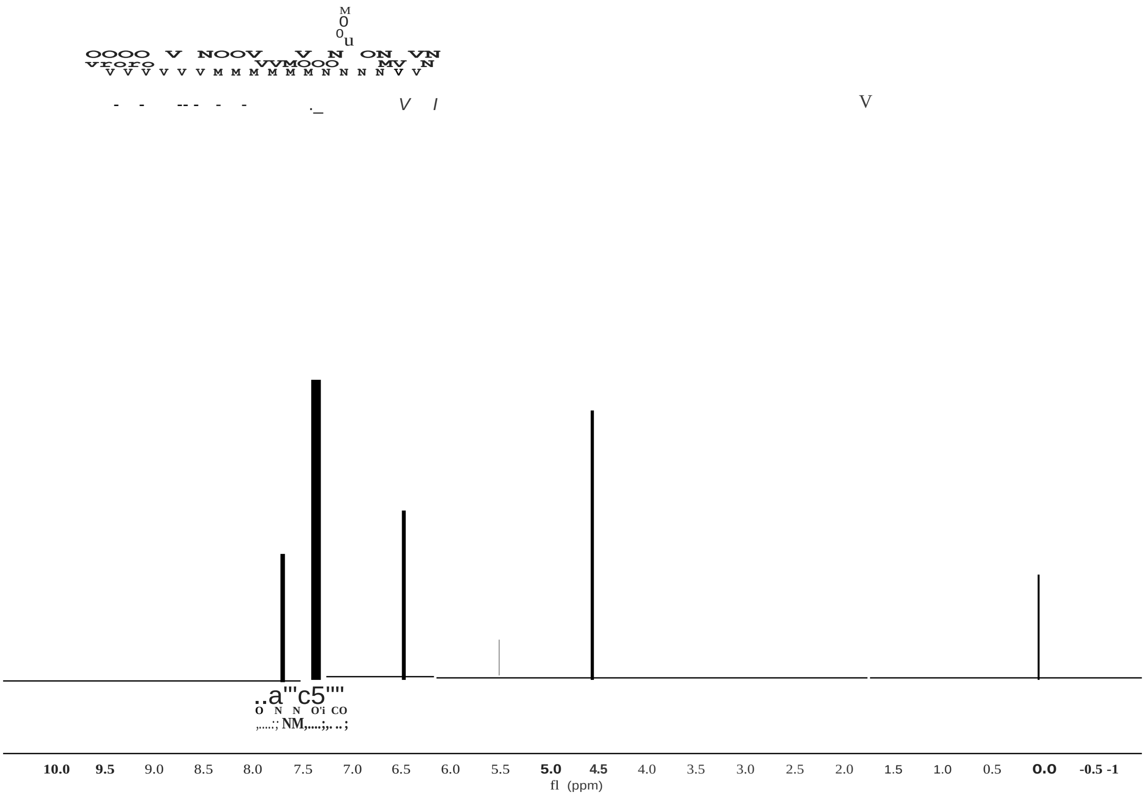

Figure S96. ^{1}H NMR (400 MHz, $CDCl_3$) spectrum of 4-methylbenzylcinnamamide (**33**)

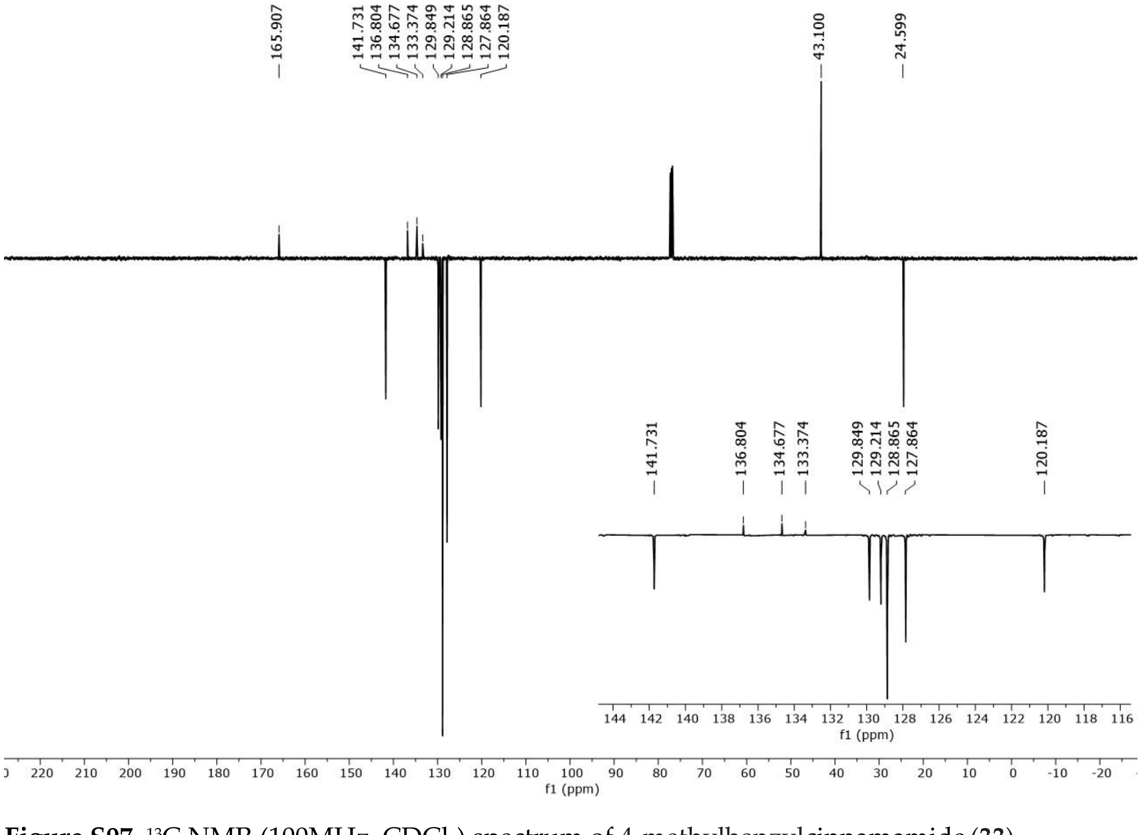

Figure S97. ^{13}C NMR (100MHz, $CDCl_3$) spectrum of 4-methylbenzylcinnamamide (**33**)

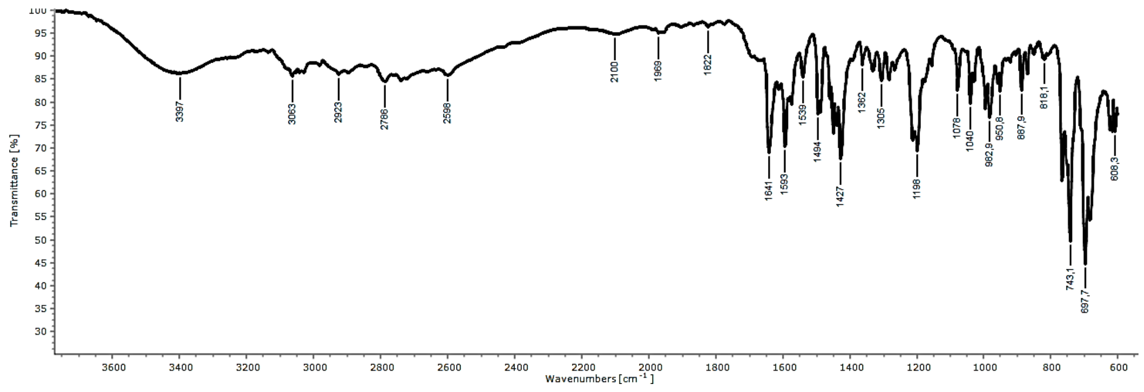

Figure S98. IR υmax (KBr, cm⁻¹) spectrum of piperonylcinnamamide (**34**)

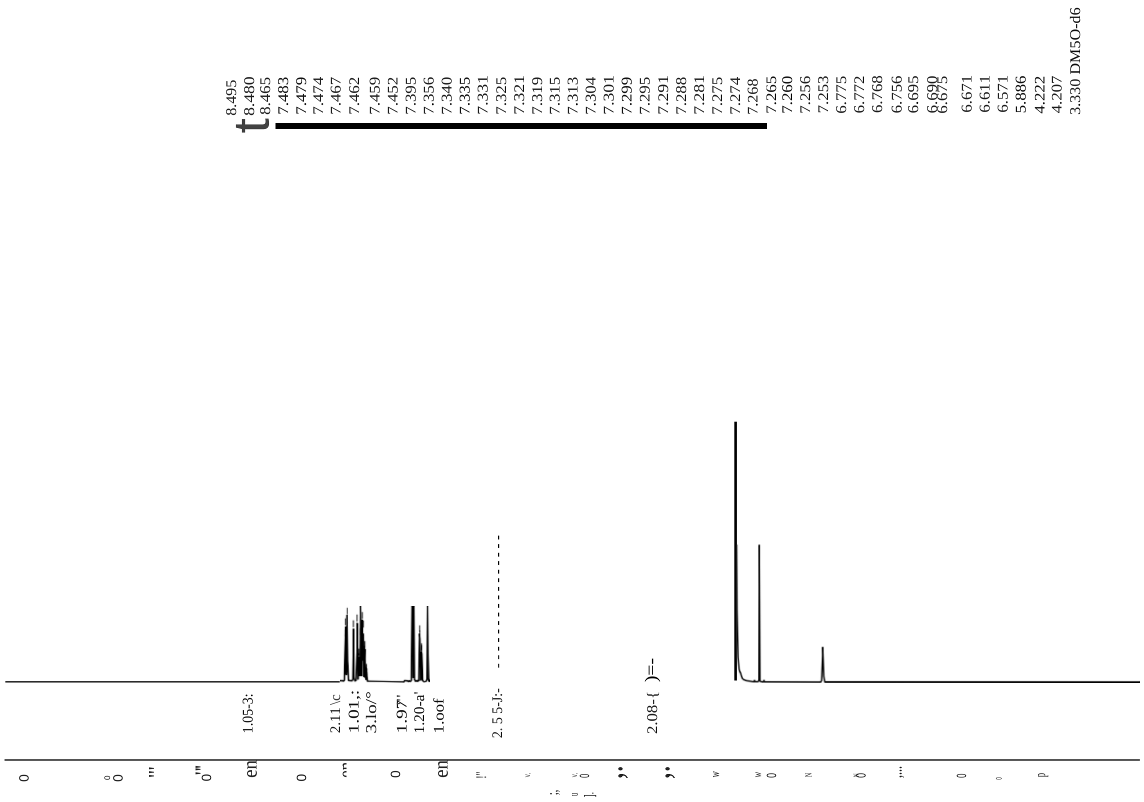

Figure S99. ^{1}H NMR (400MHz, DMSO-d_6) spectrum of piperonylcinnamamide (**34**)

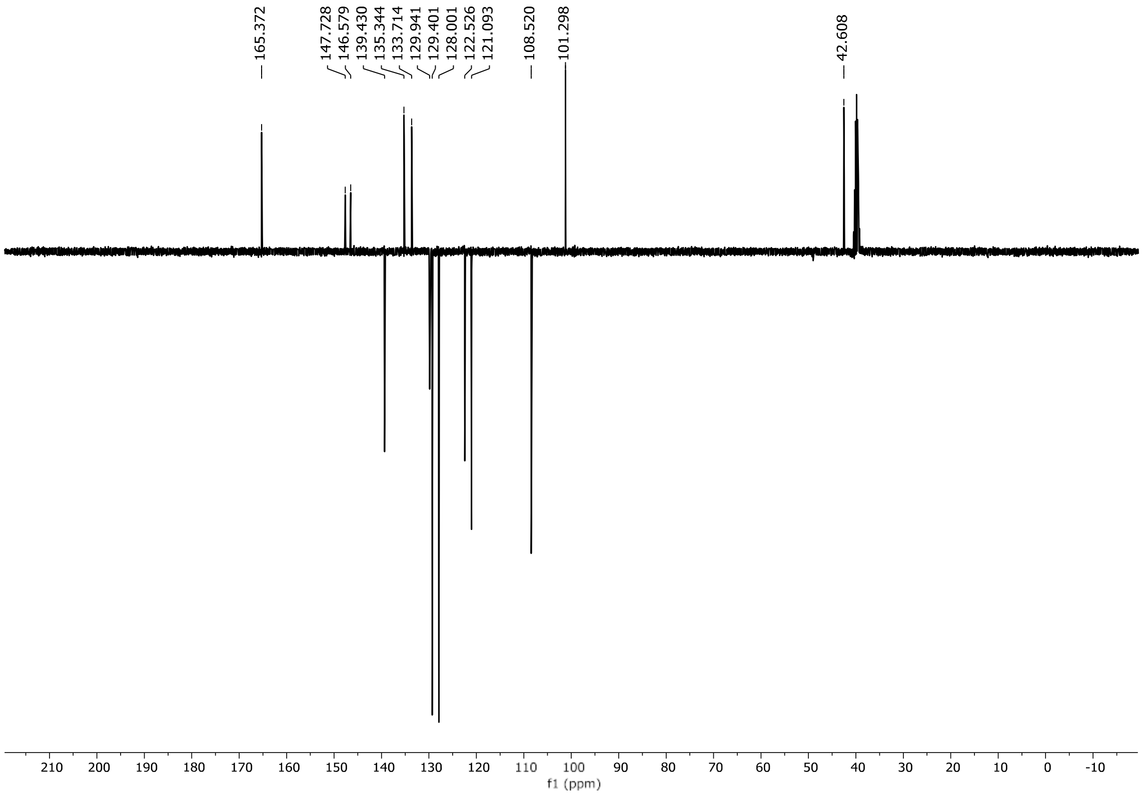

Figure S100. ^{13}C NMR (100MHz, DMSO-d_6) spectrum of piperonylcinnamamide (**34**)

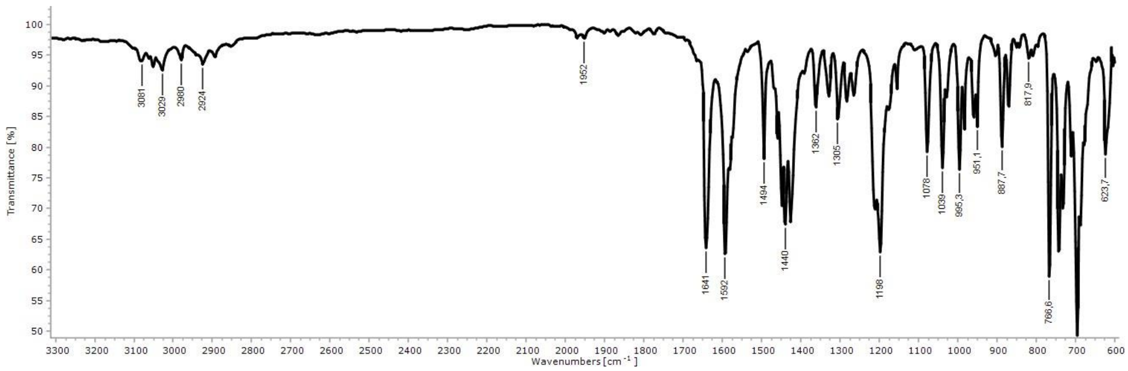

Figure S101. IR υmax (KBr, cm^{-1}) spectrum of dibenzylcinnamamide (**35**)

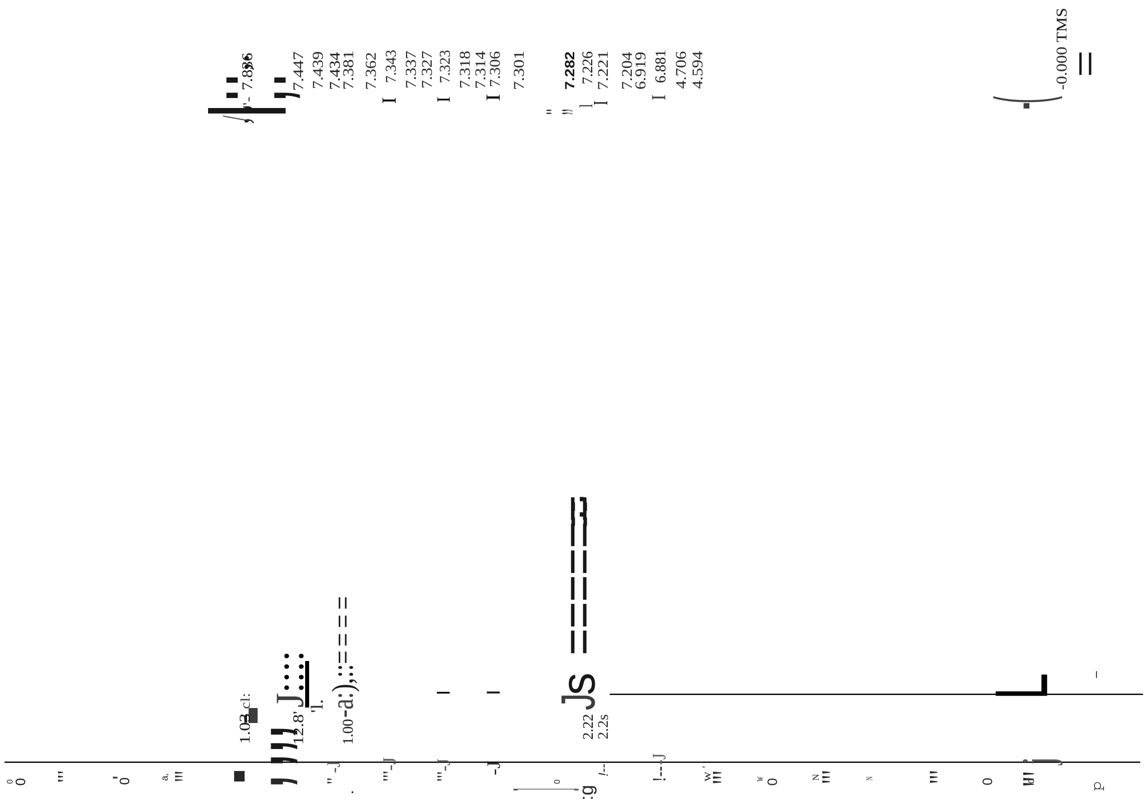

Figure S102. ^1H NMR (400MHz, $CDCl_3$) spectrum of dibenzylcinnamamide (**35**)

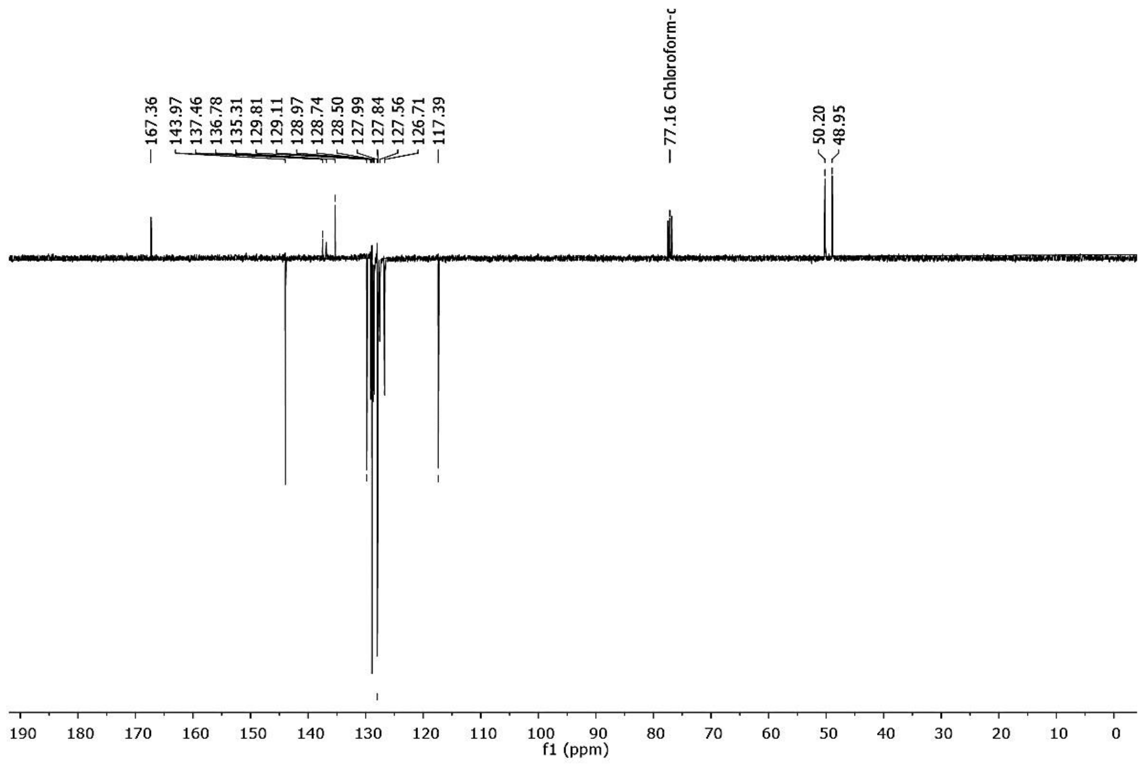

Figure S103. ^{13}C NMR (100MHz, $CDCl_3$) spectrum of dibenzylcinnamamide (**35**)

Table S1. Results of docking compound **32** to its potential targets.

Target	Pose	PLP	Z_PLP	GS	Z_GS	CS	Z_CS	ASP	Z_ASP	Aggregated Z-Score
PGFS	1	60.40	3.13	-7.79	-0.07	27.91	1.75	32.61	1.28	1.52
	2	53.94	1.62	14.74	1.02	25.91	1.32	28.92	0.62	1.14
ALDH2	1	71.49	1.86	-1.81	-0.53	33.01	1.73	39.00	2.50	1.39
	2	68.38	1.19	25.74	1.11	32.29	1.52	35.41	1.52	1.33
	3	73.79	2.35	-38.10	-2.69	34.77	2.24	37.60	2.12	1.01
CPC	1	53.12	1.37	30.45	1.29	17.93	0.53	27.70	1.40	1.15
	2	57.47	2.39	25.25	0.92	16.98	0.15	26.43	1.10	1.14
	3	50.91	0.85	28.21	1.13	21.75	2.02	23.26	0.37	1.09
AAP	1	70.10	1.20	41.78	1.66	27.45	1.30	29.12	1.23	1.35
CYPA	1	63.48	0.90	17.96	0.20	25.41	0.42	31.44	2.00	0.88
CYP2	1	78.44	3.04	30.55	1.03	31.88	2.46	33.74	2.85	2.34
	2	62.63	1.00	23.50	0.72	27.23	1.22	29.11	1.41	1.09
	3	62.00	0.91	32.49	1.12	27.05	1.17	27.75	0.99	1.05
CYP5	1	70.57	1.06	35.06	1.23	29.43	1.23	32.95	1.82	1.33
	2	72.74	1.34	31.67	1.07	29.60	1.28	29.41	0.79	1.12
	3	73.08	1.38	28.85	0.94	28.90	1.07	28.89	0.64	1.01
CYP6	1	62.53	1.86	36.74	1.63	26.03	1.56	25.65	0.12	1.29
CYP40	1	51.44	0.78	23.33	0.85	21.94	1.17	24.66	0.89	0.92
PPT (ATP)	1	57.03	1.79	36.64	1.42	23.52	1.91	20.22	1.41	1.63
PPT (Glu)	1	51.68	1.08	39.71	0.35	15.07	1.87	11.16	1.38	1.17
	2	51.87	1.11	7.71	0.22	12.20	1.19	13.44	1.92	1.11
PAH	1	79.06	2.51	39.53	1.07	32.07	2.98	29.00	-0.09	1.62

Table S2. Predicted free energies of binding (and components) of compound **32** to its predicted targets.

Target	Conformer	MM-PBSA Component							ΔG total
		VD WAALS	EEL	EPB	ENPOLAR	EDISPER	ΔG gas	ΔG solvent	
PGFS	1	-21.44	-11.67	23.35	-17.53	30.31	-33.11	36.13	3.01
	2	-13.42	-2.92	9.89	-11.51	19.85	-16.34	18.23	1.90
ALDH2	1	-43.90	-18.98	36.69	-32.83	53.57	-62.88	57.44	-5.45
	2	-42.18	-14.25	36.51	-32.16	53.79	-56.43	58.14	1.71
	3	-43.59	-21.10	39.46	-32.20	52.69	-64.69	59.95	-4.74
CPC	1	-24.41	-4.22	17.36	-18.33	31.60	-28.63	30.63	2.00
	2	-29.66	-5.85	23.13	-20.50	37.15	-35.51	39.78	4.27
	3	-32.86	-11.39	26.37	-22.59	40.54	-44.24	44.32	0.08
AAP	1	-37.78	-43.15	45.61	-30.67	48.55	-80.93	63.49	-17.44
CYPA	1	-22.29	-9.12	16.70	-18.37	28.53	-31.41	26.86	-4.55
CYP2	1	-23.19	-2.55	14.29	-18.78	31.36	-25.73	26.86	1.13
	2	-21.74	-7.77	18.21	-17.98	29.93	-29.52	30.16	0.64
	3	-27.48	-2.26	15.74	-21.48	34.79	-29.74	29.05	-0.70
CYP5	1	-20.36	-4.55	14.25	-16.19	27.28	-24.92	25.34	0.42
	2	-30.65	-1.88	17.73	-24.27	38.45	-32.53	31.91	-0.62

	3	-22.11	-7.85	16.87	-17.26	28.42	-29.96	28.03	-1.93
CYP6	1	-19.76	-5.66	15.21	-16.50	27.76	-25.42	26.46	1.05
CYP40	1	-20.60	-4.55	15.37	-16.55	27.86	-25.14	26.69	1.55
PPT (ATP)	1	-35.46	-12.06	31.30	-26.46	44.78	-47.52	49.62	2.09
PPT (Glu)	1	-52.13	-35.68	69.75	-33.56	55.56	-87.80	91.74	3.93
	2	-48.59	-34.78	66.72	-32.39	55.14	-83.37	89.47	6.10
PAH	1	-33.03	-87.08	115.50	-32.47	54.59	-120.12	137.62	17.50

References

(1) Araújo, M. O.; Pérez-Castillo, Y.; Oliveira, L. H. G.; Nunes, F. C.; Sousa, D. P. d. Larvicidal Activity of Cinnamic Acid Derivatives: Investigating Alternative Products for Aedes Aegypti L. Control. *Mol. 2021, Vol. 26, Page 61* **2020**, *26* (1), 61. https://doi.org/10.3390/MOLECULES26010061.

(2) de Morais, M. C.; Perez-Castillo, Y.; Silva, V. R.; de Souza Santos, L.; Soares, M. B. P.; Bezerra, D. P.; de Castro, R. D.; de Sousa, D. P. Cytotoxic and Antifungal Amides Derived from Ferulic Acid: Molecular Docking and Mechanism of Action. *Biomed Res. Int.* **2021**, *2021*. https://doi.org/10.1155/2021/3598000.

(3) Iranpoor, N.; Firouzabadi, H.; Riazi, A.; Pedrood, K. Regioselective Hydrocarbonylation of Phenylacetylene to α,β-Unsaturated Esters and Thioesters with Fe(CO)5 and Mo(CO)6. *J. Organomet. Chem.* **2016**, *822*, 67–73. https://doi.org/10.1016/J.JORGANCHEM.2016.01.025.

(4) Lutjen, A. B.; Quirk, M. A.; Barbera, A. M.; Kolonko, E. M. Synthesis of (E)-Cinnamyl Ester Derivatives via a Greener Steglich Esterification. *Bioorg. Med. Chem.* **2018**, *26* (19), 5291–5298. https://doi.org/10.1016/J.BMC.2018.04.007.

(5) Iranpoor, N.; Firouzabadi, H.; Riaz, A.; Pedrood, K. Regioselective Hydrocarbonylation of Phenylacetylene to α,β-Unsaturated Esters and Thioesters with Fe(CO)5 and Mo(CO)6. *J. Organomet. Chem.* **2016**, *822*, 67–73.

(6) Jakovetić, S.; Jugović, B. Z.; Gvozdenović, M. M.; Bezbradica, D. I.; Antov, M. G.; Knežević-Jugović; Mijin, D. Ž.; D., Z. Synthesis of Aliphatic Esters of Cinnamic Acid as Potential Lipophilic Antioxidants Catalyzed by Lipase B from Candida Antarctica. *Appl. Biochem. Biotechnol.* **2013**, *170*, 1560–1573.

(7) Sova, M. .; Perdih, A. .; Kotnik, M. .; Kristan, K. .; Rizner, T. L. .; Solmajer, T. .; Gobec, S. Flavonoids and Cinnamic Acid Esters as Inhibitors of Fungal 17β-Hydroxysteroid Dehydrogenase: A Synthesis, QSAR and Modelling Study. *Bioorg. Med. Chem.* **2006**, *14*, 7404–7418.

(8) Sova, M. Antioxidant and Antimicrobial Activities of Cinnamic Acid Derivatives. *Mini Rev. Med. Chem.* **2012**, *12* (8), 749–767. https://doi.org/10.2174/138955712801264792.

(9) Bisogno, F.; Mascoti, L.; Sanchez, C.; Garibotto, F.; Giannini, F.; Kurina-Sanz, M.; Enriz, R. Structure-Antifungal Activity Relationship of Cinnamic Acid Derivatives. *J. Agric. Food Chem.* **2007**, *55* (26), 10635–10640. https://doi.org/10.1021/JF0729098.

(10) Silva, R. H. N.; Andrade, A. C. M.; Nóbrega, D. F.; Castro, R. D. D.; Pessôa, H. L. F.; Rani, N.; De Sousa, D. P. Antimicrobial Activity of 4-Chlorocinnamic Acid Derivatives. *Biomed Res. Int.* **2019**, *2019*. https://doi.org/10.1155/2019/3941242.

(11) Vale, J. A. do; Rodrigues, M. P.; Lima, Â. M. A.; Santiago, S. S.; Lima, G. D. de A.; Almeida, A. A.; Oliveira, L. L. de; Bressan, G. C.; Teixeira, R. R.; Machado-Neves, M. Synthesis of Cinnamic Acid Ester Derivatives with Antiproliferative and Antimetastatic Activities on Murine Melanoma Cells. *Biomed. Pharmacother.* **2022**, *148*, 112689. https://doi.org/10.1016/J.BIOPHA.2022.112689.

(12) OSUKA, A. .; HANASAKI, Y. .; SUZUKI, H. Synthesis of α, Β- Unsaturated Carboxamides Using Dialkyltelluronium Carbamoylmethylide. *ChemInform* **1998**, *19* (4), no-no.

(13) Shuldburg, S.; Carroll, J. Scaffolding Students' Skill Development by First Introducing Advanced Techniques through the Synthesis and 15N NMR Analysis of Cinnamamides. *J. Chem. Educ.* **2017**, *94* (12), 1974–1977. https://doi.org/10.1021/ACS.JCHEMED.7B00279/ASSET/IMAGES/MEDIUM/ED-2017-002798_0006.GIF.

(14) Weidner-Wells, M. A.; Fraga-Spano, S. A.; Turchi, I. J. Unusual Regioselectivity of the Dipolar

Cycloaddition Reactions of Nitrile Oxides and Tertiary Cinnamides and Crotonamides(1). *J. Org. Chem.* **1998**, *63* (18), 6319–6328. https://doi.org/10.1021/JO9807621.
(15) Nimse, S. B.; Pal, D.; Mazumder, A.; Mazumder, R. Synthesis of Cinnamanilide Derivatives and Their Antioxidant and Antimicrobial Activity. *J. Chem.* **2015**, *2015*. https://doi.org/10.1155/2015/208910.
(16) Chen, B.; Wu, X. F. Synthesis of Linear α,β-Unsaturated Amides from Isocyanates and Alkenylaluminum Reagents. *Synlett* **2020**, *31* (8), 788–792. https://doi.org/10.1055/S-0037-1610753/ID/JR000-1008.
(17) Morcillo, S. P.; Álvarez De Cienfuegos, L.; Mota, A. J.; Justicia, J.; Robles, R. Mild Method for the Selective Esterification of Carboxylic Acids Based on the Garegg-Samuelsson Reaction. *J. Org. Chem.* **2011**, *76* (7), 2277–2281. https://doi.org/10.1021/JO102395C/SUPPL_FILE/JO102395C_SI_001.PDF.
(18) ZHU J; Zhu, H. .; Kobamoto, N. .; Yasuda, M. Fungitoxic and Phytotoxic Activities of Cinnamic Acid Ester Sand Amides. *J. Pestic. Sci.* **2001**, *25* (3), 263–266.
(19) O'BRIEN, M. J. .; MCNULTY, T. F. .; CHAN, K. P. Composition and Method for Use in Three Dimensional Printing. 9,873, 2018.
(20) Knuniants, I. L.; Gambarian, N. P. Determination of the Strength of the Bond between Radicals and Sulfur in Unsymmetric Sulfides by the Method of Destructive Bromination. *Bull. Acad. Sci. USSR Div. Chem. Sci.* **1958**, *7* (10), 1176–1184. https://doi.org/10.1007/BF00914947/METRICS.
(21) Duangkamol, C.; Jaita, S.; Wangngae, S.; Phakhodee, W.; Pattarawarapan, M. An Efficient Mechanochemical Synthesis of Amides and Dipeptides Using 2,4,6-Trichloro-1,3,5-Triazine and PPh3. *RSC Adv.* **2015**, *5* (65), 52624–52628. https://doi.org/10.1039/C5RA10127A.
(22) Wang, X.; He, L.; Li, Z.; Wang, W.; Liu, J. SmI3-Catalyzed Addition of Amines to α,β-Unsaturated N-Acylbenzotriazoles. *http://dx.doi.org/10.1080/00397910802431180* **2009**, *39* (5), 819–829. https://doi.org/10.1080/00397910802431180.
(23) Bouali, J.; Hamri, S. Design, Synthesis and Evaluation of N-Aryl Carboxamide Derivatives as Potential Anti-Proliferative Effect on the Pulmonary Artery Smooth Muscle Cells. *Artic. Res. J. Pharm. Biol. Chem. Sci.* **2015**.
(24) Saito, Y.; Ouchi, H.; Takahata, H. Carboxamidation of Carboxylic Acids with 1-Tert-Butoxy-2-Tert-Butoxycarbonyl-1,2-Dihydroisoquinoline (BBDI) without Bases. *Tetrahedron* **2008**, *64* (49), 11129–11135. https://doi.org/10.1016/J.TET.2008.09.094.
(25) Allen, C. L.; Chhatwal, A. R.; Williams, J. M. J. Direct Amide Formation from Unactivated Carboxylic Acids and Amines. *Chem. Commun.* **2011**, *48* (5), 666–668. https://doi.org/10.1039/C1CC15210F.
(26) Martínez-Soriano, P. A.; Macías-Pérez, J. R.; María Velázquez, A.; del Carmen Camacho-Enriquez, B.; Pretelín-Castillo, G.; Ruiz-Sánchez, M. B.; Abrego-Reyes, V. H.; Villa-Treviño, S.; Angeles, E. Solvent-Free Synthesis of Carboxylic Acids and Amide Analogs of CAPE (Caffeic Acid Phenethyl Ester) under Infrared Irradiation Conditions. **2015**, *5*, 81–91. https://doi.org/10.4236/gsc.2015.52011.
(27) Barajas, J. G. H.; Méndez, L. Y. V.; Kouznetsov, V. V.; Stashenko, E. E. Efficient Synthesis of New N-Benzyl- or N-(2-Furylmethyl)Cinnamamides Promoted by the "green" Catalyst Boric Acid, and Their Spectral Analysis. *Synthesis (Stuttg).* **2008**, No. 3, 0377–0382. https://doi.org/10.1055/S-2008-1032039/ID/8.
(28) Perez-Castillo, Y.; Montes, R. C.; da Silva, C. R.; Neto, J. B. de A.; Dias, C. da S.; Duarte, A. B. S.; Júnior, H. V. N.; de Sousa, D. P. Antifungal Activity of N-(4-Halobenzyl)Amides against Candida Spp. and Molecular Modeling Studies. *Int. J. Mol. Sci.* **2022**, *23* (1). https://doi.org/10.3390/ijms23010419.
(29) Khaldoun, K.; Safer, A.; Saidi-Besbes, S.; Carboni, B.; Le Guével, R.; Carreaux, F. An Efficient Solvent-Free Microwave-Assisted Synthesis of Cinnamamides by Amidation Reaction Using Phenylboronic Acid/Lewis Base Co-Catalytic System. *Synth. J. Synth. Org. Chem.* **2019**, *51* (20), 3891–3900. https://doi.org/10.1055/S-0039-1690132.
(30) Yasui, Y.; Tsuchida, S.; Miyabe, H.; Takemoto, Y. One-Pot Amidation of Olefins through Pd-Catalyzed Coupling of Alkylboranes and Carbamoyl Chlorides. *J. Org. Chem.* **2007**, *72* (15), 5898–5900. https://doi.org/10.1021/JO070724U/SUPPL_FILE/JO070724USI20070517_071910.PDF.
